# Catalyst Potential Prescribes Intermediate Coverages in Thermocatalytic Gluconic Acid Oxidation on Pt Nanoparticles

**DOI:** 10.1021/jacs.5c22029

**Published:** 2026-05-01

**Authors:** William Thomas Broomhead, Minju Chung, Karl O. Albrecht, David W. Flaherty

**Affiliations:** 1 School of Chemical and Biomolecular Engineering, 1372 Georgia Institute of Technology, Atlanta, Georgia 30032, United States; 2 ADM Research and Development Center, Archer Daniels Midland Company, Decatur, Illinois 62521, United States

## Abstract

Thermocatalytic aqueous aerobic oxidation reactions occur as kinetically coupled processes that resemble two electrocatalytic half-reactions operating at open circuit. We leverage this concept to reveal a single set of elementary steps that describe not only the fundamental relationships between steady-state rates but also catalyst electrode potentials at open circuit (*E*_cat_) during reactions among gluconic acid (GNA) and O_2_ on Pt surfaces at relevant conditions (0.03–0.95 M GNA, 20–2800 kPa O_2_, 333–363 K). Irreversible O_2_ reduction and GNA oxidation half-reactions couple via cycles that produce and consume surface hydroxyl moieties (HO*) on Pt sites, with kinetically relevant C–H bond scission that proceeds through heterolytic transition states (e.g., [*^–^O­(H)···H^+^···C­(H)­(C_5_H_9_O_6_)-O­(H)*]^⧧^). Observed turnover rates depend sensitively on the fractional coverages of both the O_2_- and GNA-derived intermediates. *In situ* measurements of *E*_cat_ provide needed mechanistic insight because these values appear uniquely sensitive to HO* coverages, which increase monotonically with increasing molar ratios of O_2_ to GNA. Notably, *E*_cat_ does not vary with the coverage of other surface intermediates. Intermittent exclusion of O_2_ from the reactor decreases *E*_cat_ to values below the point of zero charge (400 mV_RHE_) and, nonintuitively, yields increased reaction rates upon reintroduction of O_2_ due to the desorption of inhibiting GNA-derived carboxylate species. This strategy controls *E*_cat_ (and reactant coverages) in ways that increase reactor productivities by 50% or more when they are averaged over modulation cycles. These findings reveal the critical role of *E*_cat_ in setting the coverages of reactive intermediates and the ensuing impacts on rates during redox thermocatalysis.

## Graphical abstract

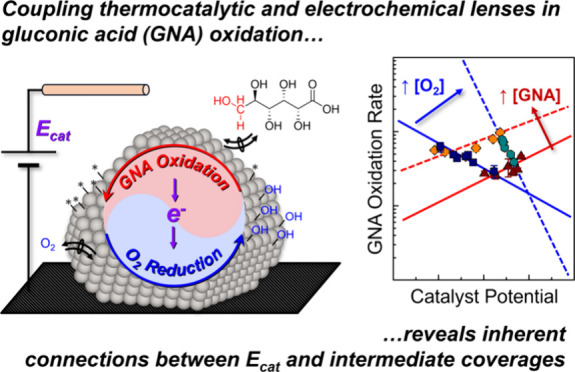

## 1 Introduction

Aqueous aerobic alcohol partial oxidation reactions convert sustainable biomass feedstocks (e.g., glucose) into commodity chemicals such as aldehydes, ketones, and carboxylic acids.^1–5^ Oxygen derived surface intermediates (e.g., O*, HO*) facilitate these reactions on heterogeneous metal catalysts (e.g., Pt, Pd, Ru, and Au), where α–C-H bond scission of the alcohol reagent typically limits rates.^6–10^ For terminal alcohols (RCH_2_OH, R = H, CH_3_, etc.), net partial oxidation reactions

$$2{\mathrm{RCH}}_{2}\mathrm{OH}+O_{2}\to 2\mathrm{RCHO}+2H_{2}O$$ 1

occur as two redox half-reactions of O_2_ reduction (eq 2a) and alcohol oxidation (eq 2b), interconnected by a surface oxygen species (e.g., HO*):

$$O_{2}+2H_{2}O+4*\to 4\mathrm{HO*}$$ 2a

$$2{\mathrm{RCH}}_{2}\mathrm{OH}+4\mathrm{HO*}\to 2\mathrm{RCHO}+4H_{2}O+4*$$ 2b

Similarly, the net reactions occur as two electrocatalytic redox half-reactions of O_2_ reduction (eq 3a) and alcohol oxidation (eq 3b), facilitated by electron transfer:

$$O_{2}+2H_{2}O+4e^{-}\to 4{\mathrm{HO}}^{-}$$ 3a

$$2{\mathrm{RCH}}_{2}\mathrm{OH}+4{\mathrm{HO}}^{-}\to 2\mathrm{RCHO}+4H_{2}O+4e^{-}$$ 3b

The thermo- and electrocatalytic approaches of decomposing net partial oxidation reactions parallel one another when the active sites (asterisks) facilitate electron transfer through the conductive catalyst substrate. However, high fractional coverages of intermediates convolute detailed mechanistic interpretations for practical operating conditions that involve elevated temperatures and pressures. These complications increase for reactions of large multifunctional molecules that bind to surfaces with diverse configurations. Experimentally unraveling this complexity and guiding catalytic design requires decoupling contributions from the two (or more) redox half-reactions from differences in the surface coverages of reactive intermediates.

Mixed potential theory offers one method to examine the fundamental connections between thermocatalytic redox reactions and electrocatalytic analogs. The elementary steps comprising eqs 3a–b balance such that the net current density (*i*_net_) equals zero during steady-state thermocatalytic operation:

$$i_{\mathrm{net}}=\sum i_{\mathrm{reduction}}+\sum i_{\mathrm{oxidation}}=0$$ 4

A traditional approach that originates from corrosion science^11–13^ involves assessing the current densities of the two half-reactions from independent linear sweep voltammetry experiments and reconstructing the relationship required to achieve zero net current *ex post facto*. This approach successfully captures the thermocatalytic rates and catalyst electrode potential (*E*_cat_) at open circuit for several oxidation and hydrogenation reactions of simple reagents (e.g., oxidation of monofunctional alcohols and aldehydes,^14–19^ HCOOH,^20^ and H_2_,^21^ and hydrogenation of nitrate groups^22–24^ ) when occurring on metal nanoparticles.

This methodology fails frequently, however, for catalytic systems that involve reactions of larger and multifunctional substrates for two possible reasons. First, such molecules adsorb with diverse mixed adsorbate structures and configurations that depend strongly on reactant concentrations.^6^ Consequently, surface coverages achieved in an independent electrocatalytic assessment of each half-reaction diverge sharply. Second, the total number of relevant half-reactions and their individual potential dependencies result in surface reactions that do not follow simple Butler–Volmer expressions. One example that illustrates these challenges includes ethanol oxidation on Pd/C (0.8 M C_2_H_5_OH, 100 kPa O_2_, 323 K),^15^ where independently measured ethanol oxidation current densities fail to account for the coverages from competitive oxygen adsorption. Furthermore, common electrochemical apparatuses need ambient pressure, modest temperatures (less than 338 K), and a buffer salt to assess the current densities by linear sweep voltammograms, which limit the predictive power of mixed potential theory to a narrow range of conditions.

Still, electroanalytical methods can offer unique insight into understanding rates and selectivities of thermocatalytic conversions (i.e., in the absence of an externally applied potential), and these appear frequently in efforts to construct conceptual bridges between the electrocatalysis and thermal catalysis communities. These efforts appear to succeed for a few chemistries to date;^14,15,17,18,20–22,24–28^ however, changes in coverages and the dominant heterolytic redox pairs limit these connections. As one illustration, the aerobic oxidation of glucose over Pt nanoparticles presents multiple complexities that limit the fidelity of predictions derived from values of *E*_cat_ at open circuit for reaction kinetics at industrially relevant conditions (0.05–1 M glucose, 20–3000 kPa O_2_, 353 K).^6^ First, the dominant redox pairs that determine *E*_cat_ depend strongly on thermodynamic activities of the reactants and coverages of surface intermediates. For example, the O_2_ reduction half-reactions contribute to *E*_cat_ when glucose concentrations remain below 1 M but become irrelevant for 1 M glucose. Second, the oxidation products (e.g., gluconic and guluronic acids) inhibit rates strongly by saturating Pt surface sites. Consequently, rates, coverages, and pH sense local glucose conversion. Collectively, these findings demonstrate the need to consider coverages of reactive species (i.e., reactants, intermediates, and products) when relating *E*_cat_ to turnover rates, barriers, and elementary kinetic parameters.

Here, we combine thermocatalytic kinetic analysis with open-circuit potentiometry to capture a single set of thermo- and electrocatalytic elementary steps in polyol oxidation reactions on Pt surfaces in a trickle-bed reactor. In this approach, gluconic acid (GNA, C_6_H_12_O_7_) serves as a model polyol substrate since GNA oxidation to guluronic acid (GLA) limits glucose-to-glucaric acid cascade reactions.^2,4^ We propose a plausible sequence of elementary steps that capture the kinetic dependencies on both GNA- and O_2_ as well as predict changes in *E*_cat_ over a wide range of conditions with high fractional coverages of both GNA- and O_2_-derived reactive intermediates (0.025–0.95 M GNA, 20–2800 kPa O_2_, 353 K). *E*_cat_ values depend monotonically on the O_2_-to-GNA ratio with a slope corresponding to the elementary electrocatalytic transfer coefficients, but do not directly prescribe the rates, instead capturing the coverages of polarized surface species (i.e., HO^–^*). We further leverage the electrocatalytic insight to manipulate the surface coverages: oxidation rates on Pt decrease over time as the coverages of strongly bound anionic surface intermediates increase, but by removing O_2_ from the feed and decreasing *E*_cat_, these intermediates desorb *via* electrostatic charge repulsion to regenerate Pt-active sites. Taken together, these findings reveal fundamental connections between thermo- and electrocatalysis and the critical role of *E*_cat_ in setting the coverages of reactive intermediates.

## 2 Experimental Methods

### 2.1 Chemicals, Catalyst Materials, and Catalyst Characterizations

The following chemicals were used directly without any additional purification: δ-gluconolactone (Aldrich 99%), DI H_2_O (18.2 MΩ cm, <5 ppb organics), 1,3-butanediol (Aldrich 99%), *N*,*N*-dimethylformamide (DMF, Aldrich 99.8%), N,O-bis­(trimethylsilyl)­trifluoroacetamide (BSTFA, with 1% trimethylchlorosilane, Thermo Scientific), γ-butyrolactone (Aldrich 99%), absolute ethanol (Fisher), deuterium oxide (D_2_O, Oakwood Chemical), γ-butyrolactone-D_6_ (Aldrich, 98 atom % D), H_2_^18^O (Aldrich, 99 atom % ^18^O), potassium chloride (Aldrich, 99%), isopropanol (Fisher, laboratory grade), perchloric acid (Aldrich, 70% in H_2_O, 99.999% trace metals), sodium perchlorate (Aldrich, 98%), H_2_ (Airgas, UHP grade), Ar (Airgas, UHP grade), O_2_ (Airgas, UHP grade), and CO (10% CO in He, Airgas certified standard). In addition to those mentioned above, the following chemicals were diluted in DI water and used without additional purification for gas chromatograph calibrations: d-glucose (Aldrich 99.5%), d-guluronic acid sodium salt (GLA, BIOSYNTH International 98%), d-glucaric acid monopotassium salt (GRA, Aldrich 98%), d-glucaric acid-1,4-lactone (Aldrich 98%), calcium 2-keto-d-gluconate (2-keto, Aldrich 99%), potassium 5-keto-d-gluconate (5-keto, Aldrich 98%), glucuronolactone (Aldrich 99%), and succinic acid (Aldrich 99%). Additionally, ^18^O_2_ (Aldrich, 99 atom % ^18^O) was used as a mass spectroscopy standard.

Catalysts composed of 1 wt % Pt on extruded carbon pellets (Pt/C, Exacer EX-C170, 1.5 mm diameter) were synthesized by contract (BC Berlin Catalysts GmbH). These pellets were crushed and sieved to retain 230–520 μm diameter particles for trickle-bed reactions or further crushed below 230 μm diameter particles for batch reactions. Catalysts were characterized both before use and following 400 h of use at relevant conditions (0–0.95 M GNA, 20–2800 kPa O_2_, 353–363 K) to determine the related changes in composition and dispersion of the nanoparticles. Platinum loadings were quantified by inductively coupled plasma optical emission spectroscopy (ICP-OES) in triplicate. Pt nanoparticle cluster diameters were quantified by transmission electron microscopy (FEI Technai F30 TEM, 300 kV); representative micrographs and the resulting cluster size distributions are provided in the Supporting Information (Supporting Information (SI) section S1). Combining TEM and ICP measurements give an estimate of the Pt site densities with the assumptions of hemispherical structures and bulk metal crystallographic properties.^29^ Triplicate CO pulse chemisorption measurements were used to confirm these site densities (Micromeritics AutoChem III, with an initial oxidation in 20% O_2_ in He at 373 K for 0.5 h, a reduction in 10% H_2_ in Ar at 423 K for 1 h, then cooling in He for CO uptakes at 313 K), taking a 1:1 CO:Pt_surf_ ratio. Table 1 summarizes the physical properties derived from these characterization techniques.

**Table 1:** Pt/C Catalyst Composition and Characterization

Pt loading by ICP (wt.% Pt)		average diameter from TEM ⟨*d*⟩ (nm)^a^			
initial	after 400 h on stream	initial	after 400 h on stream	site density from ICP and TEM (μmol Pt_surf_ g_cat_^–1^)	site density from CO chemisorption (μmol Pt_surf_ g_cat_^–1^)
0.86%	0.83%	4.3(±2) nm	6.5(±2) nm	12 ± 2	14 ± 3

a Error bars on <d> represent the standard deviations of Gaussian cluster size distributions.

### 2.2 Electrochemical Characterization of Pt/C with Cyclic Voltammetry and Stepwise Polarization

Coverages of GNA-derived species were measured with cyclic voltammograms of Pt/C working electrodes following previously reported methods.^30^ These voltammograms were obtained within a glass two-compartment H-cell (StonyLab, 200 mL) using a potentiostat workstation (BioLogic, VSP-3e) and a high loading Pt/C material (20 wt % Pt on XC-72R, Fuel Cell Store, 2–3 nm Pt clusters). Briefly, the Pt/C (10 mg) was dispersed *via* sonication into an isopropanol–water solution (2 cm^3^, 3:1 volume ratio) and pipetted dropwise into a carbon felt (Thermo Scientific, 99%, 2 × 1.5 × 0.6 cm), which was affixed to a graphite rod (1/8″ diameter) with epoxy (two-part quick set epoxy, mixed with Vulcan carbon XC-72 to retain a conductive connection) to form the working electrode. This working electrode was placed in the same compartment as a leak-free Ag/AgCl reference electrode (Innovative Instruments Inc., LF-3.2) and physically separated from a platinized titanium mesh counter electrode (Fuel Cell Store) by a proton exchange membrane (H_2_SO_4_-treated Nafion 117). Solutions of perchloric acid (0.1 M HClO_4_) were added into both compartments, and those in the working electrode compartment were continually sparged with Ar (600 cm^3^ h^–1^) with stirring at 600 rpm. Prior to the addition of gluconolactone to the working electrode compartment, a series of electrochemical pretreatments were performed. Cyclic voltammetry (30 cycles, 0.05 to 1.2 V versus the reversible hydrogen electrode (V_RHE_), 100 mV s^–1^) removed organic adsorbates from the Pt surfaces. Then, impedance spectroscopy at open circuit (10^–1^–10^5^ Hz) quantified the solution resistance (Ω_s_). Impedance spectroscopy at open circuit was collected after each addition of gluconic acid to obtain the corresponding Ω_s_ values, which ranged from 2.6 to 3.0 Ω. Hydrogen atoms were deposited then desorbed by cyclic voltammetry in the underpotentially deposited hydrogen (H_UPD_) regime from 0.08 to 0.45 V_RHE_ with a scan rate of 10 mV s^–1^ at each concentration of gluconic acid (0.01–0.5 M) while continually sparging with pure Ar (101 kPa Ar, 600 cm^3^ h^–1^) and stirring (600 rpm). The resulting coverage of H_UPD_ was quantified by integrating the charge during H_UPD_ desorption sweeps and subtracting the baseline capacitive current.

Stepwise polarization curves were obtained using the same electrodes and cell setup as for cyclic voltammetry measurements. The rates of the electrochemical O_2_ reduction (ORR), O_2_ evolution (OER), and GNA oxidation (GNA-OR) half-reactions were assessed by controlling the electrode current and measuring the corresponding electrode potentials. Each measurement proceeded until the potential remained constant within 1 mV for at least 30 s (typically less than 300 s). All polarization curves were obtained while continually sparging with pure O_2_ (101 kPa O_2_, 600 cm^3^ h^–1^) and stirring (600 rpm). The measured potentials were then manually corrected for *iR* drop (100% correction). The currents were normalized by the Pt site density (estimated from the reported weight loading and dispersion) to give the electron consumption or generation rates on the Pt/C working electrodes.

### 2.3 Steady-State and Transient Kinetics in a Trickle-Bed Reactor with Open-Circuit Potentiometry

Steady-state and transient kinetic experiments were performed in a trickle-bed reactor system that combines analytical tools to measure reactant depletion, product formation, and *E*_cat_ values, described previously.^6^ Briefly, this reactor system consists of high-pressure liquid pumps for liquid delivery (Chrom Tech, SSI LS class) and mass flow controllers for gas delivery (Parker, 601-AAASKCAA) upstream of a stainless-steel trickle-bed reactor. The solid catalytic materials (e.g., Pt/C and bare C pellets, 0.2–0.7 g, 230–520 μm) were supported by silanized glass wool (Aldrich) in a cylindrical stainless-steel reactor (1.2 cm I.D., 30 cm long). The temperature of the reactor was controlled by an aluminum heating mantle with cartridge heating elements by using an electronic temperature controller (Watlow, EZ-ZONE), and the temperature was monitored by using two K-type thermocouples (Omega, TJ36-CASS-18U-6SB-SMPW-M) submerged within the aluminum heating jacket. The pressure of the system was monitored by a digital pressure transducer (Omega, PX409-1.0KGUSBH) and controlled by a back-pressure regulator (Equilibar, LF Series) using a digital electronic pressure regulator (Equilibar, GP1). The reactor housing integrates two components: a conductive carbon cloth (Fuel Cell Store) in contact with both the catalyst bed and a PTFE-sheathed wire (Conax Technologies, MTG24T­(K)-A2-T) as the working electrode and a PEEK leak-free Ag/AgCl reference electrode (Innovative Instruments Inc., LF-3.2) located at the same axial position as the working electrode and catalyst bed. The open-circuit potential was monitored by connecting these electrodes to a potentiostat (PalmSens, EmStat 4R). The liquid and gaseous effluents from the reactor were cooled and separated; then, gas was vented, and liquid was collected using a fraction collector (Bio-Rad, 2110).

Catalysts were loaded into the fixed-bed reactor and subjected to a standardized sequence of conditions prior to obtaining measurements. To begin, the Pt/C catalyst was treated in a flowing stream of dilute hydrogen (10 kPa H_2_, 91 kPa Ar, 3000 cm^3^ h^–1^) and heated from ambient temperature to 423 K (120 K h^–1^), held for 1 h at 423 K in the flowing mixture, and then purged with pure Ar (3000 cm^3^ h^–1^) while cooling to 298 K. Then, while continuing to flow Ar, the Pt/C catalyst bed was heated at a rate of 120 K h^–1^ to 363 K (10 K above the reaction temperature), then flushed with H_2_O at 363 K for 0.1 h (600 cm_liquid_^3^ h^–1^), after which the liquid flow was changed to an aqueous solution of gluconic acid (0.1 M GNA in H_2_O, 600 cm_liquid_^3^ h^–1^) for 0.2 h. Subsequently, the GNA solution flow rate was reduced (30 cm_liquid_^3^ h^–1^), the gas flow was switched to pure O_2_ (3000 cm^3^ h^–1^), and the system was pressurized (1400 kPa O_2_). Steady-state rates, selectivities, and values of *E*_cat_ were achieved after operating at fixed conditions for 48 h. Reported values for O_2_ partial pressures account for partial pressure contributed by H_2_O (20–70 kPa H_2_O) to the total measured pressure at each temperature (333–363 K).

Reference electrodes utilized within the fixed-bed reactor were calibrated against an independent Ag/AgCl reference electrode (BASi MF-2056) in an aqueous KCl solution (3 M KCl) before and after each trickle-bed reactor experiment. Previous work provides detailed methods for reference electrode stability and their conversion to the reversible hydrogen electrode (RHE) scale.^6^ Temperature corrections were applied to measured potentials following previously established corrections for Ag/AgCl reference electrodes.^31^ Upon pressurizing the system, an equal pressure was applied to the back of the reference electrode to prevent leaks, decreasing the relative drift during the experiments. The drift in reference electrode potentials was less than 56 mV over the course of a series of experiments lasting 430 h on stream, which provides the greatest source of uncertainty in reported values of *E*_cat_. Under all conditions, gluconic acid serves as the electrolyte, and adding an additional ionic component (e.g., sodium perchlorate) minimally affects the reported *E*_cat_ values within the margin of error (SI section S2). RHE is defined as 1 bar H_2_, the measured pH, and the measured temperature under all conditions herein.

Liquid stock solutions of gluconic acid (GNA) or γ-hydroxybutyric acid (GHB) were prepared by dissolving their corresponding lactones in water, then stored in ambient air for at least 24 h (for GNA) or 96 h (for GHB) to allow the lactone hydrolysis to reach equilibrium as demonstrated by repeated compositional analysis. During experiments, these aqueous solutions were purged continually with pure argon (101 kPa Ar, 50 cm^3^ min^–1^) to remove dissolved O_2_ and CO_2_. Values of pH (in H_2_O) and pD (in D_2_O) for the stock solutions and aqueous fractions were quantified with a glass pH probe (Mettler Toledo, LE438 IP67 probe) at 293 ± 1 K.

### 2.4 Batch Kinetics and Isotope Exchanges

Batch kinetic experiments were performed within isothermal batch stirred tank reactors (Parr Instruments, 70 cm^3^) operated in parallel with independent temperature and pressure control (Parr Instruments, Multi-Parr 5000). For rate assessments, Pt/C catalyst (0.1–0.3 g) was added to the liquid reactant mixture (35 cm^3^) in a PTFE liner and sealed into each reactor along with a magnetic PTFE stir bar. The reactor headspace was then purged five times with pure argon before pressurizing the reactor (600 kPa Ar) and heating it to 353 K while stirring (600 rpm). After the temperature stabilized, the headspace was purged five times with O_2_ and set to the desired pressure (1400–1600 kPa O_2_). Shortly after, the first liquid aliquot was sampled through a filtered reactor dip tube, and the reaction clock time was defined to be zero. Subsequent aliquots were taken after purging the reactor dip tube by discarding 1 cm^3^ of liquid, and each time the headspace was repressurized with O_2_ to return to the desired pressure. Catalyst concentrations (g_cat_ cm^–3^) were corrected for each time point by subtracting the volume of liquid removed in each aliquot.

Isotope exchange experiments that utilized combinations of oxygen-18 labeled water (H_2_^18^O) and oxygen-16 labeled gas (^16^O_2_) were performed in the same batch reactor setup; however, the reaction conditions were modified (2 cm_liquid_^3^, 400 kPa O_2_). Gas headspaces were periodically sampled and stored in gas bags (Ted-lar, 600 cm^3^). The mass fragmentations corresponding to the relative isotope distribution of ^16^O_2_, ^16^O^18^O, and ^18^O_2_ in the headspace (32, 34, and 36 *m*/*z* ^+^, respectively) were quantified by a continuously sampled quadrupole mass spectrometer (Pfeiffer, OmniStar).

### 2.5 Product Derivatization and Quantification

A chemical derivatization procedure was developed utilizing silylation chemistry with BSTFA as the silylation agent to quantify the composition of liquid aliquots obtained from GNA oxidation experiments, as described previously.^6^ Briefly, aqueous aliquots (20 μL) were combined with an internal standard solution (0.01 M 1,3-butanediol in DMF, 500 μL) and BSFTA (600 μL), which corresponded to a 100% molar excess of the silylating reagent relative to the total number of hydroxyl groups in the liquid aliquots (water and organic species). These mixtures were then heated to 338 K and held for 1 h before analysis by a gas chromatograph (Agilent, 8890 GC) equipped with a capillary column (Zebron ZB-5 60 m × 0.32 mm × 0.25 μm) and a flame ionization detector. This full procedure was also used to obtain the calibration curves for all products. A representative gas chromatograph at 20% GNA conversion is provided in Supporting Information (SI section S3). A simpler procedure was sufficient to quantify the composition of the reactor effluent during γ-hydroxybutyric acid (GHB) oxidation. The aqueous effluent (300 μL) was combined with an aqueous internal standard solution (0.2 M C_2_H_5_OH, 300 μL) and quantified directly using GC. These methods yielded carbon molar balances that were within 10% of unity for all experiments (0–20% GNA conversion).

Products generated by oxidation reactions of GNA within H_2_^18^O–^16^O_2_ environments were quantified on a liquid chromatograph (Agilent, 1260 Infinity II) equipped with a reverse-phase column (Poroshell, Model 120 EC-C18) and a quadrupole mass spectrometer (Agilent, 6120). The separations were conducted using a mobile phase of 5 vol% acetonitrile and 0.1 vol% formic acid in water.

### 2.6 Calculation of GNA Oxidation Turnover Rates

The total consumption rates for oxidative conversion of GNA (−*r̂*_GNA_, where the circumflex accent denotes rate units of mol (g_cat_ s)^−1^) correspond to the sum of formation rates (*r̂*_*j*_) for four major products *j*: guluronic acid (GLA), glucaric acid (GRA), 2-keto-gluconic acid (2-keto), and 5-keto-gluconic acid (5-keto). This sum, normalized by the Pt site density ([*L*], mol-Pt_surf_ g_cat_^–1^, provided in Table 1), gives the GNA consumption turnover rates (−r_GNA_, in units of (mol GNA) (mol Pt_surf_ s)^−1^):

$$-r_{\mathrm{GNA}}=\frac{-{\mathrm{r̂}}_{\mathrm{GLA}}}{\left[L\right]}=\left(\frac{1}{\left[L\right]}\right)\times ({\mathrm{r̂}}_{\mathrm{GLA}}+{\mathrm{r̂}}_{\mathrm{GRA}}+{\mathrm{r̂}}_{\mathrm{2‐keto}}+{\mathrm{r̂}}_{\mathrm{5‐keto}})$$ 5

## 3 Results and Discussion

### 3.1 GNA-O_2_ Reaction Network and Oxidation Rates on Pt/C Catalysts

Figure 1a portrays the network of reactions responsible for aqueous aerobic glucose oxidations consistent with observations described in previous studies.^32–34^ Glucose is first oxidized to gluconic acid (GNA). GNA contains five hydroxyl groups that may oxidize to primary products (guluronic acid as well as 2-, 3-, 4-, and 5-keto-gluconic acid products) and subsequently react further in sequential partial oxidation or decarboxylation reactions. In parallel, GNA molecules interconvert with γ- and δ-gluconolactone and water through a reversible and likely quasi-equilibrated reaction. During reactions among GNA and O_2_ on Pt/C, four products comprise greater than 95% of the carbon selectivity when the GNA conversion remains below 10%. These products include guluronic acid (GLA), glucaric acid (GRA), 2-keto-gluconic acid (2-keto), and 5-keto-gluconic acid (5-keto). This reaction network demonstrates that the rate of GNA oxidation by a primary oxidation of a hydroxyl group (*−r*_GNA_, eq 5) reflects the limiting process for the production of the desired GLA and GRA products from glucose.

**Figure 1:**
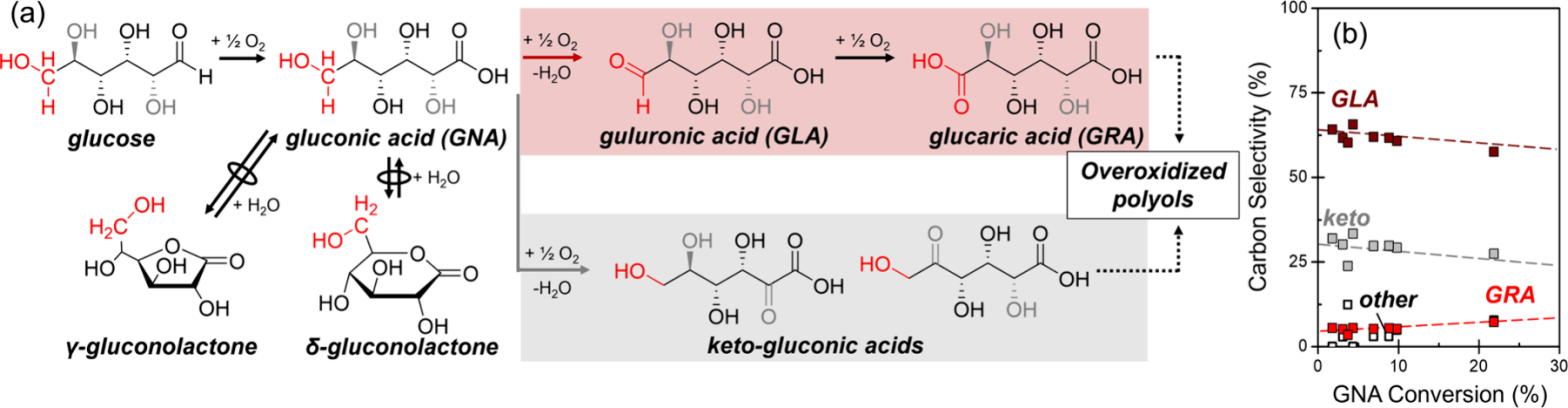
(a) Simplified glucose and gluconic acid (GNA) partial oxidation reaction network. (b) Carbon selectivities to guluronic acid (GLA, brown), glucaric acid (GRA, red), keto-gluconic acids (keto, gray), and sequential oxidation products (other, white) as a function of GNA conversion (0.12 M GNA (6–90 cm_liquid_^3^ h^–1^), 1400 kPa O_2_, 353 K).

Figure 1b shows that the steady-state carbon selectivities of GLA and keto products decrease and those of GRA and overoxidized polyols increase as the conversion of GNA increases (0–25% GNA conversion). These steady-state observations agree with the analogous conversion-selectivity profiles of GNA oxidation conducted in batch stirred tank reactors under identical conditions (SI section S4). Selectivities to both GLA and keto-gluconic acids possess finite values when extrapolated to zero conversion, which confirm that two parallel oxidation pathways consume GNA. Selectivities to GLA range from 60 to 70%, which indicate Pt nanoparticles preferentially oxidize the terminal alcohol of GNA, well documented under basic conditions as the Heyns oxidation reaction.^4,34,35^

The GNA consumption rates reported here reflect intrinsic reaction kinetics on the surfaces of Pt nanoparticles, as demonstrated by the following control experiments. First, reactions among aqueous GNA and O_2_ occur exclusively on the surface of Pt nanoparticles, because specific GNA oxidation rates (−*r̂*_GNA_) measured on Pt/C (2 × 10^–7^ mol (g_cat_ s)^−1^) exceed those for the bare carbon support (<2 × 10^–10^ mol (g_cat_ s)^−1^) by more than 3 orders of magnitude (0.12 M GNA, 1400 kPa O_2_, 353 K). Electrochemical decoupling of O_2_ reduction and GNA oxidation half-reactions reveals that both half-reactions occur predominantly on Pt domains, and not the bare carbon support (SI section S5). Second, near-steady-state values of *–r*_GNA_ in the fixed-bed reactor decrease as a function of time with two distinct deactivation regimes (representative time on stream profiles provided in SI section S6). Initially, rates decay exponentially and *–r*_GNA_ decreases by approximately 30% over 48 h (0.12 M GNA, 1400 kPa O_2_, 363 K), which signifies an induction period responsible for establishing steady-state coverages of reactive intermediates. After this induction period, rates decay linearly with time, and values of *–r*_GNA_ decrease by approximately 30% over 270 h. Rates were corrected for this second form of deactivation by linear interpolation among multiple measurements acquired at a standardized set of reaction conditions (0.12 M GNA, 1400 kPa O_2_, 353 K), following the instantaneous rate ratio methodology.^36^ In both deactivation regimes, we ascribe these rate decreases to the accumulation of strongly adsorbed, unreactive spectator surface intermediates.^4^ Third and finally, the measured GNA oxidation rates represent intrinsic kinetics of the reaction, because these rates remain constant with differences in Pt loading on carbon (0.5–3 wt % Pt/C)^6^ and among different liquid hourly space velocities (SI section S7, GNA conversion between 0 and 10%). These observations demonstrate that internal and external mass transport processes do not influence the observed reaction rates.^37^

### 3.2 Mechanism of GNA Oxidation Reactions

Rates measured as functions of reactant concentrations and the apparent effects of isotope substitution give evidence of the mechanism of GNA oxidation on Pt surfaces. Figure 2 shows that GNA oxidation rates increase as sublinear functions across a wide range for the O_2_ partial pressures (Figure 2a, *–r*_GNA_ ∼ [O_2_]^0.0–0.2^) and GNA concentrations (Figure 2b, *–r*_GNA_ ∼ [GNA]^0.0–0.1^). The weak dependence of rates on these reactants suggests that reactive intermediates derived from O_2_ and GNA reside at high coverages on the active sites of the Pt nanoparticles. Cyclic voltammograms and quantities of underpotential-deposited hydrogen atoms (H_UPD_) measured across a similar range of [GNA] values provide a quantitative measure for the fractional coverage of organic intermediates.^30^ These analyses reveal an apparent adsorption equilibrium constant of GNA-derived intermediates on the order of 100 M^–1^ at 293 K and indicates that more than 50% of Pt surface sites bind GNA-derived species even at the lowest concentrations examined (SI section S8, 0.01–0.5 M GNA, 0.1 M HClO_4_, 293 K). Critically, the Pt site coverages by GNA provide additional evidence that GNA oxidation half-reactions occur through a surface-mediated pathway, as opposed to a noncompetitive adsorption onto the bare carbon support. These observations suggest that rates involve kinetically relevant reactions among GNA- and O_2_-derived surface intermediates that exist at high coverages across the range of relevant conditions.

**Figure 2:**
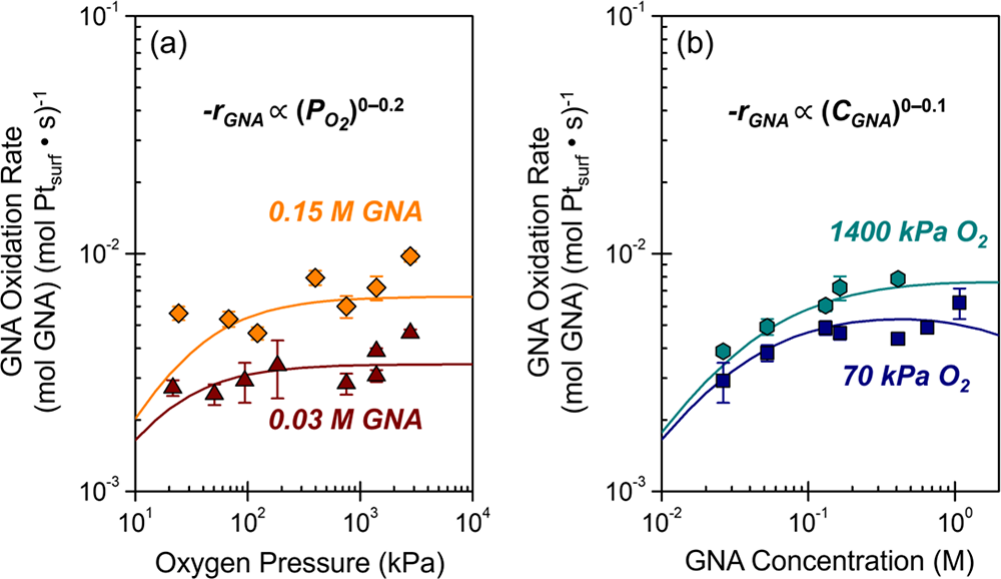
Turnover rates for oxidation of GNA as functions of (a) O_2_ pressure and (b) GNA concentration over Pt nanoparticles (0.025–0.95 M GNA, 20–2800 kPa O_2_, 353 K). Trendlines represent regressed fits to the rate expression described in eq 7.

Ratios of the rates for consumption of the reagents and the corresponding deuterated isotopologues yield values for the H-D kinetic isotope effects (*r*_H_/*r*_D_). Figure 3a–c compares rates for oxidation of γ-hydroxybutyric acid (GHB) and selectively deuterated forms of GHB that possess either deuterium-substitution only of C–H bonds (green) or only of O–H bonds (blue) (SI section S9 presents analysis of concentration–time profiles obtained from batch reactors). The molecule GHB serves as an informative surrogate for GNA in measurements of kinetic isotope effects, because both GHB and GNA possess terminal hydroxyl and carboxylic acid groups and interconvert with their lactone forms (γ-butyrolactone and gluconolactone, respectively). These comparisons give values of *r*_C–H_/*r*_C–D_ greater than two and suggest that GHB oxidation rates involve kinetically relevant C–H bond scission, which aligns with findings for aqueous ethanol oxidation on Pt.^38^

**Figure 3:**
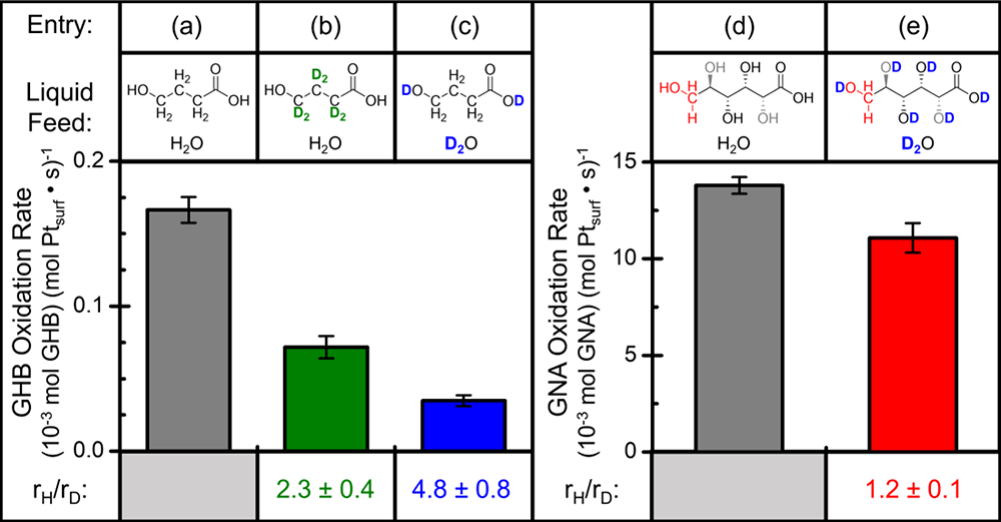
Differences in rates of oxidation of γ-hydroxybutyric acid (GHB) or gluconic acid (GNA) in aqueous solutions in response to changes in isotope substitution that include (a) GHB/H_2_O, (b) D_6_-GHB/H_2_O, and (c) GHB/D_2_O and during steady-state GNA oxidation reactions with (d) GNA/H_2_O and (e) GNA/D_2_O (0.10 M GHB or 0.12 M GNA, 1400–1600 kPa O_2_, 353 K).

Oxidation of GHB proceeds at much lower rates in D_2_O as opposed to H_2_O (*r*_H_2_O_/*r*_D_2_O_ = 4.8), and this observation appears inconsistent with reports for ethanol oxidation.^38^ In contrast, GNA oxidation rates decrease minimally with D_2_O feeds (*r*_H_2_O_/*r*_D_2_O_ of 1.2, Figure 3d,e), which suggests that the large *r*_H_2_O_/*r*_D_2_O_ values remain unique to GHB oxidation and do not reflect kinetically relevant O–H bond scission. The discrepancy between GNA and GHB may arise from D_2_O shifting lactone-acid equilibria toward the lactone, where the capped hydroxyl groups in lactones prevent alcohol oxidation. On the other hand, neither γ- or δ-gluconolactone formation caps the terminal hydroxyl group of GNA. pH (3.7 ± 0.1) and pD (5.6 ± 0.2) measurements of 0.1 M GHB stock solutions indicate D_2_O solvents decrease the concentration of carboxylate anions in the solution 100-fold, which agrees with a shift in lactone-acid equilibria toward the lactone. Values of *r*_C–H_/*r*_C__–D_ for GHB oxidation inform understanding of GNA oxidation, because these rate ratios reflect the ratio of rate constants (*k*_C–H_/*k*_C__–D_) since carboxylate anion concentrations remain equal. In contrast, *r*_H_2_O_/*r*_D_2_O_ reports on differences in ratios of rate constants as well as differences in concentrations of ring-open carboxylate anions induced by differences in pH and pD values.

Guided by the kinetic and isotopic results, Scheme 1 provides a plausible catalytic cycle for reaction among GNA- and O_2_-derived intermediates on Pt nanoparticles. The catalytic cycle contains kinetically coupled half-reactions that reduce O_2_ (blue) and oxidize GNA (red). The reduction of O_2_ occurs through a sequence of elementary steps consistent with mechanisms proposed for equivalent electrochemical reactions.^21,39,40^ Initially, O_2_ adsorbs to unoccupied sites (*) on the Pt surface (Step O.1) to form OO* moieties, which subsequently form an adduct with H_2_O upon partial negative polarization (OH_2_···OO*, Step O.2). Then, this adduct reacts to form adsorbed hydroperoxyl (HOO*) and hydroxyl (HO*) moieties (Step O.3). Sequential O–O bond scission and O–H bond scission and formation steps give three equivalents of HO* (Steps O.4 and O.5) and completes the half-reaction. The surface HO* species react with GNA in the oxidation half-reactions, which resemble the aqueous-phase oxidation of alcohols on Pt^38^ as well as vapor-phase oxidation of primary alcohols on noble metals.^41^ To begin, GNA adsorbs on an unoccupied Pt-active site to form adsorbed GNA moieties (RCH_2_O­(H)*, R = C_5_H_9_O_6_) (Step G.1). Next, adjacent HO* species abstract a H-atom from RCH_2_O­(H)* *via* a kinetically relevant C–H bond scission step (Step G.2) to form an adsorbed hydroxy-guluronic acid intermediate. Irreversible and kinetically inconsequential O–H bond scission (Step G.3) then quasi-equilibrated desorption of GLA (Step G.4) completes the GNA oxidation half-reaction and the full catalytic cycle. Keto-gluconic acids form through an analogous sequence of elementary steps that cleave internal C–H bonds (rather than terminal C–H bonds), and GRA forms by the oxidation of GLA following aldehyde oxidation half-reactions analogous to glucose that cleave the aldehyde C–H bonds.^6^

**Scheme 1:**
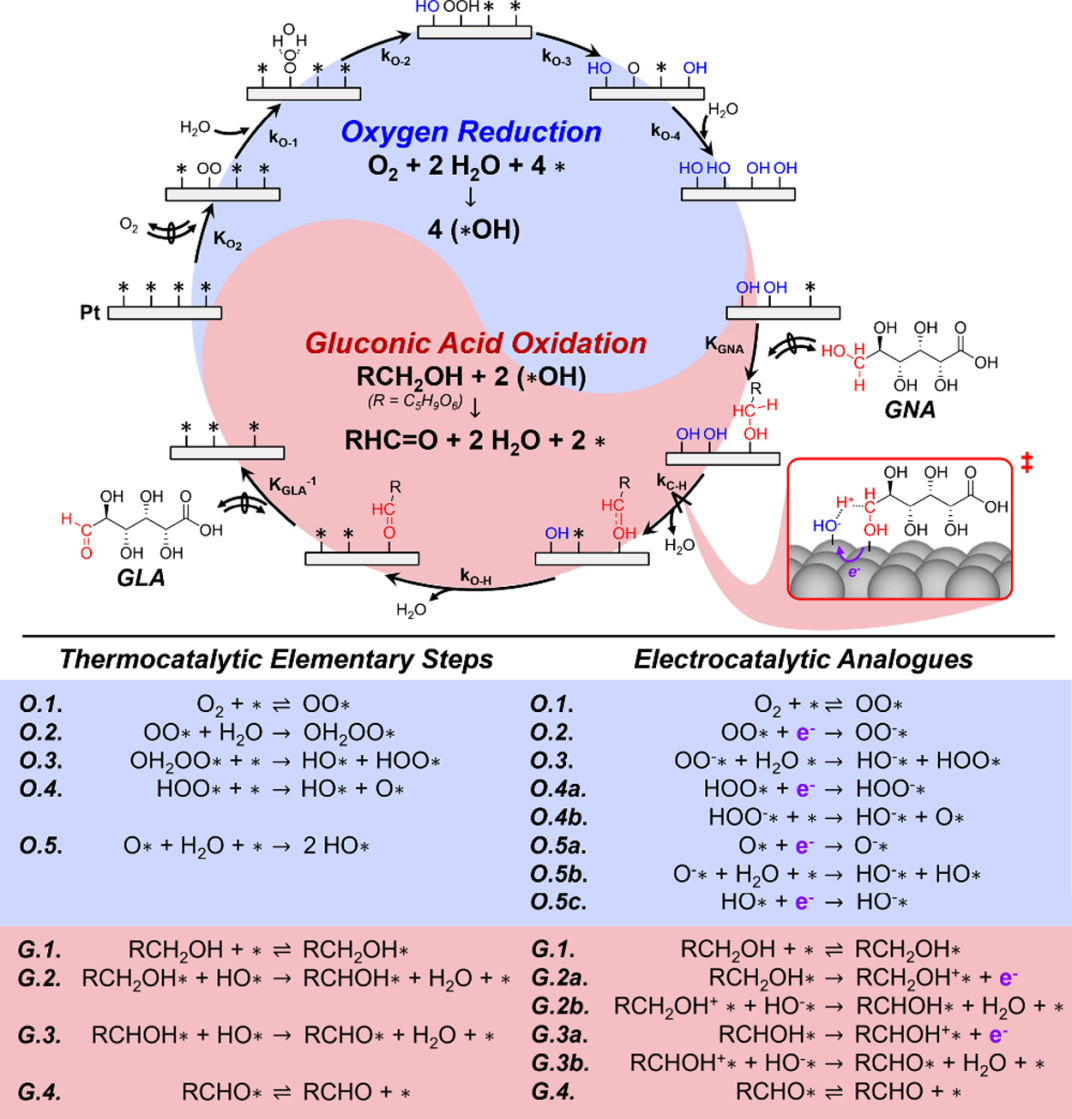
Proposed Sequence of Elementary Steps for Reactions among GNA and O_2_ on Pt Nanoparticles

The O_2_ reduction half-reaction may proceed reversibly or irreversibly during steady-state catalysis, and each case yields a different expression for HO* coverages. Analysis of the distribution of O_2_ isotopologues (^16^O_2_, ^16^O^18^O, and ^18^O_2_) produced during reactions between GNA and ^16^O_2_ in H_2_^18^O offer one method to probe the reversibility of the O_2_ reduction process (0.1 M GNA, 400 kPa O_2_, 353 K; SI section S10 describes the method, interpretation, and consistency with electrochemical assessments of O_2_ reduction and O_2_ evolution). Neither ^16^O^18^O or ^18^O_2_ formed during reactions in the presence of GNA when compared on the same reaction time scale, which indicates that the reverse rate of O_2_ reduction proceeds slower than the GNA consumption rate (4 × 10^–3^ (mol GNA) (mol Pt_surf_ s)^−1^). Notably, multiple ^18^O atoms from the H_2_^18^O solvent exchange with ^16^O of GNA (2 ^18^O) and with oxidation products (2–4 ^18^O), and the number exchanged correlates as twice the number of carboxylic acid groups (SI section S11). These observations suggest that the oxygen atoms in carboxylic acids completely scramble in acid *via* reversible lactone formation and hydrolysis. Critically, the extent of ^18^O incorporation differs from glycerol oxidation on Au/C in base,^9^ during which the oxygen atoms in hydroxyl groups scramble with ^18^O from H_2_^18^O due to glyceraldehyde-ketone interconversion.

Steady-state rates of GNA consumption equal the rates for irreversible C–H bond activation (Scheme 1, Step G.2) across all conditions, which allows these rates to be restated in the form of rate constants (*k*_*i*_) and the surface densities of intermediates ([j*]) involved:

$$\frac{-r_{\mathrm{GNA}}}{\left[L\right]}=\frac{r_{G.2}}{\left[L\right]}=k_{C-H}\left(\frac{\left[{\mathrm{RCH}}_{2}{\mathrm{OH}}^{*}\right]}{\left[L\right]}\right)\left(\frac{\left[{\mathrm{HO}}^{*}\right]}{\left[L\right]}\right)$$ 6

where [*L*] denotes the number of active sites. Application of the pseudo-steady-state hypothesis to the reactive intermediates generated and consumed (Scheme 1) gives a turnover rate expression consistent with apparent rate orders for both O_2_ and GNA, observed kinetic isotope effects, and the irreversibility of O_2_ reduction (derivation in SI section S12):

$$\frac{-r_{\mathrm{GNA}}}{\left[L\right]}=\frac{2k_{O.1}K_{O_{2}}P_{O_{2}}}{1+\left(\frac{2k_{O.1}K_{O_{2}}P_{O_{2}}}{k_{C-H}K_{\mathrm{GNA}}C_{\mathrm{GNA}}}\right)+K_{O_{2}}P_{O_{2}}+K_{\mathrm{GNA}}C_{\mathrm{GNA}}}$$ 7

where the four terms within the denominator represent the numbers of unoccupied sites/H_2_O (i.e., [*], or [H_2_O*]), hydroxyl groups ([HO*]), diatomic oxygen ([OO*]), and GNA ([RCH_2_O­(H)*]) covered sites, respectively. Linear regression of the kinetic dependencies in Figure 2 against eq 7 gives a mean regression error of 14% (regressed constants and parity plots provided in SI section S13). Analysis of six alternative mechanisms led to distinct rate expressions, distinguished by the sequence or reversibility of elementary steps and assumptions regarding site competition among oxygen reduction and GNA oxidation half-reactions. The derivations and comparisons among all mechanisms examined appear in SI sections S12 and S13. The mechanism illustrated in Scheme 1 (and corresponding rate expression, eq 7) remains the most consistent with all experimental observations while retaining a low mean regression error. Taken together, we conclude that the aerobic oxidation of GNA proceeds *via* irreversible GNA and O_2_ activation on the same Pt-active site.

### 3.3 *In Situ* Open-Circuit Potentiometry Connects Pt Nanoparticle Polarization to Reactant Ratios

Scheme 1 captures a single catalytic cycle that kinetically couples two thermochemical half-reactions *via* the coverages of HO*. Any given homolytic elementary step in this scheme that involves charge transfer between a molecule and a surface-bound intermediate (i.e., C–H scission and H–O formation) may proceed heterolytically through an analogous sequence of electrochemical processes. Rates of electron transfer into and out of the Pt nanoparticles determine the catalyst electrode potential, and these rates depend on both rates of GNA oxidation and the rates of the O_2_ reduction half-reactions. Knowledge of the relationships between the electrode potential and these processes requires a reliable measure of the open-circuit potential (OCP) during steady-state catalysis and verification that the experimental observation reflects the electrode potential (*E*_cat_) of the Pt nanoparticles that mediate catalysis.

Figure 4 shows that the OCP values measured during steady state reactions among GNA and O_2_ span a range greater than 400 mV and consistently appear at less positive (i.e., more reducing potentials) than those observed in the presence of only O_2_ and water. In the absence of GNA, OCP values increase from 1200 to 1250 mV_RHE_ with a logarithmic dependence on [O_2_] (approximately 12 mV increase per 10-fold increase in [O_2_], 20–2800 kPa O_2_, *a*_H_2_O_ = 1, pH 6.6–7.3, 353 K; Figure 4a), which aligns closely with the predicted values of the equilibrium electrochemical potential for the O_2_ reduction reaction described by the Nernst equation.^42^ These measured OCP values show that the Pt nanoparticles achieve *E*_cat_ values representative of the equilibrium potential for coupled O_2_ reduction and O_2_ evolution half-reactions that proceed at rates of equal magnitude when no other redox half-reactions occur (i.e., in the absence of organic reductants). The attainment of these equilibria requires a sufficiently long residence time of O_2_ and water within the fixed-bed reactor to allow O_2_ evolution rates to exceed rates of other electrochemical oxidation half-reactions (e.g., H_2_O oxidation to H_2_O_2_) that contribute to the net current transferred through Pt nanoparticles (*i*_net_, following eq 4), as seen by the decrease in *E*_cat_ with increasing flow rate at 100 kPa O_2_ (Figure 4a, hollow points). In contrast, the OCP value obtained from bare carbon supports (i.e., without Pt nanoparticles) does not depend on [O_2_], likely because the O_2_ molecules do not activate on carbon and only GNA deprotonation and adsorption occur. As a result, values of *E*_cat_ on carbon remain near 200 mV_RHE_ at relevant reaction conditions (0.12 M GNA, 1400 kPa O_2_, pH 2.5, 363 K). These findings demonstrate that the OCP measurements of Pt/C catalysts operating at steady state reflect the value of *E*_cat_ determined by processes that occur on Pt nanoparticles.

**Figure 4:**
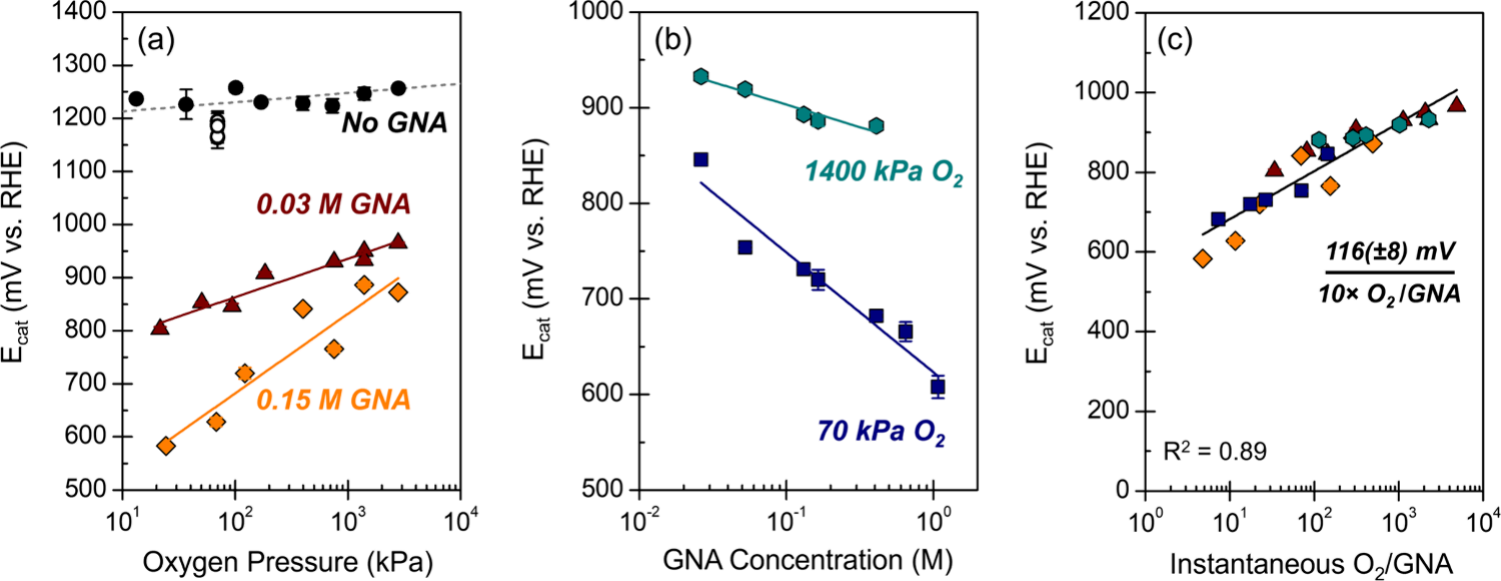
Values of the catalyst electrode potential at open circuit (*E*_cat_) as functions of (a) O_2_ pressure and (b) GNA concentration during steady-state reactions of O_2_ and GNA (0.025–0.95 M GNA, 20–2800 kPa O_2_, pH 2–3 with GNA or 6.6–7.3 without GNA (●: 40 cm_H_2_O_^3^ (g_cat_ h)^−1^, and ○: 150–450 cm_H_2_O_^3^ (g_cat_ h)^−1^), 353 K). Included in (a) is the equilibrium potential for O_2_ reduction (gray dashed line). (c) Values of *E*_cat_ as a function of the instantaneous molar ratio of O_2_ to GNA.

Values of *E*_cat_ increase logarithmically with [O_2_] (20–2800 kPa) for any fixed value of [GNA] (Figure 4a) and decrease as a logarithmic function of [GNA] (0.025–0.95 M) at each set [O_2_] (Figure 4b) over Pt nanoparticles. Reactions among GNA and O_2_-derived species cause values of *E*_cat_ to remain between those measured for reactions of only O_2_ and H_2_O (11–2800 kPa O_2_, 1200–1250 mV_RHE_, Figure 4a) and those for only GNA and H_2_O (0.1 M GNA, 101 kPa Ar, 200 mV_RHE_, *vide infra*, section 3.5), which demonstrates that *E*_cat_ represents a mixed potential determined by elementary steps from both half-reactions. A single correlation captures *E*_cat_ values obtained from all combinations of [O_2_] and [GNA] when expressed as the instantaneous molar ratio of the O_2_-to-GNA ([O_2_]/[GNA]) at the catalyst bed (Figure 4c). The form of this correlation appears as

$$E_{\mathrm{cat}}\propto (1.7\pm 0.3)\frac{RT}{F}\;\ln\left(\frac{\left[O_{2}\right]}{\left[\mathrm{GNA}\right]}\right)$$ 8

which demonstrates that values of *E*_cat_ increase by approximately 116 mV for every 10-fold increase in O_2_/GNA over 3 orders of magnitude at 353 K. The existence of this consistent relationship between *E*_cat_ and the molar ratios of the initial oxidant and reductant for this reaction network, despite the numerous intervening elementary steps and surface intermediates, suggests that the catalyst operates within a single kinetic regime (defined by a combination of kinetically relevant steps and dominant coverages) across the full range of conditions examined. These observations stand in contrast to the behavior of Pt catalysts during reactions between O_2_ and glucose: the pairs of elementary steps that determine *E*_cat_ vary significantly as glucose concentrations increase as a consequence of differences in surface coverages (0.05–1 M glucose, 20–2200 kPa O_2_, 353 K).^6^ These findings for GNA oxidation appear more similar to the observations for reactions among H_2_ and O_2_ in water (20–400 kPa H_2_, 60 kPa O_2_, 298 K), where values of *E*_cat_ increase by 60–120 mV for every 10-fold increase in the O_2_-to-H_2_ molar ratio, with slopes depending on the metal identity *via* different H_2_ oxidation and O_2_ reduction transfer coefficients.^21^ In these cases where a single set of potential-determining elementary steps gives these unified scaling relations, the oxidant-to-reductant ratio correlates to the coverages of one (or more) polarized surface intermediate (either positively or negatively charged) in order to affect *E*_cat_.

The origins of the relationships among values of *E*_cat_, the concentrations of reactants (i.e., [GNA], [O_2_]), and catalytic turnover rates appear more clearly after recasting the sequence of elementary steps for this reaction (Scheme 1) with explicit notation for electron transfer events and polarization of surface intermediates. For example, reactive HO* surface species possess negative polarization (denoted as HO^–^*) and the reactive RCH_2_O­(H)* surface species appear positively polarized (denoted as RCH_2_O­(H)^+^*) in order to produce [*^–^O­(H)···H^+^···C­(H)­(R)–O­(H)*]^⧧^ transition states for heterolytic C–H bond scission in the kinetically relevant step. Irreversible Butler–Volmer expressions for the rates of these elementary steps provide an analytical connection between *E*_cat_ and steady-state turnover rate expressions derived from otherwise identical assumptions that describe the time-independent coverages of reactive intermediates and the relative coverages of these surface species across the range of reaction conditions (*vide supra*, Section 3.2). Following Scheme 1, the net current derived from catalysis (*i*_net_) reflect contributions from all elementary steps that involve electron transfer (i.e., we do not apply any assumptions for the pair of elementary steps that determine *E*_cat_). The application of the pseudo-steady-state hypothesis to surface intermediates gives an analytical expression for *E*_cat_ that holds when the value of *i*_net_ remains equal to zero (i.e., the system operates at open circuit, complete derivation in SI section S14):

$$E_{\mathrm{cat}}=\left[\frac{RT}{(\beta_{C-H}+\beta_{O-1})F}\right]\;\ln\left(\frac{4k_{O-1}^{0\prime }K_{O_{2}}}{2k_{C-H}^{0\prime }K_{\mathrm{GNA}}}\right)+\left[\frac{RT}{(\beta_{C-H}+\beta_{O-1})F}\right]\;\ln\left(\frac{a_{O_{2}}}{a_{\mathrm{GNA}}}\right)$$ 9

where β_O–1_ and β_C–H_ represent the transfer coefficients for O_2_ reduction to O_2_^–^* (Step O.2) and GNA oxidation to RCH_2_O­(H)^+^* (Step G.2a), the rate and equilibrium constants correspond to the elementary processes shown in Scheme 1, the superscripts for rate constants *k*_O–1_^0′^ and *k*_C–H_^0′^ signify these terms contain contributions from the equilibrium potentials and transfer coefficients of their respective elementary steps, and *a*_O_2__ and *a*_GNA_ correspond to the thermodynamic activities of O_2_ and GNA, respectively.

Comparisons between the form of eqs 8 and 9 with the apparent slope between *E*_cat_ and O_2_/GNA (116 ± 8 mV for every 10-fold increase in O_2_/GNA) give the sum of transfer coefficients (β_O__–1_ + β_C–H_) equaling 0.6 ± 0.1. This sum indicates that the value of each individual elementary transfer coefficient (β_O__–1_ and β_C–H_) remains less than one-half, which may represent unusually asymmetric electron transfers^43,44^ or incomplete charge transfer and electrosorption valencies less than unity for steps that activate O_2_ and GNA reagents (steps up to O.2 and G.2a in each half-reaction). Low transfer coefficients have been reported on Pt electrodes for O_2_ reduction (β_O__–1_ = 0.3)^21^ and alcohol oxidation (β_C–H_ for methanol, ethanol, and 1-propanol ranging from 0.12 to 0.8),^45–47^ which vary widely with temperature and solution composition but agree qualitatively with our findings. The intercept of Figure 4c would give the O_2_ activation to C–H activation rate constant ratio, but these values cannot be deconvoluted from the unknown equilibrium constants and standard potentials of their elementary steps, as well as the activity coefficients of O_2_ and GNA. The constant transfer coefficients over 3 orders of magnitude in O_2_/GNA reflect a single set of elementary potential-determining kinetic processes where negative polarization of HO* and positive polarization of RCH_2_O­(H)* dictate the rates of electron transfer into and out of the Pt nanoparticles.

### 3.4 Coupling *E*_cat_ and GNA Oxidation Rates Deconvolutes Kinetic Parameters for O_2_ Reduction and GNA Oxidation Half-Reactions

The microkinetic analysis that gives eq 9 (SI section S14) also describes the turnover rate for GNA consumption (*−r*_GNA_) as a function of *E*_cat_:

$$\frac{-r_{\mathrm{GNA}}}{\left[L\right]}=\frac{k_{C-H}^{0\prime }K_{\mathrm{GNA}}a_{\mathrm{GNA}}e^{\beta_{C-H}FE_{\mathrm{cat}}/RT}}{1+K_{\mathrm{Pt}}^{0}e^{\left(FE_{\mathrm{cat}}/RT\right)}+K_{O_{2}}a_{O_{2}}+K_{\mathrm{GNA}}a_{\mathrm{GNA}}}$$ 10

In eq 10, *K*_Pt_^0^ represents the potential-dependent equilibrium (Nernstian) distribution of polarized-to-neutral Pt surface sites, which thermodynamically relates to the distribution between HO^–^* and HO* surface moieties (see SI section S14) as a description for nanoparticle polarization. As *E*_cat_ increases, the coverages of reactive HO^–^* moieties increase monotonically and eventually saturate Pt surfaces, which concurrently decreases the numbers of unoccupied sites required to bind GNA prior to kinetically relevant C–H bond activation. At the highest *E*_cat_ values (exceeding 1000 mV_RHE_), rates appear insensitive to potential because HO^–^* moieties saturate the surface and exist as the most abundant surface intermediate. These descriptions and the form of eq 10 implies that *E*_cat_ reports on the relative coverage of reactive O_2_- and GNA-derived hydroxyl surface species.

Combining eqs 9 and 10 recovers an expression more directly comparable to the thermocatalytic rate expression (eq 7):

$$\frac{-r_{\mathrm{GNA}}}{\left[L\right]}=\frac{2k_{O-1}^{0\prime }K_{O_{2}}a_{O_{2}}e^{\left(\beta_{C-H}/\left(\beta_{C-H}+\beta_{O-1}\right)\right)}}{1+\left(\frac{2k_{O-1}^{0\prime }K_{O_{2}}K_{\mathrm{Pt}}^{0}}{k_{C-H}^{0\prime }K_{\mathrm{GNA}}}e^{\left(1/\left(\beta_{C-H}+\beta_{O-1}\right)\right)}\right)\left(\frac{a_{O_{2}}}{a_{\mathrm{GNA}}}\right)+K_{O_{2}}a_{O_{2}}+K_{\mathrm{GNA}}a_{\mathrm{GNA}}}$$ 11

Comparing eq 11 to eq 7, the terms carry identical meanings; however, the thermocatalytic rate constants *k*_O__–1_ and *k*_C–H_ contain additional embedded contributions from their equilibrium potentials (superscript ′) and transfer coefficients (β_O__–1_ and β_C–H_). This commonality confirms that explicitly denoting electron transfer in the elementary steps of Scheme 1 does not affect the GNA oxidation mechanism nor the kinetic interpretations of these elementary steps made *a priori* (Section 3.2).

Figure 5 shows values for GNA oxidation rates (*−r*_GNA_) as functions of measured values of *E*_cat_ (600–1000 mV_RHE_) across the combinations of O_2_ pressures and GNA concentrations examined (Figures 2 and 4), which illustrates the relationships anticipated by eq 10. Notably, rates depend on *E*_cat_ *via* the dependence of *E*_cat_ on the coverages of HO^–^*. Consequently, a single *E*_cat_ value reflects multiple values of *–r*_GNA_ (taking a vertical line on Figure 5) when both O_2_ and GNA activities change simultaneously but at a constant molar ratio. This interpretation contrasts with prior explanations focused only upon changes in activation free energies with the catalyst potential and the impact on rates of individual half-reactions following Butler–Volmer kinetics. When RCH_2_O­(H)* or OO* coverages remain constant (approximated by fixing the GNA concentration and O_2_ pressure, solid and dashed lines of Figure 5), rates depend on *E*_cat_ with an exponential correlation that resembles a Butler–Volmer kinetic expression. In contrast to a Butler–Volmer kinetic model, *–r*_GNA_ depends nonmonotonically on *E*_cat_ (variable slopes in Figure 5), because *E*_cat_ primarily influences HO^–^* coverages but not the OO* and RCH_2_O­(H)* coverages. These slopes simply reflect the connection between HO^–^* coverages and *E*_cat_ from eq 10. In electrocatalysis, *E*_cat_ serves as an independent driving force, directly connected but decoupled from the surface coverages and the rates (in terms of their partial current densities) in both the alcohol oxidation^46,48–50^ and O_2_ reduction^42,51^ half-reactions. In contrast, *E*_cat_ serves as an *operando* probe for the coverage of polarized surface intermediates during examination of thermocatalytic (open circuit) reactions such as those reported herein. This *operando* probe offers insight that is inaccessible to other laboratory methods.

**Figure 5:**
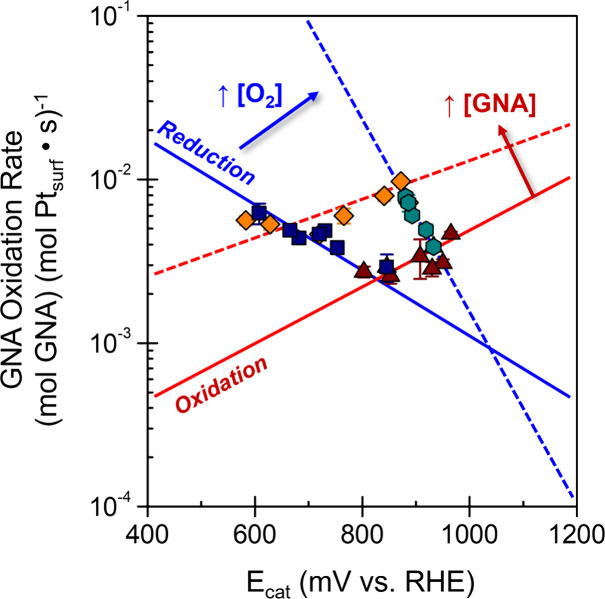
Correlations between catalyst open-circuit potential (*E*_cat_, from Figure 4) and the GNA oxidation turnover rate (Figure 2) at varying O_2_ pressures and GNA concentrations (0.025–0.95 M GNA, 20–2800 kPa O_2_, 353 K). Solid and dashed lines are provided to guide the eye.

The polarized HO^–^* surface intermediate kinetically couples O_2_ reduction and GNA oxidation half-reactions on the free-energy landscape (Scheme 2). The populations of OO*, HO^–^*, and H_2_O* surface intermediates (the initial states for C–H scission) vary sensitively with operating conditions. Conventional thermocatalytic methods to decouple the activation enthalpies and entropies of the two half-reactions involve linear regression of eq 11 against GNA and O_2_ kinetic dependencies at each temperature. Simultaneous *E*_cat_ assessments which report on HO^–^* coverages contain embedded information on the activation free energies of both half-reactions, independent of the total coverage. Thus, coupling rate assessments with *E*_cat_ at varying temperatures enables direct quantification of activation enthalpies and entropies of both O_2_ reduction and GNA oxidation (*vide infra*).

**Scheme 2:**
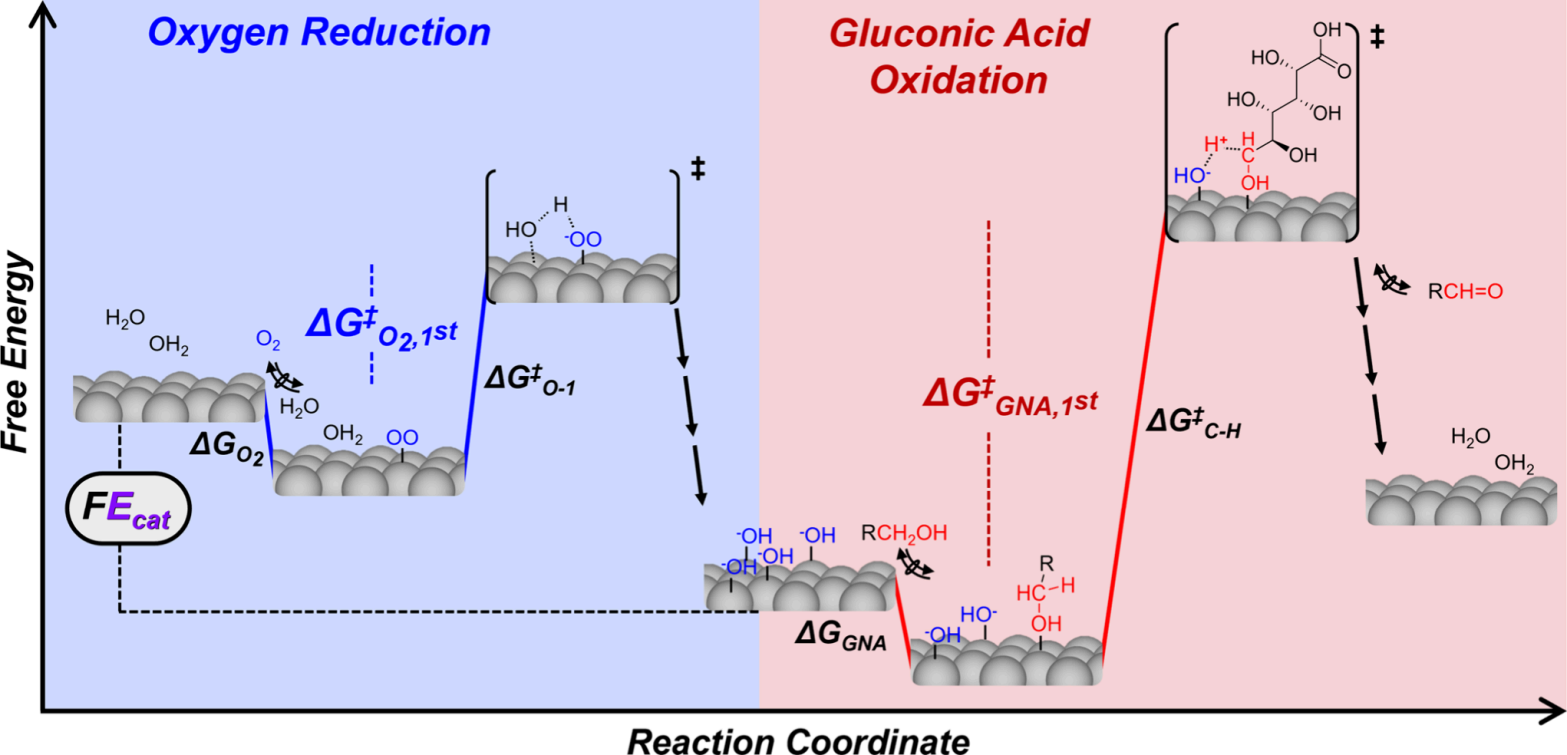
Reaction Coordinate of Oxygen Reduction and GNA Oxidation, where *E*_cat_ Prescribes the Coverages of Hydroxyl Moieties that Connect Rates of Half-Reactions

Figure 6a shows the temperature dependences on GNA oxidation rates as a function of the O_2_-to-GNA ratio (O_2_/GNA). Coverages vary widely among these three kinetic regimes as well as with temperature, giving apparent activation enthalpies (Δ*H*_app_^⧧^) that contain contributions of kinetic parameters from both half-reactions (i.e., a convolution of activation enthalpies equaling the difference between C–H scission transition states and H_2_O*, OO*, and HO^–^* initial states). Regression of *–r*_GNA_ against eq 11 at each temperature approximates the first-order GNA oxidation activation enthalpy (Δ*H*_GNA,1st_^⧧^, equaling the sum of GNA adsorption and C–H activation enthalpies Δ*H*_ads,GNA_ and Δ*H*_C–H_^⧧^, respectively, and defined as the difference between C–H scission transition states and HO^–^* saturated Pt initial states):

$$\Delta H_{\mathrm{app}}^{⧧}\approx \Delta H_{\mathrm{GNA},1\mathrm{st}}^{⧧}=\Delta H_{C-H}^{⧧}+\Delta H_{\mathrm{ads},\mathrm{GNA}}+\beta_{C-H}{\mathrm{FE}}_{C-H}^{0}-\Delta H_{\mathrm{Pt}}^{0}$$ 12

*E*_C–H_^0^ and Δ*H*_Pt_^0^ signify the equilibrium potential to heterolytically cleave a C–H bond (Scheme 1, Step G.2a) and the enthalpy required to polarize Pt surface sites by electron donation to HO* (detailed in SI section S14), respectively. Applying transition state theory to all kinetic results of Figure 6a deconvolutes the first-order GNA oxidation enthalpies (Δ*H*_GNA,1st_^⧧^) and entropies (Δ*S*_GNA,1st_^⧧^).

**Figure 6:**
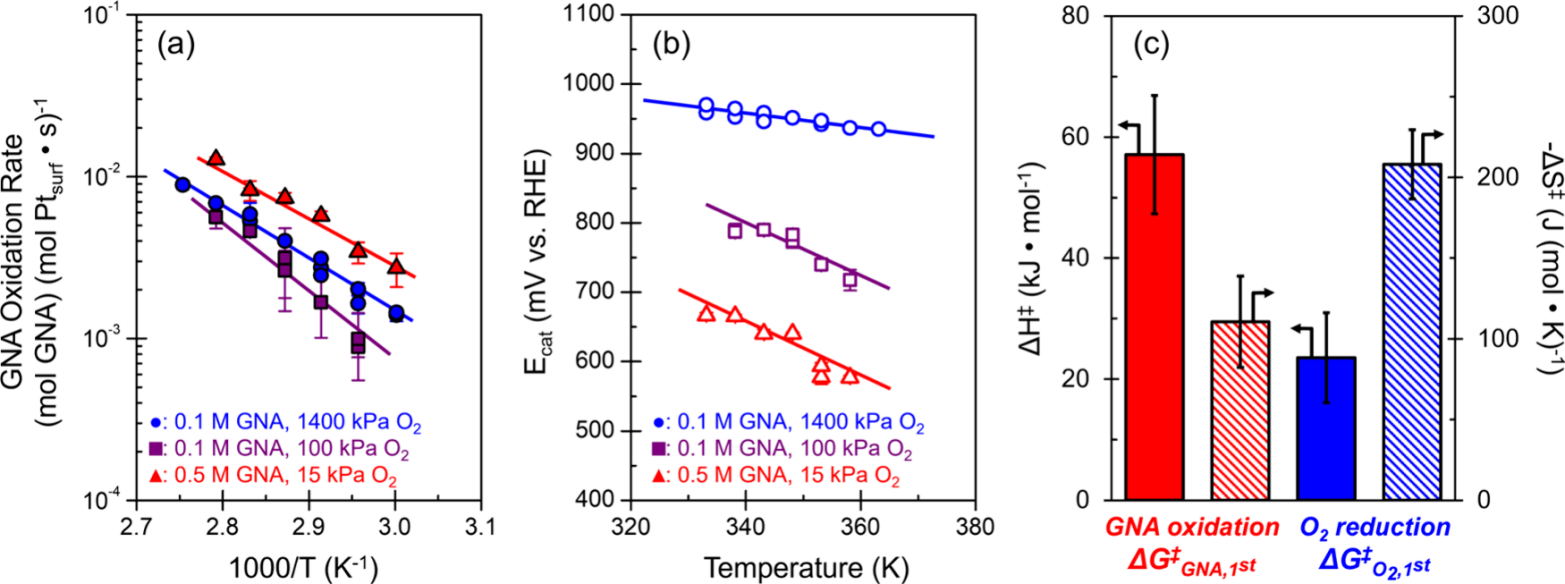
Effects of reaction temperature on kinetics for GNA oxidation including (a) turnover rates for GNA oxidation and (b) catalyst potential *E*_cat_ at distinct GNA and O_2_ feed compositions (blue: 1400 kPa O_2_, 0.1 M GNA, O_2_/GNA = 660; purple: 100 kPa O_2_, 0.1 M GNA, O_2_/GNA = 20; and red: 15 kPa O_2_, 0.5 M GNA, O_2_/GNA = 1). (c) Activation enthalpies (Δ*H* ^⧧^, solid fill) and entropies (Δ*S* ^⧧^, hatched fill) comprising the first-order activation free energies for GNA oxidation (Δ*G*_GNA,1st_^⧧^, red) and O_2_ reduction (Δ*G*_O_2_,1st_^⧧^, blue) derived from panels (a,b).

Figure 6b shows that values of *E*_cat_ decrease as the temperature rises at all combinations of activities for GNA and O_2_ (i.e., *a*_GNA_, a_O_2__), and further, the sensitivity of *E*_cat_ to temperature depends on the ratio of these activities. These consistently decreasing *E*_cat_ values indicate that coverages of HO^–^* decrease with increasing temperature for all combinations of GNA and O_2_ reactants. *E*_cat_ decreases when the first-order activation free energies for GNA oxidation (Δ*G*_GNA,1st_^⧧^) decrease with temperature to a greater extent than those for O_2_ reduction (Δ*G*_O_2_,1st_^⧧^) (eq 9). Calculated values for the temperature sensitivity of *E*_cat_ (*∂E*_*cat*_ */∂T*, slope of Figure 6b) thus depend on the activation barriers and adsorption thermodynamics of the key reactant for each half-reaction. More specifically, values of *∂E*_cat_ */∂T* report directly on first-order activation entropies for each half-reaction (Δ*S*_O_2_,1st_^⧧^ and Δ*S*_GNA,1st_^⧧^, derivation in SI section S15), through the following form:

$$\frac{\partial E_{\mathrm{cat}}}{\partial T}=\frac{\Delta S_{O_{2},1\mathrm{st}}^{⧧}-\Delta S_{\mathrm{GNA},1\mathrm{st}}^{⧧}}{(\beta_{C-H}+\beta_{O-1})F}+\frac{1}{(\beta_{C-H}+\beta_{O-1})F}\;\ln(2\frac{a_{O_{2}}}{a_{\mathrm{GNA}}})$$ 13

where β_C–H_ and β_O__–1_ represent elementary charge transfer coefficients (steps G.2a and O.2, respectively). Most significantly, the values of *∂E*_cat_ */*∂*T* capture differences between Δ*S*_O_2_,1st_^⧧^ and Δ*S*_GNA,1st_^⧧^ that remain independent of the fractional coverages of surface intermediates (and hence require no direct knowledge of these quantities). Rearranging eq 13 isolates Δ*S*_O_2_,1st_^⧧^:

$$\Delta S_{O_{2},1\mathrm{st}}^{⧧}=\Delta S_{\mathrm{GNA},1\mathrm{st}}^{⧧}+(\beta_{C-H}+\beta_{O-1})F\frac{\partial E_{\mathrm{cat}}}{\partial T}-\ln(2\frac{a_{O_{2}}}{a_{\mathrm{GNA}}})$$ 14

Combining Δ*G*_GNA,1st_^⧧^ with *E*_cat_ directly gives Δ*G*_O_2_,1st_^⧧^ at each temperature, and substituting Δ*S*_GNA,1st_^⧧^ (kinetically assessed in Figure 6a) into eq 14 gives Δ*S*_O_2_,1st_^⧧^, enabling a direct measure of the first-order O_2_ activation enthalpies (Δ*H*_O_2_,1st_^⧧^).

Figure 6c compares the enthalpies and entropies that comprise Δ*G*_O_2_,1st_^⧧^ and Δ*G*_GNA,1st_^⧧^. Lower Δ*H*_O_2_,1st_^⧧^ values relative to Δ*H*_GNA,1st_^⧧^ agree with the isotopic assessments (Figure 3) that identify C–H bond scission as the overall kinetically relevant step in GNA-O_2_ reactions. For O_2_ reduction, the highly negative Δ*S*_O_2_,1st_^⧧^ (−208 ± 21 J (mol K)^−1^) remains consistent with an entropy loss of approximately 50% of the combined standard entropy of gaseous O_2_ and H_2_O (205 and 189 J (mol K)^−1^, respectively^52^). The activation enthalpies for C–H scission (Δ*H*_GNA,1st_^⧧^ of 57 ± 10 kJ mol^–1^, versus 40–95 kJ mol^–1^ for methanol oxidation on Pt, strongly dependent on the overpotential^53,54^ ) and O_2_ reduction (Δ*H*_O_2_,1st_^⧧^ of 24 ± 7 kJ mol^–1^, versus 23–40 kJ mol^–1^ for O_2_ reduction on Pt^55–57^ ) agree closely with independent assessments of these values extracted from individual half-reactions examined on separated anodic and cathodic reactions in a traditional three-electrode cell. The approach herein illustrates that tandem examination of rates, activation enthalpies, and *E*_cat_ quantifies kinetic parameters for multiple elementary steps as a technique to abstract additional thermochemical insight into the free-energy landscape of Scheme 2.

Here, we learn that the catalyst potential (*E*_cat_) during partial oxidation of complex oxygenates reports on the coverages of polarized surface intermediates that couple the half-reactions responsible for catalytic turnovers (i.e., HO^–^*, eq 10) through an examination of the thermocatalytic sequence of elementary steps combined with perspective provided by electrokinetic analysis. *E*_cat_ uniquely reports on HO^–^* coverages because HO^–^* serves as a common intermediate between kinetically relevant processes for O_2_ reduction and GNA oxidation half-reactions. The coverages of HO^–^* influence the thermocatalytic GNA oxidation rates (*−r*_GNA_), but these rates do not vary as a single-valued function of *E*_cat_, because GNA also adsorbs and activates on the Pt surfaces. Treatments of the reduction and oxidation of substrates as two independent electrochemical half-reactions provide an accurate description only when the chemistry fulfills several requirements. First, the coverages of all GNA- and O_2_-derived surface intermediates have a similar potential dependence. Second, the coverage of a GNA-derived intermediate does not affect the O_2_ reduction half-reaction rates, and vice versa. Third, all other elementary electrochemical half-reactions occurring in parallel have current densities lower than those of GNA oxidation and O_2_ reduction. These requirements likely do not hold for many situations that involve complex and multifunctional substrates, especially those with high coverages of reactive intermediates. Consequently, rigorous treatment of these systems must address the intermediate coverages of all species, including those formed through elementary steps that do not involve charge transfer, which affect oxidation rates but not *E*_cat_.

Drawing connections to concepts familiar to practitioners of heterogeneous catalysis, values of *E*_cat_ offer a measure of a quantity colloquially described as a virtual fugacity of hydroxyl species on the Pt surface.^41,58^ In some cases such as for elevated pH, these hydroxyl coverages may be equilibrated with the concentration of bulk hydroxide anions (HO^–^) in the fluid phase, as noted for glycerol oxidation on Au.^59^ In this scenario, the quasi-equilibrated desorption of hydroxide equates the virtual and actual fugacity of hydroxyl species, resulting in the equilibration of solution electrochemical potentials with *E*_cat_. Virtual fugacities represent the number of intermediates generated *in situ*, which exist at numbers difficult to assess in complex reactions and remain governed by activities of fluid-phase reactants but consider all the coverage-dependent kinetics of reactions that form and consume these intermediates at pseudo-steady-state. Here, simultaneous *E*_cat_ assessments during thermocatalysis enable a rapid quantification of HO^–^* coverage without requiring extensive steady-state kinetic dependencies (exceeding 300 h on stream) and during transient kinetic periods, especially relevant for understanding catalyst deactivation.

### 3.5 Method to Increase Time-Averaged Turnover Rates Inspired by Scaling Relationships between Rates and *E*_cat_

Values of *E*_cat_ reflect the coverages of reactive surface intermediates but do not correspond to a single value of the GNA oxidation turnover rate (Figures 5 and 6). These observations, together with knowledge of how unreactive spectator reaction intermediates inhibit rates,^4^ suggest a methodology to increase the productivity of Pt nanoparticle catalysts for GNA oxidation reactions over extended periods by manipulating *E*_cat_.

Figure 7a shows that GNA oxidation rates decrease by approximately 50% over the first 48 h of time on stream following the initiation of the reaction (0.12 M GNA, 100 kPa O_2_, 353 K). Values of *E*_cat_ stabilize within the first 3–10 h of this process (∼750 mV_RHE_) and remain constant over greater periods, because rates of both GNA oxidation and O_2_ reduction half-reactions decrease symmetrically over the same Pt-active sites. These decreases in rates at nearly constant *E*_cat_ indicate that changes in reactivity reflect the loss of Pt surface atoms available for catalysis by aggregation or sintering of Pt nanoparticles, dissolution and leaching of Pt from the carbon support, or increasing coverages of unreactive surface species. The quantity of Pt on the carbon support and the mean diameter of Pt clusters remain mostly unchanged following catalysis for extended periods (>400 h on stream, Table 1), which excludes leaching and aggregation as the predominant cause of this initial deactivation. Furthermore, intermittent changes to the reaction conditions led to significant increases in transient rates, inconsistent with site loss by Pt aggregation. Figure 7a shows immediate decreases in both GNA oxidation rates (6 ± 1 to <0.01 (mmol GNA) (mol Pt_surf_ s)^−1^) and *E*_cat_ (750 to <200 mV_RHE_) in response to removal of O_2_ from the reactor inlet (i.e., 101 to 0 kPa O_2_ and 101 kPa Ar). The reintroduction of O_2_ after 9 h causes GNA oxidation rates to rise and exceed the initial steady-state values, while *E*_cat_ nearly instantaneously returns to the original value (750 mV_RHE_). Subsequently, the system exhibits a decrease in rates akin to that observed during the initial induction period and reaches equal steady-state rates after 30 h. This behavior suggests that the decrease in values of *E*_cat_ with removal of the O_2_ reflects a change in the coverages of reactive intermediates, corresponding to removal of inhibiting surface intermediates from active sites on the Pt nanoparticles.

**Figure 7:**
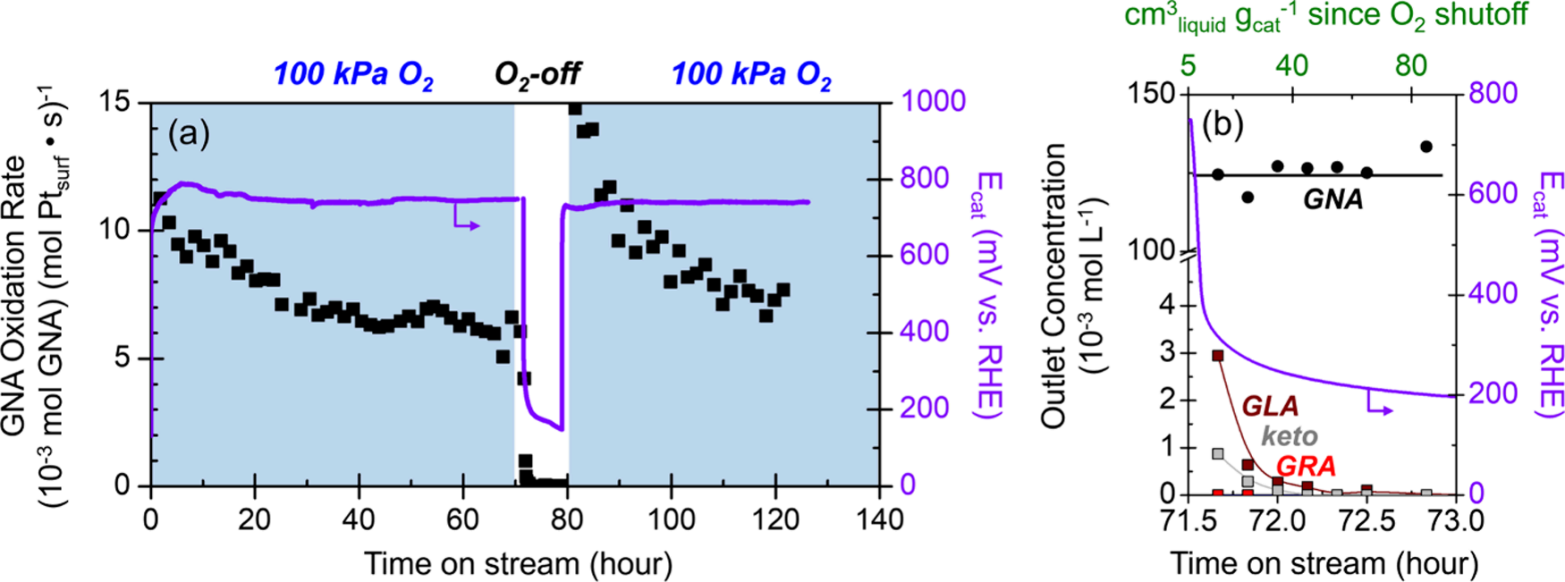
(a) GNA oxidation rates (■) and catalyst open-circuit potentials (purple) versus time on stream with 100 kPa O_2_ (blue shaded) or 100 kPa Ar (no O_2_, unshaded) (0.12 M GNA, 353 K). (b) Outlet concentrations of GNA (●) and major products guluronic acid (GLA, brown), glucaric acid (GRA, red), and keto-glucaric acids (keto, gray) during the initial period of O_2_ shutoff, alongside the cumulative liquid passed over the catalyst bed since the O_2_ shutoff as a second *x*-axis (green).

Figure 7b shows that products from GNA oxidation slowly elute from the reactor over the course of approximately 1 h following the removal of O_2_ from the inlet stream, which corresponds to a much longer time scale than needed to purge gaseous or dissolved O_2_ from the system (cumulative liquid volumes passed through the catalyst bed in 1 h exceed 60 cm_liquid_^3^ g_cat_^–1^, second *x*-axis of Figure 7b, and with 100-fold shorter gas residence times, the cumulative gas volumes passed through the catalyst bed exceed 6000 cm_gas_^3^ g_cat_^–1^). These comparisons show that reactions of GNA with residual O_2_-derived intermediates cannot form the GLA, GRA, and keto products that eluted over this period. Consequently, these products must form and accumulate on the surface during steady-state catalysis but then desorb from the catalyst surfaces after the O_2_ shutoff. The integrated quantities of GNA, GRA, and 2-keto/5-keto products that elute during the O_2_ shutoff period represent approximately 6 mol_O‑consumed_ mol_Pt–surf_^–1^, which far exceeds the expected quantity that could be stabilized on Pt nanoparticles alone and inconsistent with a reduction of platinum oxides as proposed previously.^60,61^ Hence, these findings suggest that these organic species bind both on Pt nanoparticles and across the conductive carbon support, and while reactions on Pt determine *E*_cat_, changes in *E*_cat_ affect electrostatic forces within the entire conductive catalyst bed. Similar transient behavior of rates and *E*_cat_ values appear in experiments that interrupt the O_2_ flow for periods of 0.5 and 1.0 h (SI section S16), which all show integrated transient product quantities of 4–6 mol_O‑consumed_ mol_Pt–surf_^–1^.

These behaviors suggest the inhibiting organic residues desorb in response to a decrease in *E*_cat_ (toward more cathodic values) and a corresponding diminishment of electrostatic forces, as portrayed in Scheme 3. During steady-state catalysis, *E*_cat_ values remain more positive than 600 mV_RHE_, which remains above the potential of zero charge for Pt nanoparticles (400–500 mV_RHE_ at pH 2.5, 298 K^62,63^ ) and carbon (likely between 150 and 300 mV_RHE_ at pH 2.5 based on the work function^64^), noting that these potential of zero charge values depend highly on coverages.^65^ As a result, the surfaces remain positively polarized and bind anionic carboxylates strongly at high coverages. These species compete for active sites and reduce the coverage of HO^–^*, RCH_2_O­(H)*, and other key intermediates, which leads to a decrease in reaction rate. Upon interrupting O_2_ flow, the *E*_cat_ decreases and may fall below the potential of zero charge, negatively polarizing the Pt and carbon surfaces. The change in polarity destabilizes carboxylate adsorbates, which causes spectator inhibiting species to desorb and increase the fraction of Pt-active sites available to bind other reactive species as well as facilitate O_2_ activation and reduction. This leads to higher catalytic turnover rates once O_2_ re-enters the reactor. This dependence of carboxylate adsorption on the catalyst potential reflects one instance in which a thermochemical parameter depends on spontaneously generated electric fields, even without net electrochemical reactions and electrolytes.

**Scheme 3:**
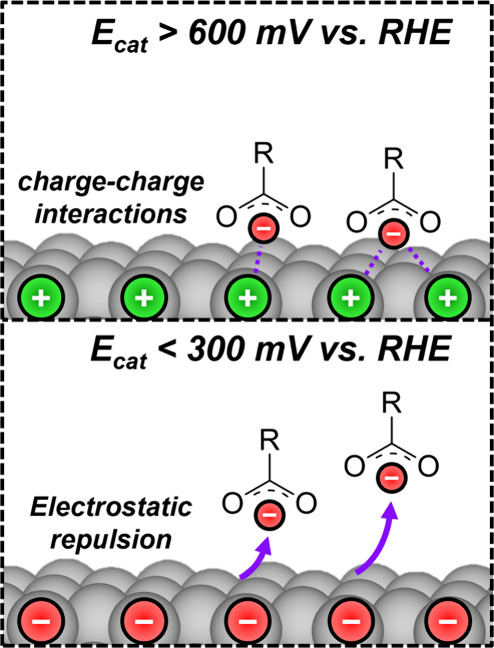
Proposed Interactions between Anionic Carboxylate Species and the Pt/C Surface above and below the Potential of Zero Charge

Figure 7 shows that the increased reaction rates persist on a much longer time scale than the duration of the O_2_ shutoffs: periodically interrupting O_2_ flow may offer a method to repeatedly regenerate active sites and when optimized increase the time-averaged productivity of these reactors. Notably, we determined that shutting off the O_2_ for 0.5 h followed by operating at standard conditions for 6 h (0.12 M GNA, 100 kPa O_2_, 353 K) leads to a time-averaged GNA oxidation rate that surpasses that for steady-state operation by 50% (7.2 × 10^–3^ vs 4.7 × 10^–3^ (mol_GNA_) (mol Pt_surf_ s)^−1^). SI section S17 describes the approach used to optimize the performance. These findings suggest that further improvements may be achieved through more sophisticated control of O_2_ flow rates and partial pressures that would provide dynamic operation, and *in situ* measurements of *E*_cat_ would offer high time resolution insight needed in programming this process. Generally, time-averaged GNA oxidation rates on Pt increase with shorter O_2_ shutoff times and more frequent regeneration cycles, leaving room for further optimization. These rate enhancement methods apply for a wide range of polyol oxidation reaction systems as long as those reactions generate an anionic product that strongly adsorbs on the metal surface at steady state (for *E*_cat_ above the potential of zero charge) but with weak adsorption in the absence of O_2_ (when *E*_cat_ falls below the potential of zero charge).

## 4 Conclusions

In this study, we combine thermocatalytic and electrocatalytic analyses to reveal the elementary steps of gluconic acid (GNA)-O_2_ reactions on Pt catalyst surfaces and describe how the half-reactions within aqueous aerobic polyol oxidations combine to complete a catalytic cycle. Kinetic and isotopic experiments with *in situ* measurements of the catalyst potential at open circuit (*E*_cat_) bridge the gap between thermo- and electrocatalysis by tracking the coverages of surface intermediates during steady-state reactions. This analysis holds in a trickle-bed reactor at elevated temperatures (above 333 K) and pressures (including up to 2800 kPa O_2_), in which direct electrochemical measurements are infeasible.

From a thermocatalytic lens, a set of elementary steps describes the GNA oxidation rates in which C–H scission is kinetically relevant. These elementary steps contain irreversible O_2_ reduction and GNA oxidation half-reactions (confirmed by isotopic assessments) where the balance between oxidized (HO*) and reduced (*) Pt sites kinetically couples the half-reactions. Over a wide range of industrially relevant reaction conditions (0.03–0.95 M GNA, 20–2800 kPa O_2_, 333–363 K), the coverages of both O_2_- and GNA-derived intermediates change dramatically, captured in a microkinetic model consistent with the elementary steps.

From an electrocatalytic lens, *E*_cat_ depends monotonically on the instantaneous O_2_-to-GNA molar ratio over 3 orders of magnitude despite the widely changing coverages, with elementary O_2_ reduction and GNA oxidation transfer coefficients (β_O__–1_ and β_C–H_) that sum to 0.6 ± 0.1 indicating their individual transfer coefficients fall well below 0.5. The same elementary steps that capture the thermocatalytic rates also describe *E*_cat_ once explicitly denoting charge transfer via heterolytic [*^–^O­(H)···H^+^···C­(H)­(R)–O­(H)*]^⧧^ transition states (*R* = C_5_H_9_O_6_). All charge transfer elementary steps can contribute equally to *E*_cat_, such that no single pair of elementary steps sets the potential. From these elementary steps, *E*_cat_ predominantly describes the coverages of polarized surface intermediates (HO^–^*), but a single *E*_cat_ corresponds to multiple rates depending on the coverages of the other reactive surface intermediates. Furthermore, temperature dependencies on *E*_cat_ reveal that HO^–^* coverages decrease with temperature, ascribed to higher activation entropies for GNA oxidation relative to O_2_ reduction. Leveraging this knowledge, we observed that O_2_ shutoffs decrease *E*_cat_ by desorbing strongly bound anionic carboxylate species, regenerating deactivated Pt sites and increasing the integral GNA oxidation performance.

Taken together, these findings reveal the fundamental connection between the catalyst potential, the thermochemical rates, and the coverages of surface intermediates during steady-state redox reactions. *E*_cat_ can serve as an *operando* probe in thermocatalysis for the precise surface intermediates that couple reduction and oxidation half-reactions. The methods employed here for the specific case of GNA oxidation can be generalized: any set of reactions described by mixed potential theory (e.g., aerobic oxidations for formic acid, ethanol, furfural, or γ-hydroxybutyric acid) could conceivably be described at elevated temperature and pressure by a single sequence of elementary steps that captures the thermocatalytic rates, the coverages of surface species, and the catalyst potential. Furthermore, manipulating *E*_cat_ precisely tunes the surface coverages, which provides an additional method to manipulate the reactivity of metal catalysts for aqueous aerobic oxidation chemistry.

## Supplementary Material



## Notes

Transmission electron micrographs; effect of sodium perchlorate on *E*_cat_; GC chromatogram; batch GNA oxidation kinetics; Pt-active site confirmation for Pt/C; representative time on stream GNA oxidation rates and *E*_cat_; effect of liquid hourly space velocity; hydrogen underpotential deposition displacement by GNA; hydrogen–deuterium isotope effects on GNA and GHB oxidation rates; ^16^O_2_–H_2_^18^O isotope exchange and ^18^O incorporation into GNA; electrochemical polarization curves; derivations of GNA oxidation rate expressions and *E*_cat_ expressions; comparison of GNA oxidation microkinetic models; derivations of temperature dependences of GNA oxidation rates and *E*_cat_; effects of varying O_2_ shutoff time on GNA oxidation rates and *E*_cat_; mathematical optimization of reactor productivity from O_2_ shutoffs (PDF)

## †

Department of Chemical and Biomolecular Engineering, KAIST, 9986+GH Daejeon, South Korea
