# Supplementary material for: Catalyst Potential Prescribes Intermediate Coverages in Thermocatalytic Gluconic Acid Oxidation on Pt Nanoparticles
Source: J Am Chem Soc. 2026 May 1;148(18):18746–61. doi: 10.1021/jacs.5c22029 (PMC13185123; doi:10.1021/jacs.5c22029)
Supplement: Supplementary file 1 [file ja5c22029_si_001.pdf]

Catalyst Potential Prescribes Intermediate Coverages in Thermocatalytic Gluconic Acid Oxidation on Pt Nanoparticles

Supplementary Information

William Thomas Broomhead,[1] Minju Chung,[1] Karl O. Albrecht,[2] and David W. Flaherty[1,*]

1. *School of Chemical and Biomolecular Engineering, Georgia Institute of Technology, Atlanta, GA 30032, United States*
2. *ADM Research and Development Center, Archer Daniels Midland Company, Decatur, IL 62521, United States*

* Corresponding author. dflaherty3@gatech.edu

S1. Transmission Electron Micrographs of Pt/C Catalysts S2
S2. Effects of Sodium Perchlorate on Catalyst Potential for Pt/C S3
S3. Representative Gas Chromatograph from Gluconic Acid Oxidation on Pt/C S4
S4. GNA-O_2 Product Evolution during Batch Kinetic GNA Oxidation Experiments for Pt/C S5
S5. Confirmation of Pt as the Active Sites for GNA Oxidation on Pt/C Catalysts S6
S6. Time on Stream Evolution of GNA Oxidation Rates and Open Circuit Potential S7
S7. GNA Oxidation Rates as a Function of LHSV during Steady State GNA-O_2 Reactions S9
S8. Estimates of GNA Adsorption Equilibrium Constants at 293 K by Cyclic Voltammograms S10
S9. Batch and Flow Reactor Kinetic Profiles for GHB and GNA Oxidation Isotope Effect Experiments on Pt/C S11
S10. $^{16}O_2$-$H_2{}^{18}O$ Labeling and Electrochemical Potentiometry to Probe O_2 Reduction Reversibility S12
S11. Mass Spectra of GNA and GRA from Reactions with $^{16}O_2$ and $H_2{}^{18}O$ S13
S12. Pseudo Steady State Derivations of Rate Equations from Thermocatalytic Elementary Steps S14
  Derivation of a General Rate Equation S14
  Alternative Case 1: O_2 Reduction Requires Paired Active Sites S17
  Alternative Case 2: Quasi-Equilibrated O-H Scission Precedes C-H Scission S20
  Alternative Case 3: C-H Scission Forms a Metal Hydride S24
S13. Linear Regression and Fitting of Microkinetic Models for GNA Oxidation S27
S14. Pseudo Steady State Derivations of Rate Equations and Catalyst Potential Expressions from the Electrocatalytic Analogues of Thermocatalytic Elementary Steps S31
  Derivation of a General Rate Equation S32
    Complete Electron Balance S33
  Alternative Case 1: C-H Scission is Solution Mediated S37
S15. Derivation of the Enthalpic and Entropic Contributions to Rates and E_{cat} S45
S16. GNA Oxidation Transient Rate and Potential Profiles with Varying O_2 Shutoff Times S50
S17. Optimizing Integral Reactor Productivity with Periodic O_2 Shutoffs S51
S18. References S53

S1. Transmission Electron Micrographs of Pt/C Catalysts

Figure S1 provides the Pt cluster diameter distributions and representative micrographs from transmission electron microscopy (TEM) of pre- and post-reaction Pt/C catalysts. Average Pt cluster diameters increase after 400 hours of GNA oxidation reactions. Assuming the Pt clusters are hemispherical with bulk metal crystallographic properties,[1] these diameters give an average dispersion of 26% before reactions and 17% after reactions.

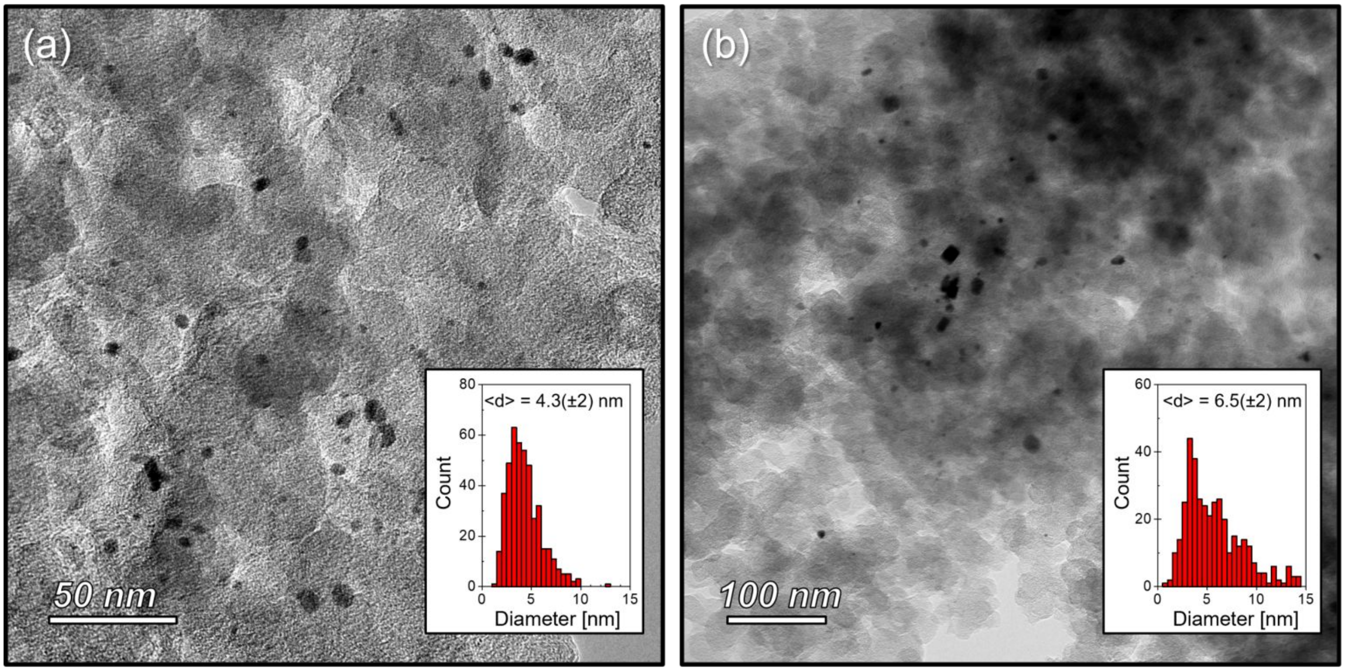

Figure S1. Transmission electron micrographs of (a) pristine Pt/C catalysts and (b) those after 400 hours of catalytic operation in GNA-O_2 reactions (0–0.95 M GNA, 20–2800 kPa O_2, 353–363 K). Cluster diameter histograms included as an inset.

S2. Effects of Sodium Perchlorate on Catalyst Potential for Pt/C

Figure S2 provides GNA oxidation rates and open circuit potential values of Pt/C catalysts in the presence of $NaClO_4$ as an additional ionic component. Catalyst potential varies less than 20 mV_{RHE} among all conditions (0 to 0.5 M $NaClO_4$) indicating that GNA feeds alone contain sufficiently high ionic conductivity to quantitatively assess E_{cat} (ionic strength of GNA alone of approximately 0.005 M, measured pH of 2.2). These variations fall within the reported error margin. GNA oxidation rates remain approximately constant with 0.1 M $NaClO_4$ but irreversibly decrease with 0.5 M $NaClO_4$. These rate decreases may arise from the electrostatic adsorption of Cl^- impurities present in the $NaClO_4$.

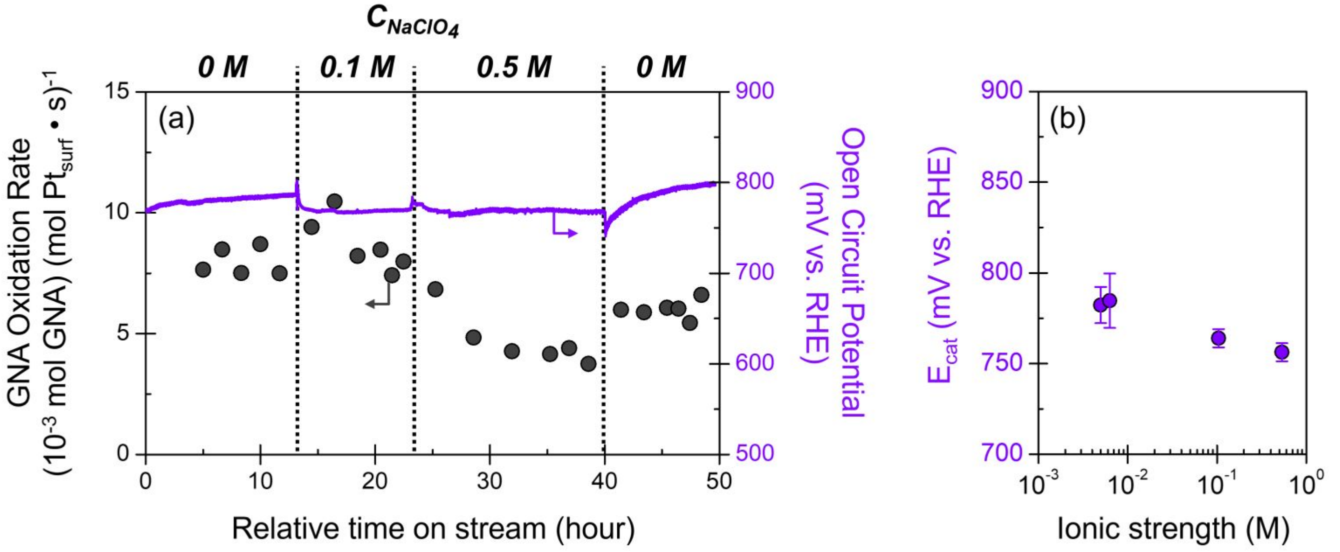

Figure S2. (a) GNA oxidation rates and catalyst open circuit potentials on Pt/C with the introduction of sodium perchlorate (0.1 and 0.5 M) at intermediate times on stream. (b) catalyst potential E_{cat} versus the total ionic strength. (353 K, 0.12 M GNA, 1400 kPa O_2, 0.2 g Pt/C, pH 2.3).

S3. Representative Gas Chromatograph from Gluconic Acid Oxidation on Pt/C

Figure S3 provides a section of a representative gas chromatogram during gluconic acid (GNA)-O_2 reactions on Pt/C operating at 20% GNA conversion. The abbreviations are for 2-keto-gluconic acid (2-keto), 5-keto-gluconic acid (5-keto), guluronic acid (GLA), gluconic acid (GNA), and glucaric acid (GRA). Smaller molecules (4- and 5-carbon products, e.g., tartaric acid, as well as the solvent, excess silylation agent, and 1,3-butanediol internal standard) elute before 29 minutes.

This gas chromatogram was generated using a Zebron ZB-5 column (60 m × 0.32 mm × 0.25 μm), H_2 as the carrier gas at a constant linear velocity of 40 cm sec^{-1}, and a temperature program of: 60°C for 2 minutes, 76°C (8°C min^{-1}) for 0 minutes, 124°C (24°C min^{-1}) for 0 minutes, 166°C (6°C min^{-1}) for 0 minutes, 206°C (2°C min^{-1}) for 0 minutes, and 330°C (24°C min^{-1}) for 4 minutes.

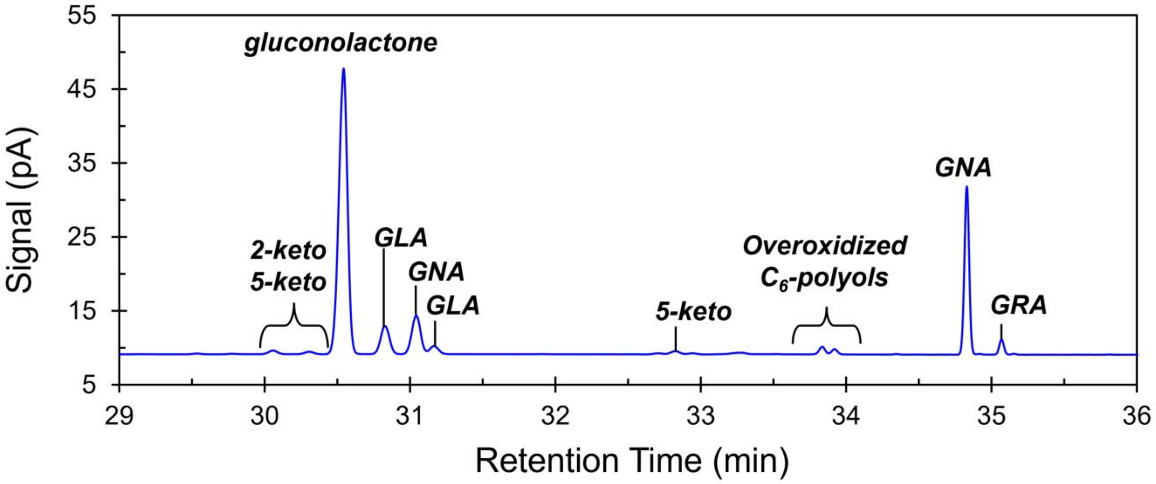

Figure S3. Representative section of a GC chromatogram from GNA-O_2 reactions on Pt/C catalysts, operating at approximately 20% GNA conversion (0.12 M GNA, 1400 kPa O_2, 353 K).

S4. GNA-O_2 Product Evolution during Batch Kinetic GNA Oxidation Experiments for Pt/C

Figure S4a shows the concentrations of guluronic acid (GLA), glucaric acid (GRA), and 2- and 5-keto gluconic acids (keto) versus site time (units of $mol_{Pt\text{-}surf}$ s L^{-1}) during batch GNA-O_2 reactions on Pt/C with varying catalyst masses. The initial slopes of these batch profiles give the site time yields of each product. The total GNA oxidation turnover rates ($-r_{GNA}$) derived from these batch profiles match those of the flow reactor experiments under identical conditions (8±1 mol ($mol_{Pt\text{-}surf}$ s)$^{-1}$, 0.12 M GNA, 1600 kPa O_2, 353 K). Figure S4b overlays the integral carbon selectivities as a function of GNA conversion during the batch kinetic experiments with those of the flow reactor (Figure 1b of the main text), with identical liquid and gas phase compositions. While batch experiments generally exhibit a higher GLA selectivity compared to steady state experiments (likely due to reactions that occur on fresh Pt surfaces versus those that have deactivated on-stream), the same conversion-selectivity trend holds.

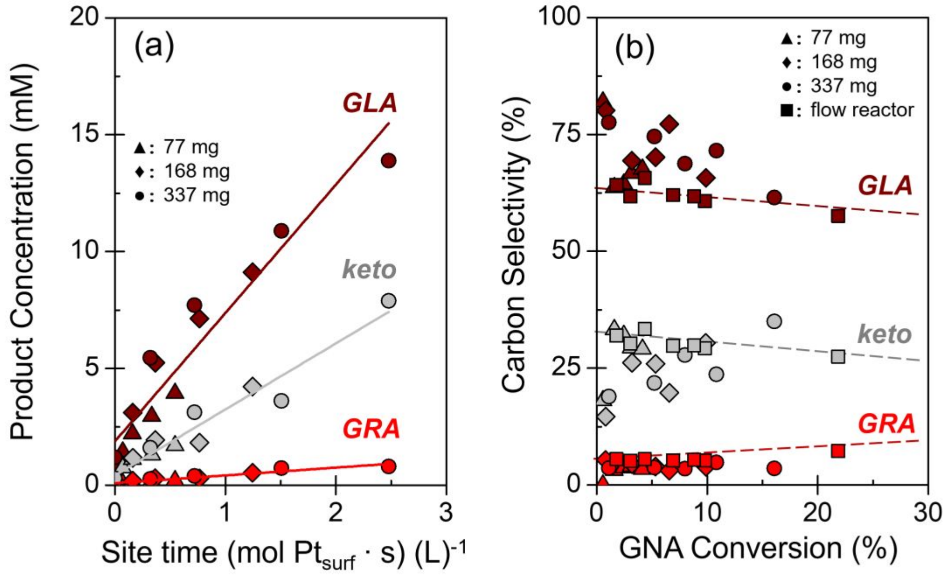

Figure S4. Oxidation of GNA in semi-batch reactors and corresponding (a) product concentrations as a function of site time, (b) carbon selectivities as a function of GNA conversion (guluronic acid (GLA, brown), glucaric acid (GRA, red), keto-gluconic acids (keto, gray), 0.12 M GNA (30–35 cm^3_{liquid}), 1600 kPa O_2 (45 cm^3_{vapor}), 353 K at 600 RPM). Measurements obtained with total loadings of 77 mg (▲), 168 mg (♦), and 337 mg (●) of Pt/C catalysts in semi-batch reactors and with 750 mg (■) Pt/C catalyst in trickle bed reactors.

S5. Confirmation of Pt as the Active Sites for GNA Oxidation on Pt/C Catalysts

Figure S5 provides electrochemical polarization curves for O_2 reduction (ORR) and GNA oxidation (GOR) half-reactions on bare Vulcan XC-72R carbon and 20%Pt/C electrodes (0.5 M GNA, 0.1 M perchloric acid solutions saturated by 1 bar O_2). Absolute values of the measured currents (in mA) were normalized by the catalyst mass to give current densities in A g_{cat}^{-1}. The onset potentials for both ORR and GOR half-reactions on carbon alone occur at overpotentials at least 400 mV greater than Pt/C (onset potential defined at 10^{-2} A g_{cat}^{-1}), confirming that both half-reactions occur at Pt nanoparticles. Furthermore, introducing GNA blocks underpotentially-deposited hydrogen on Pt (*vide infra*, Section S8), confirming that GNA chemically adsorbs on the Pt nanoparticles.

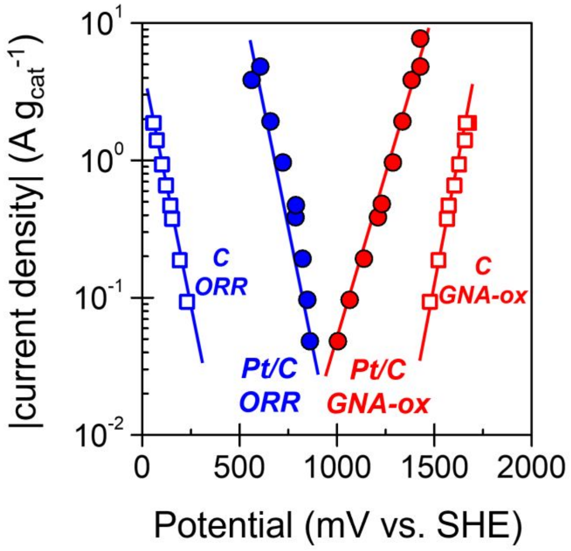

Figure S5. Rates of electron consumption and generation in O_2 reduction (ORR) and GNA oxidation (GOR) electrochemical half-reactions as a function of potential on Pt/C (solid) and bare C (hollow) catalysts, derived from stepwise voltammetry profiles (293 K, 0.1 M $HClO_4$ (0.15 L, pH 1.0), 0.5 M GNA, 1 bar O_2 (1200 cm^3 h^{-1} sparge), 600 RPM, 2.6–3.0 Ω_s).

S6. Time on Stream Evolution of GNA Oxidation Rates and Open Circuit Potential

Figure S6 shows representative time on stream profiles for Pt/C catalysts during GNA-O_2 reactions (0.1 M GNA, 1400 kPa O_2) on two timescales: an induction period during the first 60 hours of time on stream at 363 K (Figure S6a), and a longer period extending to over 300 hours of time on stream at 353 K (Figure S6b). During the initial period, GNA oxidation rates follow a first-order deactivation profile and decrease by approximately 30% over the course of 48 hours on stream (Figure S6a), before reaching a stable rate of 9×10^{-3} mol (g-atom-Pt_{surf} s)$^{-1}$. We ascribe this 48 hour induction behavior to the slow accumulation of surface intermediates, eventually reaching a steady state coverage. Simultaneously, the open circuit potential increases from approximately 0.200 V_{RHE} (i.e., from Pt surfaces without O_2-derived species) to its steady state mixed potential of 0.930 V_{RHE} for these conditions. These changes reflect gradual changes in the coverages of surface intermediates that evolve from irreversible carboxylate adsorption as well as changing coverages of oxygen- and GNA-derived species. Thus, steady state rates were not reported until these induction periods subsided. The decreases in active site density with time give an apparent site density $[L]_{app}$ that equals the initial site density $[L]_{initial}$ minus the coverage of irreversibly-bound adsorbates that contribute to the loss of sites, $[L]_{loss}$:

$$[L]_{app} = [L]_{initial} - [L]_{loss} \tag{S6.1}$$

In all kinetic results reported herein, we normalize turnover rates by $[L]_{initial}$, which reflects turnover rates on $[L]_{app}$ when $[L]_{loss}$ remains constant.

After this initial induction period, the rates decrease by 30% over the course of 250 hours, as demonstrated by periodically returning to a reference condition (Figure S6b). This slow deactivation may arise from irreversible surface adsorbates, dilute contaminants from the reactor feed, or structural changes of Pt nanoparticles, which all increase $[L]_{loss}$. To correct for further changes in $[L]_{loss}$ in this second regime, we take the rate ratio of all kinetic and temperature dependence rates relative to the nearest reference condition of Figure S6b, following previously described methodologies.[2] The resulting rate ratios were then multiplied by the rate of the reference condition at 60 hours on stream (i.e., the initial data reported in Figure S6b) to give the corrected site densities $[L]$, and thus corrected GNA oxidation rates.

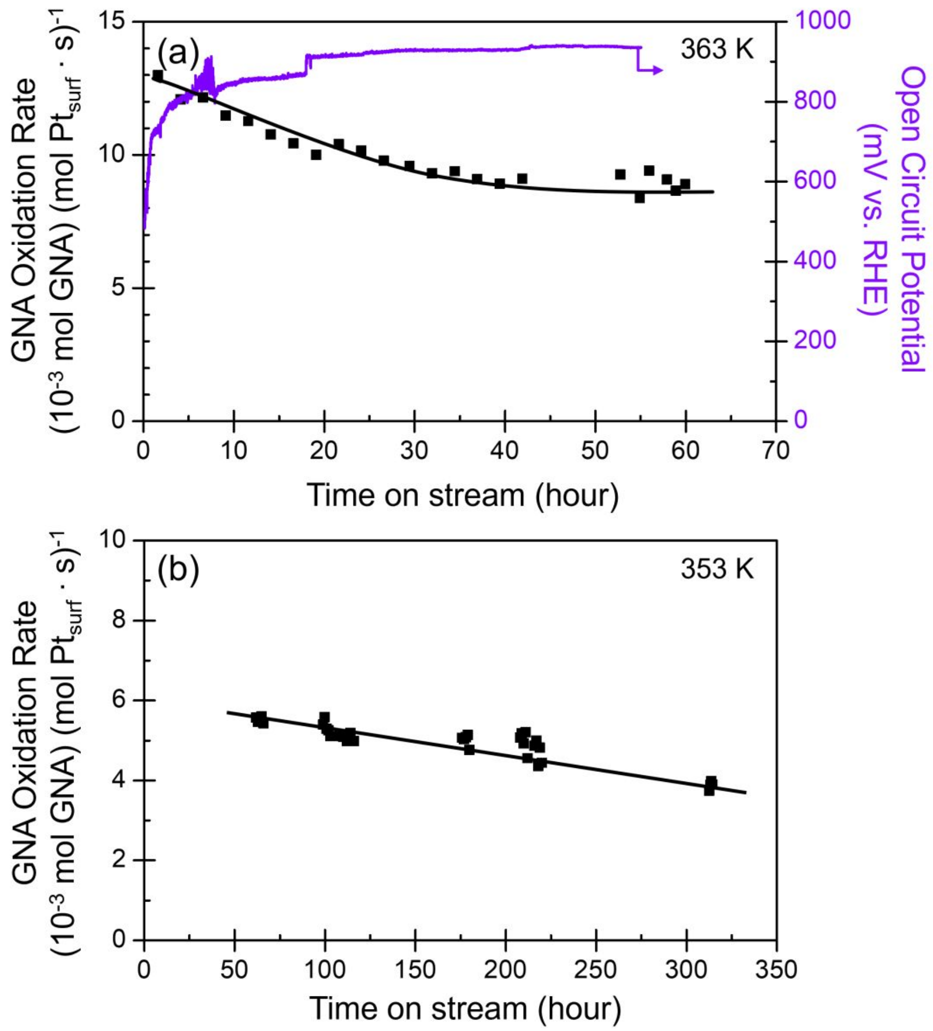

Figure S6. Steady state GNA oxidation rates and catalyst open circuit potentials on Pt/C as a function of time on stream, in (a) the induction period (363 K) and (b) over longer timescales (353 K), 0.12 M GNA (30 cm^3_{liquid} h^{-1}), 14 bar O_2 (3000 cm^3_{STP} h^{-1}), 0.75 g Pt/C, pH 2.5.

S7. GNA Oxidation Rates as a Function of LHSV during Steady State GNA-O_2 Reactions

Figure S7 provides the GNA oxidation turnover rates as a function of the liquid hourly space velocity (LHSV, units of cm^3_{liquid} (g_{cat} h)$^{-1}$) during steady state GNA-O_2 reactions (0.12 M GNA, 1400 kPa O_2, 353 K). Increasing LHSV decreased the GNA conversion regardless of method for achieving the change (either by increasing the liquid volumetric flow rate or by decreasing the Pt/C catalyst mass). Above 20 c m^3_{liquid} (g_{cat} h)$^{-1}$ (below 10% GNA conversion), constant GNA oxidation rates indicate that external mass transport contributes negligibly to the measured rates.

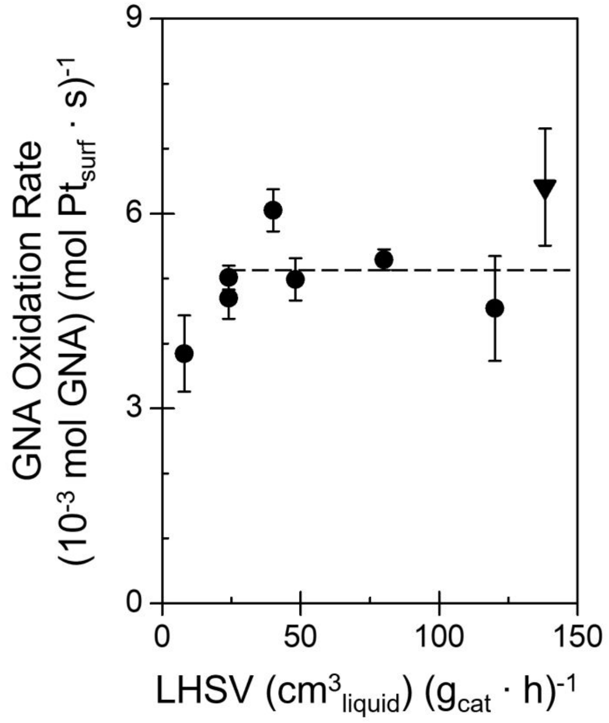

Figure S7. GNA oxidation turnover rates as a function of liquid hourly space velocity (LHSV, cm^3_{liquid} (g_{cat} h)$^{-1}$) for loadings of 0.2 g (▼) and 0.75 g (●) Pt/C catalyst (0.12 M GNA (6–90 cm^3_{liquid} h^{-1}), 1400 kPa O_2 (3000 cm^3_{STP} h^{-1}), 353 K).

S8. Estimates of GNA Adsorption Equilibrium Constants at 293 K by Cyclic Voltammograms

Figure S8 shows cyclic voltammograms of Pt/C catalysts with varying GNA concentrations in 0.1 M perchloric acid solutions, in the regime between 0.08–0.45 V_{RHE}, corresponding to underpotentially-deposited hydrogen on the Pt surface (H_{UPD}-Pt). Integrating the H_{UPD} desorption feature relative to the capacitive current and without any GNA gives a hydrogen coverage of approximately 0.7 H_{UPD} Pt_{surf}^{-1} for 3 nm Pt clusters. H_{UPD} cannot fully saturate the Pt surface, consistent with previous studies.[3,4] The H_{UPD} integral decreases with increasing GNA concentration following an adsorption isotherm (included as the inset) similar to previous findings for phenol and benzaldehyde adsorption on Pt.[5] Fitting a Langmuir adsorption isotherm to the fraction of H_{UPD} inhibited as a surrogate of coverage gives a GNA adsorption equilibrium constant on the order of 100 L mol^{-1}, consistent with a high fractional coverage of GNA-derived surface intermediates.

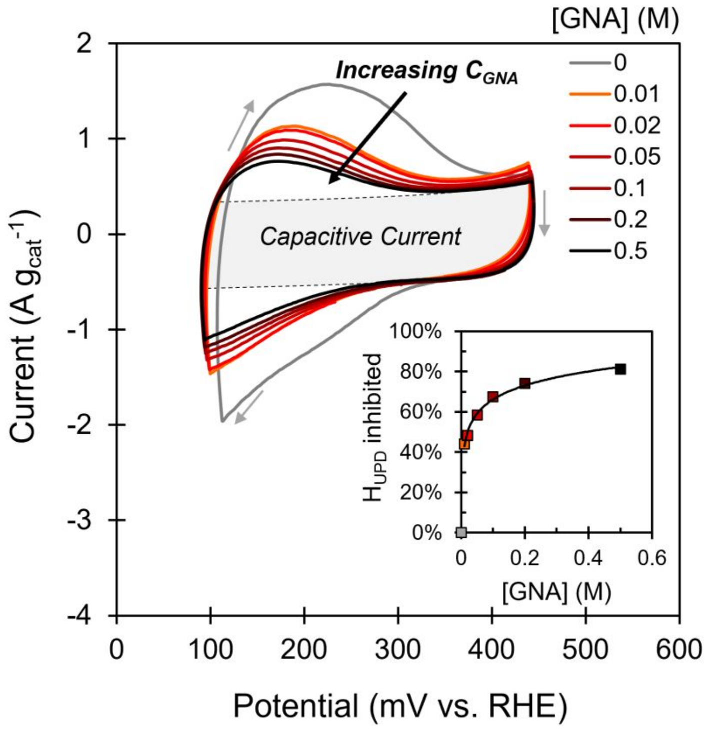

Figure S8. Cyclic voltammograms of Pt/C catalysts from 0.08 to 0.45 V_{RHE} at varying concentrations of gluconic acid (GNA), with an inset showing the fraction of underpotentially-deposited hydrogen (H_{UPD}) inhibited by GNA as a function of GNA concentration, 10 mV s^{-1}, 0.1 M $HClO_4$ (0.15 L, pH 1.0), 0–0.5 M GNA, Ar sparge (600 cm^3 h^{-1}), 10 mg Pt/C catalyst, 293 K, 600 RPM, 2.6–3.0 Ω_s, manual iR compensation.

S9. Batch and Flow Reactor Kinetic Profiles for GHB and GNA Oxidation Isotope Effect Experiments on Pt/C

Figure S9a provides batch concentration profiles during GHB oxidation on Pt/C for kinetic isotope effect (KIE) experiments with fluid phase compositions that differ between GHB + H_2O, D_6-GHB + H_2O, and GHB + D_2O. The concentration of succinic semialdehyde (the primary partial oxidation product of GHB) increases linearly with site time, with slopes corresponding to the turnover rates. Figure S9b verifies the large H_2O/D_2O KIE for GHB oxidation at steady state by varying liquid feeds between GHB + H_2O and GHB + D_2O. Figure S9c provides a similar experiment as Figure S9b, but during GNA oxidation, with liquid feeds of either GNA + H_2O or GNA + D_2O. Within the experimental error, E_{cat} values do not systematically change from H_2O to D_2O feeds. The turnover rates and subsequent kinetic isotope effects (KIE) derived from all three figures are summarized in Figure 3 of the main text.

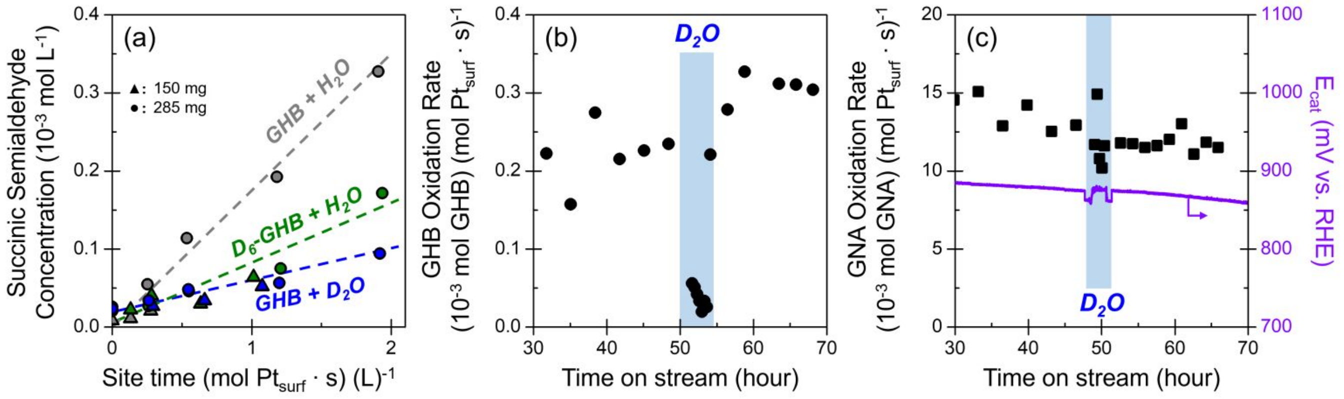

Figure S9. (a) Concentrations of succinic semialdehyde as a function of site time from batch γ-hydroxybutyric acid (GHB) oxidation reactions with GHB/H_2O (gray), D_6-GHB/H_2O (green), and GHB/D_2O (blue) liquid compositions (0.10 M GHB (30–35 cm^3_{liquid}), 1600 kPa O_2 (45 cm^3_{vapor}), 30–35 c m^3_{liquid} 0.15–0.29 g Pt/C, 353 K, 600 RPM). (b) Steady state GHB oxidation rates with GHB/H_2O or GHB/D_2O (blue shaded region) (0.10 M GHB, 1400 kPa O_2, 353 K). (c) Steady state GNA oxidation rates and E_{cat} values with GNA/H_2O or GNA/D_2O (blue shaded region) (0.12 M GNA, 1400 kPa O_2, 353 K).

<u>S10. $^{16}O_2$-$H_2$$^{18}O$ Labeling and Electrochemical Potentiometry to Probe O_2 Reduction Reversibility</u>

Figure S10a provides the O_2 isotopologue distribution ($^{16}O{=}^{16}O$, $^{16}O{=}^{18}O$, and $^{18}O{=}^{18}O$ corresponding to m/z$^+$ of 32, 34, and 36, respectively) during $^{16}O_2$-$H_2$$^{18}O$ batch kinetic experiments with and without 0.14 M GNA, alongside $^{16}O_2$ and $^{18}O_2$ as references. In both cases, $^{16}O{=}^{18}O$ and $^{18}O{=}^{18}O$ were not detected, indicating that the O_2 reduction half-reactions are irreversible.

Figure S10b provides electrochemical polarization curves for O_2 reduction (ORR), O_2 evolution (OER), and GNA oxidation (GOR) half-reactions on 20%Pt/C electrodes (0.1 M perchloric acid solutions saturated by 1 bar O_2). The measured currents (in mA) were normalized by the Pt site density to give electron conversion rates. Without added GNA, the onset potentials for ORR and OER half-reactions occur at overpotentials greater than 200 mV relative to their equilibrium potential (e.g., for ORR, 950 vs. 1170 mV vs. SHE), suggesting that these reactions are in the irreversible Butler-Volmer kinetic regime. With 0.5 M GNA, GOR half-reactions occur with at least 500 mV lower potential than OER, suggesting that the forward gluconic acid oxidation turnover rates are much faster than the reverse reaction of O_2 reduction. Together, the $^{16}O_2$-$H_2$$^{18}O$ experiments and independent electrochemical studies confirm that O_2 reduction remains irreversible during steady state catalysis on Pt surfaces.

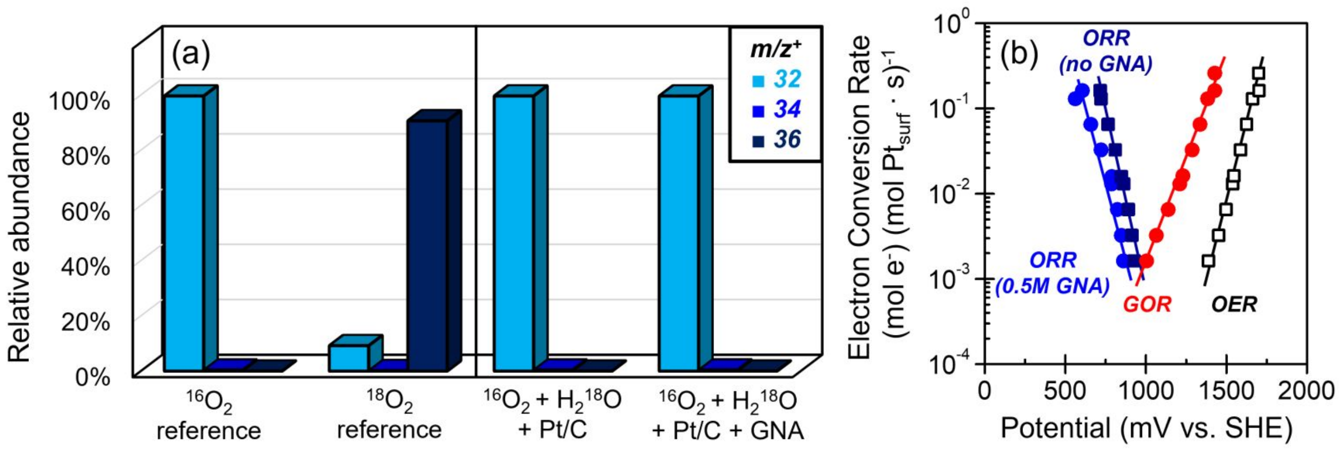

Figure S10. (a) Relative mass fragments of $^{16}O_2$, $^{16}O^{18}O$, and $^{18}O_2$ isotopologues (m/z$^+$ = 32, 34, and 36, respectively) in batch reactor headspaces during $^{16}O_2$-$H_2$$^{18}O$ isotope scrambling experiments, without or with 0.14 M GNA, alongside $^{16}O_2$ and $^{18}O_2$ references, 400 kPa O_2 (68 cm^3_{vapor}), 2 cm^3_{liquid} (300 RPM), 353 K, 0.015 g Pt/C catalyst. (b) Rates of electron consumption and generation in O_2 reduction (ORR), O_2 evolution (OER), and GNA oxidation (GOR) electrochemical half-reactions as a function of potential, derived from stepwise voltammetry profiles. 0.1 M $HClO_4$ (0.15 L, pH 1.0), 0 or 0.5 M GNA, 1 bar O_2 (600 cm^3 h^{-1} sparge), 600 RPM, 10 mg 20% Pt/C catalyst, 293 K, 2.6–3.0 Ω_s, manual iR compensation.

S11. Mass Spectra of GNA and GRA from Reactions with $^{16}O_2$ and $H_2{}^{18}O$

^{18}O can incorporate into both GNA and the GNA oxidation products during GNA-$^{16}O_2$-$H_2{}^{18}O$ batch kinetic experiments on Pt/C catalysts. Figure S11 compares the mass fragmentations of GNA and the GNA oxidation products of guluronic acid (GLA), 2- and 5-keto gluconic acid (ketos), and glucaric acid (GRA) after two-hour batch reactions at 353 K (GNA conversion approximately 15%). For GNA, GLA, and the keto-gluconic acids, the molecular weights for all molecular ion fragments shift up by 4 g mol^{-1} (195 to 199), indicating that two ^{18}O atoms replace ^{16}O in the molecule. Similarly, for GRA, the molecular ion fragment shifts up by 8 g mol^{-1} (209 to 217), indicating that four ^{18}O atoms replace ^{16}O in the molecule. We ascribe this incorporation to the homogeneous H_3O^+-catalyzed substitution of carboxylate ^{16}O-H with ^{18}O-H, analogous to a transesterification or ester hydrolysis reaction. Within the detection limits, ^{18}O does not incorporate into the other hydroxyl groups of the polyol molecules.

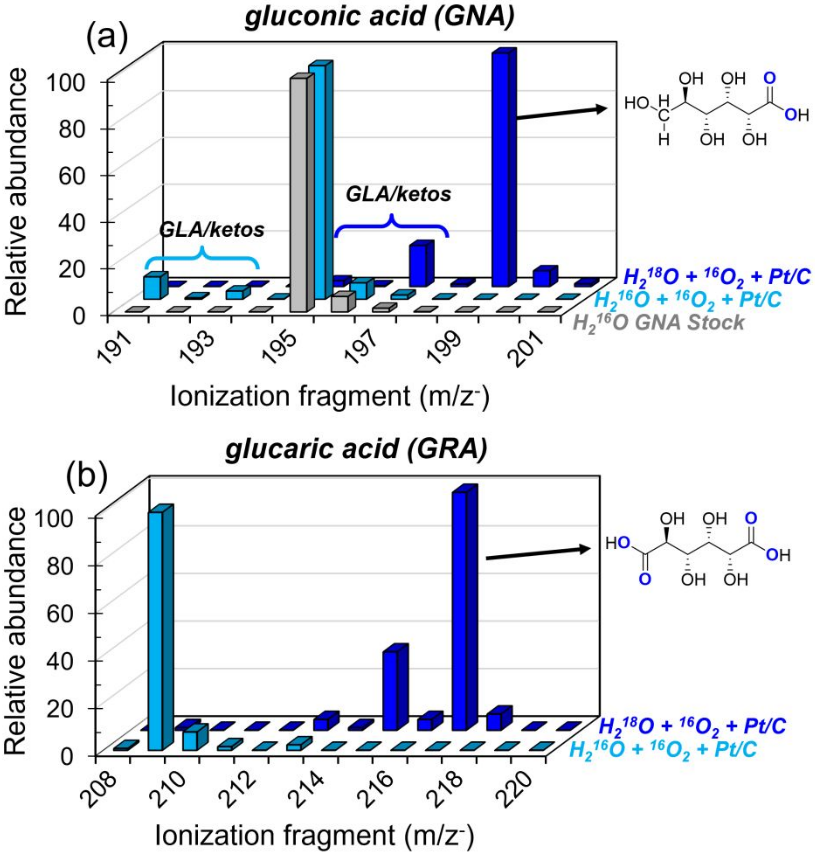

Figure S11. Mass fragment distribution of GNA and GNA-derived products (GLA, keto-gluconic acids, and GRA) in GNA-$^{16}O_2$-$H_2{}^{16}O$ (light blue) and GNA-$^{16}O_2$-$H_2{}^{18}O$ (dark blue) reactions. The fragmentations of a GNA-$H_2{}^{16}O$ stock solution are provided as reference (gray). 0.12 M GNA (2 cm^3_{liquid}, 300 RPM), 400 kPa O_2 (68 cm^3_{vapor}), 353 K, 0.015 g Pt/C catalyst.

S12. Pseudo Steady State Derivations of Rate Equations from Thermocatalytic Elementary Steps

Scheme 2 captures a sequence of elementary steps consistent with kinetic and isotopic assessments of aqueous GNA-O_2 reactions on Pt/C catalysts. These elementary steps are rewritten as follows:

O_2 Reduction Half-Reaction

O.1.	$* \ + \ O_2 \rightleftharpoons OO*$	K_{O_2}
O.2.	$OO* \ + \ H_2O \rightarrow OH_2OO*$	$k_{O.1}$
O.3.	$OH_2OO* \ + \ * \nrightarrow HO* \ + \ HOO*$	$k_{O.2}$
O.4.	$HOO* \ + \ * \rightarrow HO* \ + \ O*$	$k_{O.3}$
O.5.	$O* \ + \ H_2O + \ * \rightarrow 2\,HO*$	$k_{O.4}$

Net O_2 Reduction:

ORR.	$O_2 + 4* \ + \ 2\,H_2O \rightarrow 4\,HO*$	r_{ORR}

Alcohol Oxidation Half-Reaction

G.1.	$*_g \ + \ RCH_2OH \rightleftharpoons RCH_2OH*_g$	K_{GNA}
G.2.	$RCH_2OH*_g \ + \ HO* \nrightarrow RCHOH*_g + H_2O + \ *$	k_{C-H}
G.3.	$RCHOH*_g \ + \ HO* \rightarrow RCHO*_g + H_2O + \ *$	k_{O-H}
G.4.	$RCHO*_g \rightleftharpoons \ *_g \ + \ RCHO$	K_{GLA}^{-1}

Net Alcohol Oxidation:

GOR.	$RCH_2OH + 2\,HO* \rightarrow RCHO + 2\,H_2O + \ *$	r_{C-H}

Nomenclature:

R = $C_5H_9O_6$ for GNA; $*$: O_2 reduction active site; $*_g$: GNA binding multi-site ensemble;

$\rightleftharpoons$:Quasi equilibrated; $\rightarrow$:irreversible; $\nrightarrow$: kinetically-relevant

Derivation of a General Rate Equation

Overall turnover rates (-r_{GNA}) are defined by step G.2 with C-H scission as the kinetically relevant step:

$$\frac{-r_{GNA}}{[*_g]_T} = r_{G.2} = k_{C-H}\left(\frac{[RCH_2OH*_g]}{[*_g]_T}\right)\left(\frac{[HO*]}{[*]_T}\right) \tag{S12.1}$$

Subscript T denotes the total number of active sites. Quasi equilibrium approximation for step G.1:

$$K_{GNA} = \frac{[RCH_2OH*_g]}{[*_g]a_{GNA}};\ [RCH_2OH *_g] = K_{GNA}a_{GNA}[*_g] \tag{S12.2}$$

Substituting Eqn. S12.2 into Eqn. S12.1:

$$\frac{-r_{GNA}}{[*_g]_T} = k_{C-H}K_{GNA}a_{GNA}\left(\frac{[*_g]}{[*_g]_T}\right)\left(\frac{[HO*]}{[*]_T}\right) \tag{S12.3}$$

Pseudo steady state approximation on $[HO*]$ from an overall reaction balance:

$$\frac{d[HO*]}{dt} = 4r_{ORR} - 2r_{C-H} = 0 \tag{S12.4}$$

Oxidation half-reactions are limited by step G.2. Reduction half-reactions are limited by O=O bond scission in Step O.3, after the polarization of adsorbed O_2 (in this case, through its formation of an adduct with H_2O as OH_2OO*, but when explicitly denoting electronic charge, forms OO^-*, analogous to the elementary steps in H_2O_2 direct synthesis[6]). Combining these steps into Eqn. S12.4:

$$4\left(k_{O.2}\frac{[OH_2OO*][*]}{[*]_T^2}\right) = 2\left(k_{C-H}K_{GNA}a_{GNA}\left(\frac{[*_g]}{[*_g]_T}\right)\left(\frac{[HO*]}{[*]_T}\right)\right) \tag{S12.5}$$

Pseudo steady state balance on activated O_2 complex $[OH_2OO*]$:

$$\frac{d[OH_2OO*]}{dt} = r_{O.2} - r_{O.3} = 0 \tag{S12.6}$$

$$k_{O.2}\frac{[OH_2OO*][*]}{[*]_T^2} = k_{O.1}a_{H_2O}\frac{[OO*]}{[*]_T} \tag{S12.7}$$

$$4\left(k_{O.1}a_{H_2O}\frac{[OO*]}{[*]_T}\right) = 2\left(k_{C-H}K_{GNA}a_{GNA}\left(\frac{[*_g]}{[*_g]_T}\right)\left(\frac{[HO*]}{[*]_T}\right)\right) \tag{S12.8}$$

Quasi-equilibrium approximation on Step O.1:

$$K_{O_2} = \frac{[OO*]}{a_{O_2}[*]};\quad [OO*] = K_{O_2}a_{O_2}[*] \tag{S12.9}$$

Substituting Eqn. S12.9 into S12.8 and rearranging to isolate $[HO*]$:

$$\frac{[HO*]}{[*]_T} = \left(\frac{[*]}{[*]_T}\right)\left(\frac{[*_g]_T}{[*_g]}\right)\left(\frac{2k_{O.1}K_{O_2}a_{H_2O}}{k_{C-H}K_{GNA}}\right)\left(\frac{a_{O_2}}{a_{GNA}}\right) \tag{S12.10}$$

Substituting Eqn. S12.10 into Eqn. S12.3:

$$\frac{-r_{GNA}}{[*_g]_T} = 2k_{O.1}K_{O_2}a_{H_2O}a_{O_2}\left(\frac{[*]}{[*]_T}\right) \tag{S12.11}$$

Two coupled site balances:

$$[*]_T = [*] + [OO*] + [HO*];\qquad [*_g]_T = [*_g] + [RCH_2OH*_g] \tag{S12.12}$$

$$[\,*\,]_T = [\,*\,] + K_{O_2}a_{O_2}[\,*\,] + \left(\frac{[*_g]_T}{[*_g]}\right)\left(\frac{2k_{O.1}K_{O_2}a_{H_2O}}{k_{C-H}K_{GNA}}\right)\left(\frac{a_{O_2}}{a_{GNA}}\right)[\,*\,] \quad \text{(S12.13)}$$

$$\frac{[*_g]_T}{[*_g]} = 1 + K_{GNA}a_{GNA} \quad \text{(S12.14)}$$

Substituting Eqns. S12.2, S12.9, S12.10, and S12.14 into Eqn. S12.13:

$$\frac{[*]_T}{[*]} = 1 + K_{O_2}a_{O_2} + \frac{\left(2k_{O.1}K_{O_2}a_{H_2O}\right)a_{O_2}(1+K_{GNA}a_{GNA})}{k_{C-H}K_{GNA}a_{GNA}} \quad \text{(S12.15)}$$

Substituting Eqn. S12.15 back into Eqn. S12.11:

$$\frac{-r_{GNA}}{[*_g]_T} = \frac{\left(2k_{O.1}K_{O_2}a_{H_2O}\right)a_{O_2}}{\left(1+K_{O_2}a_{O_2}+\frac{\left(2k_{O.1}K_{O_2}a_{H_2O}\right)a_{O_2}(1+K_{GNA}a_{GNA})}{k_{C-H}K_{GNA}a_{GNA}}\right)} \quad \text{(S12.16)}$$

Further rearranging Eqn. S12.16 gives the final rate equation:

$$\frac{-r_{GNA}}{[*_g]_T} = k_{C-H}\left(\frac{K_{GNA}a_{GNA}}{1+K_{GNA}a_{GNA}}\right)\left(\frac{\left(2k_{O.1}K_{O_2}a_{H_2O}\right)a_{O_2}}{\frac{k_{C-H}K_{GNA}a_{GNA}(1+K_{O_2}a_{O_2})}{(1+K_{GNA}a_{GNA})}+\left(2k_{O.1}K_{O_2}a_{H_2O}\right)a_{O_2}}\right) \quad \text{(S12.17)}$$

As expected, Eqn. S12.17 resembles that from a Mars-van Krevelen mechanism[7] in the limiting case when K_{GNA} and K_{O_2} are much smaller than unity:

$$\frac{-r_{GNA}}{[*_g]_T} = \left(\frac{(k_{C-H}K_{GNA}a_{GNA})\left(2k_{O.1}K_{O_2}a_{H_2O}a_{O_2}\right)}{(k_{C-H}K_{GNA}a_{GNA})+\left(2k_{O.1}K_{O_2}a_{H_2O}a_{O_2}\right)}\right) \quad \text{(S12.18)}$$

We next simplify Eqn. S12.17 under the following two cases.

Limiting Case 1: O_2 Reduction and Alcohol Oxidation Occur on the Same Active Sites

In this case, $[\,*\,] = [\,*_g]$:

$$\frac{-r_{GNA}}{[L]} = 2k_{O.1}K_{O_2}a_{H_2O}a_{O_2}\left(\frac{[*]}{[L]}\right) \quad \text{(S12.19)}$$

$[L]$ denotes the total number of active sites, equal to $[\,*\,]_T$. With a single site balance:

$$[L] = [\,*\,] + [HO\,*\,] + [OO\,*\,] + [RCH_2OH\,*\,] \quad \text{(S12.20)}$$

Substituting Eqns. S12.2, S12.9, and S12.10 into Eqn. S12.20:

$$\frac{[L]}{[*]} = 1 + \left(\frac{2k_{O.1}K_{O_2}a_{H_2O}}{k_{C-H}K_{GNA}}\right)\left(\frac{a_{O_2}}{a_{GNA}}\right) + K_{O_2}a_{O_2} + K_{GNA}a_{GNA} \quad \text{(S12.21)}$$

Substituting Eqn. S12.21 into Eqn. S12.19 gives the rate expression:

$$\frac{-r_{GNA}}{[L]} = \frac{\left(2k_{O.1}K_{O_2}a_{H_2O}\right)a_{O_2}}{1+\left(\frac{2k_{O.1}K_{O_2}a_{H_2O}}{k_{C-H}K_{GNA}}\right)\left(\frac{a_{O_2}}{a_{GNA}}\right)+K_{O_2}a_{O_2}+K_{GNA}a_{GNA}} \quad \text{(S12.22)}$$

This sequence of elementary steps was chosen to represent the kinetic results, reported in Scheme 2 of the main text giving Eqn. S12.22 as Eqn. 6 of the main text.

Limiting Case 2: O_2 Reduction Half-Reactions are Quasi Equilibrated

In this case, we treat Steps O.2-O.5 as quasi equilibrated:

O.2. $$OO* + H_2O \rightleftharpoons OH_2OO* \qquad K_{O-1}$$

O.3. $$OH_2OO* + * \rightleftharpoons HO* + HOO* \qquad K_{O-2}$$

O.4. $$HOO* + * \rightleftharpoons HO* + O* \qquad K_{O-3}$$

O.5. $$O* + H_2O + * \rightleftharpoons 2\,HO* \qquad K_{O-4}$$

The product of quasi-equilibrium expressions for all elementary steps in O_2 reduction gives $[HO*]$:

$$K_{O_2}K_{O-1}K_{O-2}K_{O-3}K_{O-4} = K_{red} = \left(\frac{[OO*]}{a_{O_2}[*]}\right)\left(\frac{[OH_2OO*]}{[OO*]a_{H_2O}}\right)\left(\frac{[HO*][HOO*]}{[OH_2OO*][*]}\right)\left(\frac{[HO*][O*]}{[HOO*][*]}\right)\left(\frac{[HO*]^2}{[O*]a_{H_2O}[*]}\right) \quad \text{(S12.23)}$$

$$K_{red} = \frac{1}{\left(a_{H_2O}\right)^2\left(a_{O_2}\right)}\left(\frac{[HO*]}{[*]}\right)^4 \quad \text{(S12.24)}$$

$$[HO*] = [*]\left(K_{red}^{1/4}a_{H_2O}^{1/2}a_{O_2}^{1/4}\right) \quad \text{(S12.25)}$$

Substituting Eqn. S12.25 into Eqn. S12.3 gives the following rate expression:

$$\frac{-r_{GNA}}{[*_g]_T} = k_{C-H}\left(K_{GNA}a_{GNA}\frac{[*_g]}{[*_g]_T}\right)\left(K_{red}^{1/4}a_{H_2O}^{1/2}a_{O_2}^{1/4}\frac{[*]}{[*]_T}\right) \quad \text{(S12.26)}$$

Two coupled site balances:

$$[*_g]_T = [*_g] + [RCH_2OH*_g] = [*_g](1 + K_{GNA}a_{GNA}) \quad \text{(S12.27)}$$

$$[*]_T = [*] + [OO*] + [HO*] = [*]\left(1 + K_{O_2}a_{O_2} + K_{red}^{\frac{1}{4}}a_{H_2O}^{\frac{1}{2}}a_{O_2}^{\frac{1}{4}}\right) \quad \text{(S12.28)}$$

Substituting Eqns. S12.27 and S12.28 into Eqn. S12.26 gives the final four-parameter rate equation:

$$\frac{-r_{GNA}}{[*_g]_T} = k_{C-H}\left(\frac{K_{GNA}a_{GNA}}{1+K_{GNA}a_{GNA}}\right)\left(\frac{K_{red}^{\frac{1}{4}}a_{H_2O}^{\frac{1}{2}}a_{O_2}^{\frac{1}{4}}}{1+K_{O_2}a_{O_2}+K_{red}^{\frac{1}{4}}a_{H_2O}^{\frac{1}{2}}a_{O_2}^{\frac{1}{4}}}\right) \quad \text{(S12.29)}$$

This sequence of elementary steps could not represent the kinetic results, because a quasi-equilibrated O_2 reduction half-reaction would evolve $^{18}O_2$ during $^{16}O_2$-$H_2{}^{18}O$ experiments, inconsistent with the findings in Section S10.

Alternative Case 1: O_2 Reduction Requires Paired Active Sites

Here, Steps O.2 and O.3 occur simultaneously in a single kinetically relevant step involving concerted H_2O O-H scission, O=O scission, and Pt-OH formation:

O.2. $$OO* \ + H_2O + \ * \nrightarrow HO* \ + HOO*$$ k_{O-1}

Pseudo steady state approximation on $[HO*]$ from an overall reaction balance:

$$\frac{d[HO*]}{dt} = 4r_{red} - 2r_{ox} = 0 \quad \text{(S12.30)}$$

Oxidation half-reactions are limited by step G.2, and reduction half-reactions are limited by Step O.2:

$$4\left(k_{O.1}a_{H_2O}\frac{[OO*][*]}{[*]_T^2}\right) = 2\left(k_{C-H}K_{GNA}a_{GNA}\left(\frac{[*_g]}{[*_g]_T}\right)\left(\frac{[HO*]}{[*]_T}\right)\right) \quad \text{(S12.31)}$$

Substituting Eqn. S12.9 into Eqn. S12.31 and rearranging to isolate $[HO*]$:

$$\frac{[HO*]}{[*]_T} = \left(\frac{[*]}{[*]_T}\right)^2\left(\frac{[*_g]_T}{[*_g]}\right)\left(\frac{2k_{O.1}K_{O_2}a_{H_2O}a_{O_2}}{k_{C-H}K_{GNA}a_{GNA}}\right) \quad \text{(S12.32)}$$

Substituting Eqn. S12.32 into Eqn. S12.3:

$$\frac{-r_{GNA}}{[*_g]_T} = 2k_{O.1}K_{O_2}a_{H_2O}a_{O_2}\left(\frac{[*]}{[*]_T}\right)^2 \quad \text{(S12.33)}$$

Two coupled site balances:

$$[*]_T = [*] + [HO*] + [OO*]; \qquad [*_g]_T = [*_g] + [RCH_2OH*_g] \quad \text{(S12.34)}$$

Substituting Eqns. S12.2, S12.9, and S12.10 into Eqn. S12.34:

$$[*]_T = [*] + K_{O_2}a_{O_2}[*] + \frac{[*]^2}{[*]_T}\left(\frac{[*_g]_T}{[*_g]}\right)\left(\frac{2k_{O.1}K_{O_2}a_{H_2O}a_{O_2}}{k_{C-H}K_{GNA}a_{GNA}}\right) \quad \text{(S12.35)}$$

$$\frac{[*_g]_T}{[*_g]} = 1 + K_{GNA}a_{GNA} \quad \text{(S12.36)}$$

Substituting Eqn. S12.36 into Eqn. S12.35, then rearranging and simplifying:

$$0 = [*]^2\left(\frac{2k_{O.1}K_{O_2}a_{H_2O}a_{O_2}(1+K_{GNA}a_{GNA})}{k_{C-H}K_{GNA}a_{GNA}[*]_T}\right) + [*]\left(1 + K_{O_2}a_{O_2}\right) - [*]_T \quad \text{(S12.37)}$$

$$0 = [*]^2 + [*]\left(\frac{k_{C-H}K_{GNA}a_{GNA}[*]_T\left(1+K_{O_2}a_{O_2}\right)}{2k_{O.1}K_{O_2}a_{H_2O}a_{O_2}(1+K_{GNA}a_{GNA})}\right) - \left(\frac{k_{C-H}K_{GNA}a_{GNA}[*]_T^2}{2k_{O.1}K_{O_2}a_{H_2O}a_{O_2}(1+K_{GNA}a_{GNA})}\right) \quad \text{(S12.38)}$$

$$[*] = -\frac{1}{2}\left(\frac{k_{C-H}K_{GNA}a_{GNA}[*]_T\left(1+K_{O_2}a_{O_2}\right)}{2k_{O.1}K_{O_2}a_{H_2O}a_{O_2}(1+K_{GNA}a_{GNA})}\right) \pm \frac{1}{2}\sqrt{\left(\frac{k_{C-H}K_{GNA}a_{GNA}[*]_T\left(1+K_{O_2}a_{O_2}\right)}{2k_{O.1}K_{O_2}a_{H_2O}a_{O_2}(1+K_{GNA}a_{GNA})}\right)^2 + \frac{2k_{C-H}K_{GNA}a_{GNA}[*]_T^2}{k_{O.1}K_{O_2}a_{H_2O}a_{O_2}(1+K_{GNA}a_{GNA})}} \quad \text{(S12.39)}$$

We take the positive solution because the negative solution would give a non-physical value for coverages:

$$[\,*\,] = -\frac{1}{2}\left(\frac{k_{C-H}K_{GNA}a_{GNA}[*]_T\left(1+K_{O_2}a_{O_2}\right)}{2k_{O.1}K_{O_2}a_{H_2O}a_{O_2}(1+K_{GNA}a_{GNA})}\right) + \frac{1}{2}\sqrt{\left(\frac{k_{C-H}K_{GNA}a_{GNA}[*]_T\left(1+K_{O_2}a_{O_2}\right)}{2k_{O.1}K_{O_2}a_{H_2O}a_{O_2}(1+K_{GNA}a_{GNA})}\right)^2 + \frac{2k_{C-H}K_{GNA}a_{GNA}[*]_T^2}{k_{O.1}K_{O_2}a_{H_2O}a_{O_2}(1+K_{GNA}a_{GNA})}} \quad \text{(S12.40)}$$

Substituting Eqn. S12.40 into Eqn. S12.33:

$$\frac{-r_{GNA}}{[*]_T} = \frac{2k_{O.1}K_{O_2}a_{H_2O}a_{O_2}}{[*]_T^2}\left(-\frac{1}{2}\left(\frac{k_{C-H}K_{GNA}a_{GNA}[*]_T\left(1+K_{O_2}a_{O_2}\right)}{2k_{O.1}K_{O_2}a_{H_2O}a_{O_2}(1+K_{GNA}a_{GNA})}\right) + \frac{1}{2}\sqrt{\left(\frac{k_{C-H}K_{GNA}a_{GNA}[*]_T\left(1+K_{O_2}a_{O_2}\right)}{2k_{O.1}K_{O_2}a_{H_2O}a_{O_2}(1+K_{GNA}a_{GNA})}\right)^2 + \frac{2k_{C-H}K_{GNA}a_{GNA}[*]_T^2}{k_{O.1}K_{O_2}a_{H_2O}a_{O_2}(1+K_{GNA}a_{GNA})}}\right)^2 \quad \text{(S12.41)}$$

Simplifying:

$$\frac{-r_{GNA}}{[*]_T} = \frac{k_{O.1}K_{O_2}a_{H_2O}a_{O_2}}{2}\left(-\left(\frac{k_{C-H}K_{GNA}a_{GNA}\left(1+K_{O_2}a_{O_2}\right)}{2k_{O.1}K_{O_2}a_{H_2O}a_{O_2}(1+K_{GNA}a_{GNA})}\right) + \sqrt{\left(\frac{k_{C-H}K_{GNA}a_{GNA}\left(1+K_{O_2}a_{O_2}\right)}{2k_{O.1}K_{O_2}a_{H_2O}a_{O_2}(1+K_{GNA}a_{GNA})}\right)^2 + \frac{2k_{C-H}K_{GNA}a_{GNA}}{k_{O.1}K_{O_2}a_{H_2O}a_{O_2}(1+K_{GNA}a_{GNA})}}\right)^2 \quad \text{(S12.42)}$$

Further simplifying:

$$\frac{-r_{GNA}}{[*]_T} = \frac{k_{O.1}K_{O_2}a_{H_2O}a_{O_2}}{2}\left(\left(\frac{k_{C-H}K_{GNA}a_{GNA}\left(1+K_{O_2}a_{O_2}\right)}{2k_{O.1}K_{O_2}a_{H_2O}a_{O_2}(1+K_{GNA}a_{GNA})}\right)^2 - 2\left(\frac{k_{C-H}K_{GNA}a_{GNA}\left(1+K_{O_2}a_{O_2}\right)}{2k_{O.1}K_{O_2}a_{H_2O}a_{O_2}(1+K_{GNA}a_{GNA})}\right)\sqrt{\left(\frac{k_{C-H}K_{GNA}a_{GNA}\left(1+K_{O_2}a_{O_2}\right)}{2k_{O.1}K_{O_2}a_{H_2O}a_{O_2}(1+K_{GNA}a_{GNA})}\right)^2 + \frac{2k_{C-H}K\text{[illegible]}}{k_{O.1}K_{O_2}a_{H_2O}a_{O}\text{[illegible]}}}\text{[illegible]}\right. \quad \text{(S12.43)}$$

$$\frac{-r_{GNA}}{[*]_T} = \frac{k_{C-H}K_{GNA}a_{GNA}}{(1+K_{GNA}a_{GNA})}\left(1 + \left(1+K_{O_2}a_{O_2}\right)\left(\frac{k_{C-H}K_{GNA}a_{GNA}\left(1+K_{O_2}a_{O_2}\right)}{2K_{O_2}k_{O.1}a_{O_2}(1+K_{GNA}a_{GNA})}\right) - \left(1+K_{O_2}a_{O_2}\right)\sqrt{\left(\frac{k_{C-H}K_{GNA}a_{GNA}\left(1+K_{O_2}a_{O_2}\right)}{2K_{O_2}k_{O.1}a_{O_2}(1+K_{GNA}a_{GNA})}\right)^2 + \frac{2k_{C-H}K_{GNA}a_{GNA}}{K_{O_2}k_{O.1}a_{O_2}(1+K_{GNA}a_{GNA}\text{[illegible]}}}\text{[illegible]}\right. \quad \text{(S12.44)}$$

While Eqn. S12.44 can accurately capture all kinetic results, the added algebraic complexity does not add any additional fitting confidence, as described in Section S13.

Limiting Case 1.1: Alcohol Oxidation and O_2 Reduction Occur on the Same Active Sites

In this case, $[\,*\,] = [\,*_g]$:

$$\frac{-r_{GNA}}{[L]} = k_{C-H}K_{GNA}a_{GNA}\left(\frac{[*]}{[L]}\right)\left(\frac{[HO*]}{[L]}\right) \tag{S12.45}$$

$[L]$ denotes the total number of active sites, equal to $[\,*\,]_T$. Pseudo steady state approximation on $[HO\,*\,]$ from an overall reaction balance:

$$\frac{d[HO*]}{dt} = 4r_{red} - 2r_{ox} = 0 \tag{S12.46}$$

Oxidation half-reactions are limited by step G.2, and reduction half-reactions are limited by Step O.2:

$$4\left(k_{O.1}[OO\,*\,][\,*\,]a_{H_2O}\right) = 2\left(k_{C-H}[RCH_2OH\,*\,][HO\,*\,]\right) \tag{S12.47}$$

Substituting Eqn. S12.9 into Eqn. S12.47 and rearranging to isolate $[HO\,*\,]$:

$$[HO\,*\,] = [\,*\,]\left(\frac{2k_{O.1}K_{O_2}a_{H_2O}}{k_{C-H}K_{GNA}}\right)\left(\frac{a_{O_2}}{a_{GNA}}\right) \tag{S12.48}$$

Substituting Eqn. S12.48 into Eqn. S12.45:

$$\frac{-r_{GNA}}{[L]} = \left(2k_{O.1}K_{O_2}a_{H_2O}\right)a_{O_2}\left(\frac{[*]}{[L]}\right)^2 \tag{S12.49}$$

Site balance:

$$[L] = [\,*\,] + [OO\,*\,] + [HO\,*\,] + [RCH_2OH\,*\,] \tag{S12.50}$$

Substituting Eqns. S12.2, S12.9, and S12.48 into Eqn. S12.50:

$$\frac{[L]}{[*]} = 1 + K_{O_2}a_{O_2} + \left(\frac{2k_{O.1}K_{O_2}a_{H_2O}}{k_{C-H}K_{GNA}}\right)\left(\frac{a_{O_2}}{a_{GNA}}\right) + K_{GNA}a_{GNA} \tag{S12.51}$$

Substituting Eqn. S12.51 into Eqn. S12.49 gives the rate equation:

$$\frac{-r_{GNA}}{[L]} = \frac{\left(2k_{O.1}K_{O_2}a_{H_2O}\right)a_{O_2}}{\left(1+K_{O_2}a_{O_2}+\left(\frac{2k_{O.1}K_{O_2}a_{H_2O}}{k_{C-H}K_{GNA}}\right)\left(\frac{a_{O_2}}{a_{GNA}}\right)+K_{GNA}a_{GNA}\right)^2} \tag{S12.52}$$

Eqn. S12.52 cannot accurately capture all kinetic results, because there are no observable negative order regimes for either O_2 or GNA, and in the dilute GNA limit, rates would be second order with respect to a_{GNA} simultaneous with rates that are negative first order with respect to a_{O_2}.

Alternative Case 2: Quasi-Equilibrated O-H Scission Precedes C-H Scission

Here, the O_2 reduction half-reaction remains identical to the elementary steps of Scheme 2, but the alcohol oxidation reaction proceeds through the updated sequence of elementary steps in which Step G.2 consists of a quasi-equilibrated O-H scission to form an alkoxy surface intermediate:

Alcohol Oxidation Half-Reaction

G.1. $\quad *_g + RCH_2OH \rightleftharpoons RCH_2OH *_g \quad K_{GNA}$

G.2. $$RCH_2OH*_g + HO* \rightleftharpoons RCH_2O*_g + H_2O + * \qquad K_{O-H}$$

G.3. $$RCH_2O*_g + HO* \nrightarrow RCHO*_g + H_2O + * \qquad k_{C-H}$$

G.4. $$RCHO*_g \rightleftharpoons *_g + RCHO \qquad K_{GLA}^{-1}$$

We note that the case when Step G.2. is irreversible gives an identical rate expression to Eqn. S12.17.

Overall turnover rates are defined by Step G.3:

$$\frac{-r_{GNA}}{[*_g]_T} = r_{G.3} = k_{C-H}\left(\frac{[RCH_2O*_g]}{[*_g]_T}\right)\left(\frac{[HO*]}{[*]_T}\right) \tag{S12.53}$$

Quasi equilibrium approximation for steps G.2 and G.1:

$$K_{O-H} = \frac{[RCH_2O*_g][*]a_{H_2O}}{[RCH_2OH*_g][HO*]};\ \ [RCH_2O*_g] = \frac{K_{O-H}}{a_{H_2O}}[RCH_2OH*_g]\left(\frac{[HO*]}{[*]}\right) \tag{S12.54}$$

$$K_{GNA} = \frac{[RCH_2OH*_g]}{[*_g]a_{GNA}},\ [RCH_2OH*_g] = K_{GNA}a_{GNA}[*_g] \tag{S12.55}$$

Substituting Eqns. S12.54 and S12.55 into Eqn. S12.53:

$$\frac{-r_{GNA}}{[*_g]_T} = \frac{k_{C-H}K_{GNA}K_{O-H}}{a_{H_2O}}a_{GNA}\left(\frac{[*_g]}{[*_g]_T}\right)\left(\frac{[HO*]}{[*]}\right)\left(\frac{[HO*]}{[*]_T}\right) \tag{S12.56}$$

Pseudo steady state approximation on $[HO*]$ from an overall reaction balance:

$$\frac{d[HO*]}{dt} = 4r_{ORR} - 2r_{C-H} = 0 \tag{S12.57}$$

Oxidation half-reactions are limited by step G.3, and reduction half-reactions are limited by Step O.3:

$$4\left(k_{O.2}\frac{[OH_2OO*][*]}{[*]_T^2}\right) = 2\left(\frac{k_{C-H}K_{GNA}K_{O-H}}{a_{H_2O}}a_{GNA}\left(\frac{[*_g]}{[*_g]_T}\right)\left(\frac{[HO*]}{[*]}\right)\left(\frac{[HO*]}{[*]_T}\right)\right) \tag{S12.58}$$

Pseudo steady state balance on activated O_2 complex $[OH_2OO*]$:

$$\frac{d[OH_2OO*]}{dt} = r_{O.2} - r_{O.3} = 0 \tag{S12.59}$$

$$k_{O.2}\frac{[OH_2OO*][*]}{[*]_T^2} = k_{O.1}a_{H_2O}\frac{[OO*]}{[*]_T} \tag{S12.60}$$

Substituting Eqn. S12.60 into Eqn. S12.58:

$$4\left(k_{O.1}a_{H_2O}\frac{[OO*]}{[*]_T}\right) = 2\left(\frac{k_{C-H}K_{GNA}K_{O-H}}{a_{H_2O}}a_{GNA}\left(\frac{[*_g]}{[*_g]_T}\right)\left(\frac{[HO*]^2}{[*][*]_T}\right)\right) \tag{S12.61}$$

Substituting Eqn. S12.9 into Eqn. S12.61 and rearranging to isolate $[HO*]$:

$$\left(\frac{[HO*]^2}{[*][*]_T}\right) = \left(\frac{[*]}{[*]_T}\right)\left(\frac{[*_g]_T}{[*_g]}\right)\left(\frac{2k_{O.1}K_{O_2}a_{H_2O}^2}{k_{C-H}K_{GNA}K_{O-H}}\right)\left(\frac{a_{O_2}}{a_{GNA}}\right) \tag{S12.62}$$

$$[HO*] = [*]\sqrt{\left(\frac{[*_g]_T}{[*_g]}\right)\left(\frac{2k_{O.1}K_{O_2}a_{H_2O}^2}{k_{C-H}K_{GNA}K_{O-H}}\right)\left(\frac{a_{O_2}}{a_{GNA}}\right)} \tag{S12.63}$$

Substituting Eqn. S12.63 into Eqn. S12.56:

$$\frac{-r_{GNA}}{[*_g]_T} = \frac{k_{C-H}K_{GNA}K_{O-H}}{a_{H_2O}}a_{GNA}\left(\frac{[*_g]}{[*_g]_T}\right)\left(\frac{\left([*]\sqrt{\left(\frac{[*_g]_T}{[*_g]}\right)\left(\frac{2k_{O.1}K_{O_2}a_{H_2O}^2}{k_{C-H}K_{GNA}K_{O-H}}\right)\left(\frac{a_{O_2}}{a_{GNA}}\right)}\right)^2}{[*][*]_T}\right) \tag{S12.64}$$

$$\frac{-r_{GNA}}{[*_g]_T} = \left(2k_{O.1}K_{O_2}a_{H_2O}\right)a_{O_2}\left(\frac{[*]}{[*]_T}\right) \tag{S12.65}$$

Two coupled site balances:

$$[*]_T = [*] + [OO*] + [HO*]; \qquad [*_g]_T = [*_g] + [RCH_2OH*_g] \tag{S12.66}$$

Substituting Eqns. S12.9, S12.55, and S12.63 into Eqn. S12.66:

$$[*]_T = [*] + K_{O_2}a_{O_2}[*] + [*]\sqrt{\left(\frac{[*_g]_T}{[*_g]}\right)\left(\frac{2k_{O.1}K_{O_2}a_{H_2O}^2}{k_{C-H}K_{GNA}K_{O-H}}\right)\left(\frac{a_{O_2}}{a_{GNA}}\right)} \tag{S12.67}$$

$$\frac{[*_g]_T}{[*_g]} = 1 + K_{GNA}a_{GNA} \tag{S12.68}$$

Substituting Eqn. S12.68 into Eqn. S12.67:

$$\frac{[*]_T}{[*]} = 1 + K_{O_2}a_{O_2} + \sqrt{\left(\frac{2k_{O.1}K_{O_2}a_{H_2O}^2}{k_{C-H}K_{GNA}K_{O-H}}\right)\left(\frac{a_{O_2}(1+K_{GNA}a_{GNA})}{a_{GNA}}\right)} \tag{S12.69}$$

Substituting Eqn. S12.69 into Eqn. S12.65:

$$\frac{-r_{GNA}}{[*_g]_T} = \frac{\left(2k_{O.1}K_{O_2}a_{H_2O}\right)a_{O_2}}{\left(1+K_{O_2}a_{O_2}+\sqrt{\left(\frac{2k_{O.1}K_{O_2}a_{H_2O}^2}{k_{C-H}K_{GNA}K_{O-H}}\right)\left(\frac{a_{O_2}\left(1+K_{GNA}a_{GNA}\right)}{a_{GNA}}\right)}\right)} \tag{S12.70}$$

This sequence of elementary steps could still represent all kinetic results but differs from Scheme 2 in the form of the surface alcohol species (i.e., whether C-H scission occurs on a bound alcohol or an alkoxy). This rate expression could be mechanistically distinguished from Eqn. 6 of the main text in the dilute GNA limit: in Eqn. S12.70, the maximum GNA reaction order is 0.5, versus in Eqn. 6 of the main text, which can equal 1.0. However, our current kinetic dataset cannot distinguish between the two microkinetic models.

Limiting Case 2.1: Alcohol Oxidation and O_2 Reduction Occur on the Same Active Sites

In the case where $[*] = [*_g]$:

$$\frac{-r_{GNA}}{[L]} = \frac{k_{C-H}K_{GNA}K_{O-H}}{a_{H_2O}} a_{GNA} \left(\frac{[HO*]}{[L]}\right)^2 \quad \text{(S12.71)}$$

$[L]$ denotes the total number of active sites, equal to $[*]_T$. Substituting Eqn. S12.63 into Eqn. S12.71:

$$\frac{-r_{GNA}}{[L]} = \left(2k_{O.1}K_{O_2}a_{H_2O}\right)a_{O_2}\left(\frac{[*]}{[L]}\right) \quad \text{(S12.72)}$$

Single site balance:

$$[L] = [*] + [OO*] + [HO*] + [RCH_2OH*] \quad \text{(S12.73)}$$

Substituting Eqns. S12.9, S12.55, and S12.63 into Eqn. S12.73:

$$[L] = [*] + K_{O_2}a_{O_2}[*] + \sqrt{[*][L]\left(\frac{2k_{O.1}K_{O_2}a_{H_2O}^2}{k_{C-H}K_{GNA}K_{O-H}}\right)\left(\frac{a_{O_2}}{a_{GNA}}\right)} + K_{GNA}a_{GNA}[*] \quad \text{(S12.74)}$$

Define $[A] = \sqrt{[*]}$:

$$0 = \left(1 + K_{O_2}a_{O_2} + K_{GNA}a_{GNA}\right)[A]^2 + \sqrt{[L]\left(\frac{2k_{O.1}K_{O_2}a_{H_2O}^2}{k_{C-H}K_{GNA}K_{O-H}}\right)\left(\frac{a_{O_2}}{a_{GNA}}\right)}[A] - [L] \quad \text{(S12.75)}$$

Rearranging:

$$0 = [A]^2 + \sqrt{[L]\left(\frac{2k_{O.1}K_{O_2}a_{H_2O}^2}{k_{C-H}K_{GNA}K_{O-H}}\right)\left(\frac{a_{O_2}}{a_{GNA}\left(1+K_{O_2}a_{O_2}+K_{GNA}a_{GNA}\right)^2}\right)}[A] - \frac{[L]}{\left(1+K_{O_2}a_{O_2}+K_{GNA}a_{GNA}\right)} \quad \text{(S12.76)}$$

$$[A] = -\frac{1}{2}\sqrt{[L]\left(\frac{2k_{O.1}K_{O_2}a_{H_2O}^2}{k_{C-H}K_{GNA}K_{O-H}}\right)\left(\frac{a_{O_2}}{a_{GNA}\left(1+K_{O_2}a_{O_2}+K_{GNA}a_{GNA}\right)^2}\right)} \pm \frac{1}{2}$$
$$\sqrt{[L]\left(\frac{2k_{O.1}K_{O_2}a_{H_2O}^2}{k_{C-H}K_{GNA}K_{O-H}}\right)\left(\frac{a_{O_2}}{a_{GNA}\left(1+K_{O_2}a_{O_2}+K_{GNA}a_{GNA}\right)^2}\right) + 4\frac{[L]}{\left(1+K_{O_2}a_{O_2}+K_{GNA}a_{GNA}\right)}} \quad \text{(S12.77)}$$

We take the positive root of Eqn. S12.77 to avoid negative coverages, then substitute Eqn. S12.77 into Eqn. S12.72:

$$\frac{-r_{GNA}}{[L]} = \left(2k_{O.1}K_{O_2}a_{H_2O}\right)a_{O_2}$$
$$\left(-\frac{1}{2}\sqrt{[L]\left(\frac{2k_{O.1}K_{O_2}a_{H_2O}^2}{k_{C-H}K_{GNA}K_{O-H}}\right)\left(\frac{a_{O_2}}{a_{GNA}\left(1+K_{O_2}a_{O_2}+K_{GNA}a_{GNA}\right)^2}\right)} + \frac{1}{2}\sqrt{[L]\left(\frac{2k_{O.1}K_{O_2}a_{H_2O}^2}{k_{C-H}K_{GNA}K_{O-H}}\right)\left(\frac{a_{O_2}}{a_{GNA}\left(1+K_{O_2}a_{O_2}+K_{GNA}a_{GNA}\right)^2}\right) + 4\,\cdots}\right.$$

[illegible]

(S12.78)

Simplifying Eqn. S12.78:

$$\frac{-r_{GNA}}{[L]} = \frac{\left(k_{O.1}K_{O_2}a_{H_2O}\right)a_{O_2}[L]}{2}$$

$$\left(-\sqrt{\left(\frac{2k_{O.1}K_{O_2}a_{H_2O}^2}{k_{C-H}K_{GNA}K_{O-H}}\right)\left(\frac{a_{O_2}}{a_{GNA}\left(1+K_{O_2}a_{O_2}+K_{GNA}a_{GNA}\right)^2}\right)} + \sqrt{\left(\frac{2k_{O.1}K_{O_2}a_{H_2O}^2}{k_{C-H}K_{GNA}K_{O-H}}\right)\left(\frac{a_{O_2}}{a_{GNA}\left(1+K_{O_2}a_{O_2}+K_{GNA}a_{GNA}\right)^2}\right) + \frac{4}{\left(1+K_{O_2}a_{O_2}+K\right.} \text{[illegible]}}\right.$$

(S12.79)

This sequence of elementary steps cannot describe the experimental kinetic results, because Eqn. S12.79 cannot give a positive order dependence for GNA.

Alternative Case 3: C-H Scission Forms a Metal Hydride

Here, we consider the case proposed in previous studies in which alcohol adsorption and C-H scission may form a surface hydride ($H*$), which is then oxidized by surface HO $*$.[8] The O_2 reduction half-reaction remains the same as Scheme 2, but the alcohol oxidation reaction proceeds through the updated sequence of elementary steps.

Alcohol Oxidation Half-Reaction

G.1.	$* + RCH_2OH \rightleftharpoons RCH_2OH*$	K_{GNA}
G.2.	$RCH_2OH* + * \rightarrow RCH_2O* + H*$	k_{O-H}
G.3.	$RCH_2O* + * \nrightarrow RCHO* + H*$	k_{C-H}
G.4.	$RCHO* \rightleftharpoons * + RCHO$	K_{GLA}^{-1}
G.5.	$H* + HO* \rightarrow 2* + H_2O$	k_{H-ox}

For simplicity, we consider the limiting case when alcohol oxidation and O_2 reduction occur on the same active sites.

Overall turnover rates are defined by Step G.3:

$$\frac{-r_{GNA}}{[L]} = r_{G.3} = k_{C-H}\left(\frac{[RCH_2O*]}{[L]}\right)\left(\frac{[*]}{[L]}\right) \quad \text{(S12.80)}$$

$[L]$ denotes the total number of active sites. Pseudo steady state approximation on $[RCH_2O*]$:

$$\frac{d[RCH_2O*]}{dt} = 0 = r_{G.2} - r_{G.3} \quad \text{(S12.81)}$$

$$\frac{-r_{GNA}}{[L]} = r_{G.2} = k_{O-H}\left(\frac{[RCH_2OH*]}{[L]}\right)\left(\frac{[*]}{[L]}\right) \quad \text{(S12.82)}$$

Quasi equilibrium approximation for steps G.1:

$$K_{GNA} = \frac{[RCH_2OH*]}{[*]a_{GNA}},\ [RCH_2OH*] = K_{GNA}a_{GNA}[*] \quad \text{(S12.83)}$$

Substituting Eqn. S12.83 into Eqn. S12.82:

$$\frac{-r_{GNA}}{[L]} = k_{O-H}K_{GNA}a_{GNA}\left(\frac{[*]}{[L]}\right)^2 \tag{S12.84}$$

Overall site balance:

$$[L] = [\,*\,] + [HO\,*\,] + [OO\,*\,] + [RCH_2OH\,*\,] + [H\,*\,] \tag{S12.85}$$

Pseudo steady state approximation on $[HO\,*\,]$:

$$\frac{d[HO*]}{dt} = 4r_{ORR} - r_{G.5} = 0 \tag{S12.86}$$

O_2 reduction half-reactions are limited by Step O.3:

$$4\left(k_{O.2}\,\frac{[OH_2OO*][*]}{[L]^2}\right) = k_{H-ox}\left(\frac{[H*]}{[L]}\right)\left(\frac{[HO*]}{[L]}\right) \tag{S12.87}$$

Pseudo steady state balance on activated O_2 complex $[OH_2OO\,*\,]$:

$$\frac{d[OH_2OO*]}{dt} = r_{O.2} - r_{O.3} = 0 \tag{S12.88}$$

$$k_{O.2}\frac{[OH_2OO*][*]}{[L]^2} = k_{O.1}a_{H_2O}\frac{[OO*]}{[L]} \tag{S12.89}$$

Substituting Eqn. S12.89 into Eqn. S12.87:

$$4\left(k_{O.1}a_{H_2O}\,\frac{[OO*]}{[L]}\right) = \left(k_{H-ox}\left(\frac{[H*]}{[L]}\right)\left(\frac{[HO*]}{[L]}\right)\right) \tag{S12.90}$$

Quasi-equilibrium approximation on Step O.1:

$$K_{O_2} = \frac{[OO*]}{a_{O_2}[*]};\quad [OO\,*\,] = K_{O_2}a_{O_2}[\,*\,] \tag{S12.91}$$

Substituting Eqn. S12.91 into Eqn. S12.90 and rearranging to isolate $[HO\,*\,]$:

$$4\left(k_{O.1}a_{H_2O}K_{O_2}a_{O_2}\,\frac{[*]}{[L]}\right) = \left(k_{H-ox}\left(\frac{[H*]}{[L]}\right)\left(\frac{[HO*]}{[L]}\right)\right) \tag{S12.92}$$

$$\left(\frac{[HO*]}{[L]}\right) = \frac{4k_{O.1}a_{H_2O}K_{O_2}a_{O_2}}{k_{H-ox}}\left(\frac{[*]}{[H*]}\right) \tag{S12.93}$$

Pseudo steady state balance on $[H\,*\,]$:

$$\frac{d[H*]}{dt} = 0 = r_{G.2} + r_{G.3} - r_{G.5} = 2r_{G.2} - r_{G.5} \tag{S12.94}$$

$$k_{O-H}K_{GNA}a_{GNA}\left(\frac{[*]}{[L]}\right)^2 = k_{H-ox}\left(\frac{[H*]}{[L]}\right)\left(\frac{[HO*]}{[L]}\right) \tag{S12.95}$$

Eqns. S12.92 and S12.95 are coupled, and a pseudo steady state balance cannot isolate $[H\,*\,]$ and $[HO\,*\,]$ coverages. Without an expression for either coverage, we cannot derive an analytical expression of the rate equation in which rates have a positive order dependence on O_2 pressure, and therefore we cannot describe the kinetic results. The final expression, excluding $[H\,*\,]$ and $[HO\,*\,]$ in Eqn. S12.85, is:

$$\frac{-r_{GNA}}{[L]} = \frac{k_{O-H} K_{GNA} a_{GNA}}{\left(1 + K_{O_2} a_{O_2} + K_{GNA} a_{GNA}\right)^2} \tag{S12.96}$$

S13. Linear Regression and Fitting of Microkinetic Models for GNA Oxidation

Seven model equations for the GNA consumption turnover rate (-r_{GNA}), derived from various sequences of elementary steps in Section S12 and displayed in the same order as subsections in Section S12, are as follows. All seven models contain four fitting constants, colored accordingly: O_2 activation, O_2 adsorption, alcohol activation, and alcohol adsorption.

Model 1: Irreversible GNA and O_2 activation on independent sites:

(1) $$\frac{-r_{GNA}}{[*_g]_T} = k_{C-H}\left(\frac{K_{GNA}a_{GNA}}{1+K_{GNA}a_{GNA}}\right)\left(\frac{2k_{O.1}K_{O_2}a_{O_2}}{\frac{k_{C-H}K_{GNA}a_{GNA}(1+K_{O_2}a_{O_2})}{(1+K_{GNA}a_{GNA})}+2k_{O.1}K_{O_2}a_{O_2}}\right)$$ (S13.1 = S12.17)

Model 2: Irreversible GNA and O_2 activation on the same site:

(2) $$\frac{-r_{GNA}}{[L]} = \frac{2k_{O.1}K_{O_2}a_{O_2}}{1+\left(\frac{2k_{O.1}K_{O_2}a_{O_2}}{k_{C-H}K_{GNA}a_{GNA}}\right)+K_{O_2}a_{O_2}+K_{GNA}a_{GNA}}$$ (S13.2 = S12.22)

Model 3: Quasi-equilibrated O_2 activation, GNA and O_2 activation on independent sites:

(3) $$\frac{-r_{GNA}}{[*_g]_T} = k_{C-H}\left(\frac{K_{GNA}a_{GNA}}{1+K_{GNA}a_{GNA}}\right)\left(\frac{\left(K_{red}a_{O_2}\right)^{\frac{1}{4}}}{1+K_{O_2}a_{O_2}+\left(K_{red}a_{O_2}\right)^{\frac{1}{4}}}\right)$$ (S13.3 = S12.29)

Model 4: Irreversible GNA and O_2 activation on independent sites, O_2 activation on two sites:

(4) $$\frac{-r_{GNA}}{[*]_T} = \frac{k_{C-H}K_{GNA}a_{GNA}}{(1+K_{GNA}a_{GNA})}$$
$$\left(1+\left(1+K_{O_2}a_{O_2}\right)\left(\frac{k_{C-H}K_{GNA}a_{GNA}\left(1+K_{O_2}a_{O_2}\right)}{2K_{O_2}k_{O.1}a_{O_2}(1+K_{GNA}a_{GNA})}\right)-\left(1+K_{O_2}a_{O_2}\right)\sqrt{\left(\frac{k_{C-H}K_{GNA}a_{GNA}\left(1+K_{O_2}a_{O_2}\right)}{2K_{O_2}k_{O.1}a_{O_2}(1+K_{GNA}a_{GNA})}\right)^2+\frac{2k_{C-H}K_{GNA}\text{[illegible]}}{K_{O_2}k_{O.1}a_{O_2}(1+\text{[illegible]}}}\right.$$

(S13.4 = S12.44)

Model 5: Irreversible GNA and O_2 activation on the same site, O_2 activation on two sites:

(5) $$\frac{-r_{GNA}}{[L]} = \frac{2k_{O.1}K_{O_2}a_{O_2}}{\left(1+K_{O_2}a_{O_2}+\left(\frac{2k_{O.1}K_{O_2}a_{O_2}}{k_{C-H}K_{GNA}a_{GNA}}\right)+K_{GNA}a_{GNA}\right)^2}$$ (S13.5 = S12.52)

Model 6: Irreversible GNA and O_2 activation on independent sites, quasi-equilibrated O-H scission before GNA activation:

(6) $$\frac{-r_{GNA}}{[*_g]_T} = \frac{2k_{O.1}K_{O_2}a_{O_2}}{\left(1+K_{O_2}a_{O_2}+\sqrt{\frac{2k_{O.1}K_{O_2}a_{O_2}(1+K_{GNA}a_{GNA})}{k_{C-H}K_{O-H}K_{GNA}a_{GNA}}}\right)}$$ (S13.6 = S12.70)

Model 7: Irreversible GNA and O_2 activation on the same sites, quasi-equilibrated O-H scission before GNA activation:

(7) $$\frac{-r_{GNA}}{[L]} = \frac{k_{O.1}K_{O_2}a_{O_2}[L]}{2}$$

$$\left(-\sqrt{\left(\frac{2k_{O.1}K_{O_2}a_{O_2}}{k_{C-H}K_{O-H}K_{GNA}a_{GNA}(1+K_{O_2}a_{O_2}+K_{GNA}a_{GNA})^2}\right)}+\sqrt{\left(\frac{2k_{O.1}K_{O_2}a_{O_2}}{k_{C-H}K_{O-H}K_{GNA}a_{GNA}(1+K_{O_2}a_{O_2}+K_{GNA}a_{GNA})^2}\right)+\frac{4}{(1+K_{O_2}a_{O_2}+\text{[illegible]}}}\right.$$

(S13.7 = S12.79)

We take the thermodynamic activity of water as unity in all cases. In the case of Eqn. S13.3, O_2 activation is an equilibrated process. Regression of Eqns. S13.1–S13.7 against the kinetic dependencies in Figure 2 of the main text gives the numerical fitting values of these four constants, as well as their mean regression errors, displayed in Table S1. Parity plots between measured -r_{GNA} and each fitted model are provided in Figure S12.

First, we eliminate the elementary steps comprising Eqns. S13.3, S13.5, and S13.7 as potential models, because these processes cannot accurately reproduce the kinetic dependencies of Figure 2. Those steps comprising Eqn. S13.3 require a quasi-equilibrated O_2 reduction half-reaction, and would evolve $^{18}O_2$ during $^{16}O_2$-$H_2{}^{18}O$ experiments, however, this expectation does not agree with product characterization by mass spectrometry (Section S10). Eqn. S13.5 predicts a second order a_{GNA} dependence and a negative first order a_{O_2} dependence, which does not align with the observed partial positive reaction orders with respect to both reactants. Eqn. S13.7 predicts a zero to negative order dependences on a_{GNA}, which also appears inconsistent with the observed positive order dependence on GNA concentration.

Second, the elementary steps comprising Eqns. S13.1, S13.2, S13.4, and S13.6 agree with all kinetic results, and the quality of fit for all four models compare closely in mean regression errors. Of these four models, Eqn. S13.2 presents the simplest interpretation and yields physically intuitive values for the fitting constants in Table S1. As shown in Section S14, Eqn. S13.2 can accurately capture the observed dependence on E_{cat} in Eqn. 7 of the main text, where E_{cat} scales logarithmically with the O_2 to GNA ratio,

independent of site coverage. In contrast, Eqns. S13.1 or S13.4 cannot capture this unified dependence when the two half-reactions occur on independent sites, because the coverage terms would not cancel from the expressions (*vide infra,* Eqn. S14.33). Therefore, E_{cat} would depend nonlinearly on a_{GNA} and a_{O_2}. Finally, we cannot eliminate Eqn. S13.6 as a potential model for all observed kinetics. The models of Eqns. S13.2 and S13.6 only differ in their sequence of O-H and kinetically relevant C-H bond dissociation steps. For simplicity and based on the minute differences in the mean regression error, we select the elementary steps that yield Eqn. S13.2 to accurately describe the kinetic results and coverages of reactive surface species.

Table S1. Kinetic and thermodynamic parameters from microkinetic modeling of GNA-O_2 reactions on Pt/C at 353 K.

Model Eqn. #	O_2 activation	O_2 adsorption	alcohol activation	alcohol adsorption	Mean regression error
(1)	4.4×10^{-3} s^{-1}	3.1×10^{-2} kPa^{-1}	7.2×10^{-2} s^{-1}	3.3×10^{0} L mol^{-1}	13%
(2)	4.0×10^{-3} s^{-1}	4.3×10^{-2} kPa^{-1}	2.4×10^{-1} s^{-1}	9.4×10^{-1} L mol^{-1}	14%
(3)	1.0×10^{-2} kPa^{-1}	0	1.2×10^{-2} s^{-1}	3.4×10^{1} L mol^{-1}	11%
(4)	4.9×10^{1} s^{-1}	4.0×10^{-5} kPa^{-1}	8.4×10^{-3} s^{-1}	3.0×10^{1} L mol^{-1}	12%
(5)	2.0×10^{-2} s^{-1}	1.6×10^{-3} kPa^{-1}	1.4×10^{2} s^{-1}	9.9×10^{-3} L mol^{-1}	24%
(6)	2.9×10^{-3} s^{-1}	1.9×10^{0} kPa^{-1}	2.9×10^{-1} s^{-1}	4.8×10^{-3} L mol^{-1}	19%
(7)	4.9×10^{-3} s^{-1}	3.2×10^{-2} kPa^{-1}	4.5×10^{0} s^{-1}	7.5×10^{-2} L mol^{-1}	13%

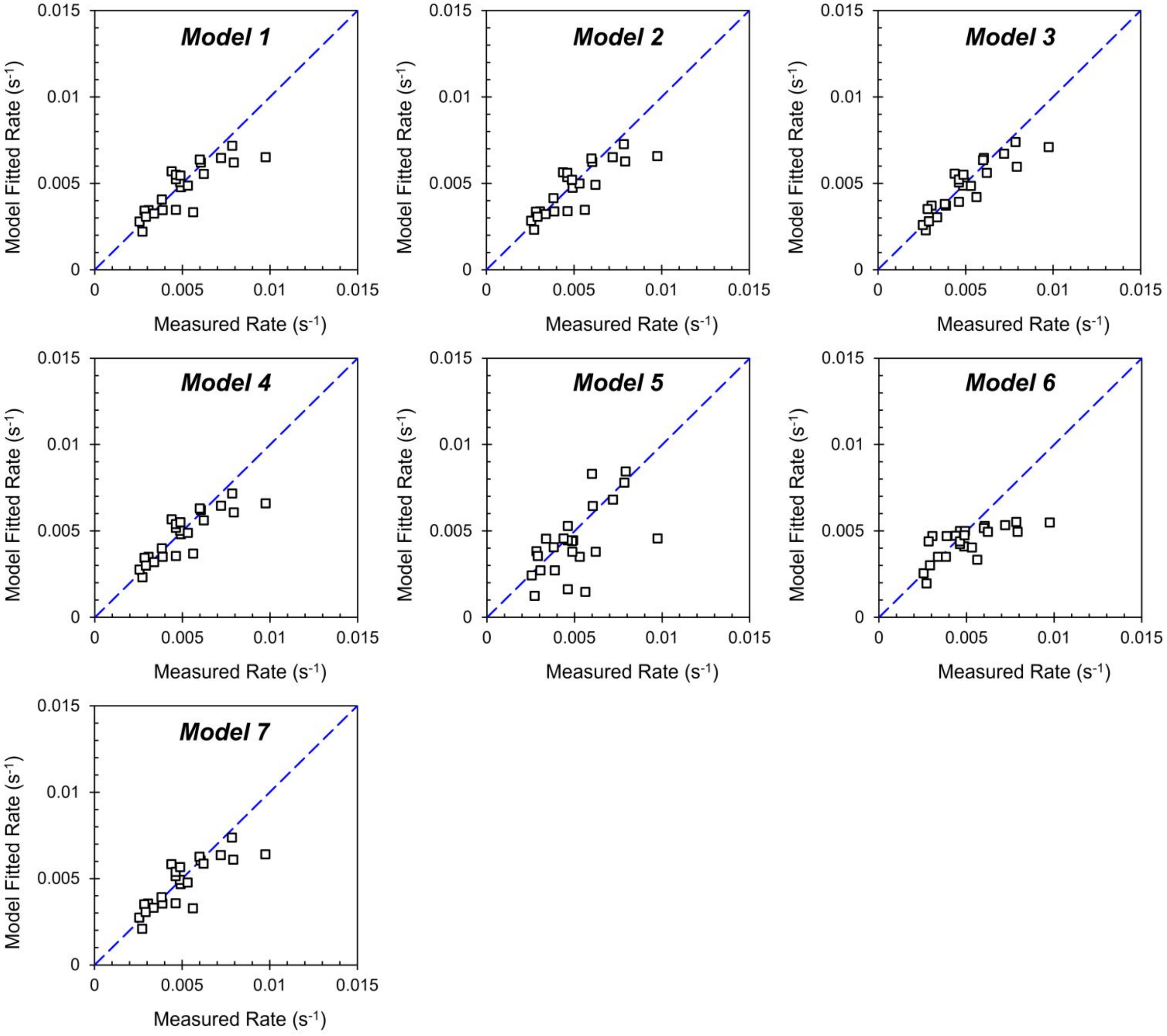

Figure S12. Parity plots between the measured GNA oxidation rates ($-r_{GNA}$) at 353 K from Figure 2 of the main text and each of the seven microkinetic model derived rate expressions (Eqns. S13.1–S13.7).

S14. Pseudo Steady State Derivations of Rate Equations and Catalyst Potential Expressions from the Electrocatalytic Analogues of Thermocatalytic Elementary Steps

Scheme 2 captures a sequence of elementary steps consistent with kinetic and isotopic assessments of aqueous GNA-O_2 reactions on Pt/C catalysts. To describe their electrocatalytic analogues, we make the following notes:

1. The elementary steps below are in the same sequence as the thermochemical analysis in Section S12, involving the same active sites, and as shown in this analysis, will result in the same functional form of the rate expression.
2. We derive the general rate expression when O_2 reduction and alcohol oxidation occur on different sites, then relax that assumption to the same site to match Scheme 2.
3. We assume all electron transfer steps are irreversible (i.e., not accounting for reverse reactions in the Butler-Volmer equation) or equilibrated (i.e., described by the Nernst equation), that rate and equilibrium constants are independent of pH, and that the electrosorption valency in adsorption steps are near zero.
4. In both half-reactions, the positive or negative polarization of adsorbates is treated explicitly as a complete and irreversible charge transfer step immediately after the chemical adsorption step, as the surrogate of an electrosorption valency.

O_2 Reduction Half-Reaction

O.1. $$* + O_2 \rightleftharpoons OO* \qquad K_{O_2}$$

O.2. $$OO* + e^- \rightarrow OO^-* \qquad k_{O-1}$$

O.3. $$OO^-* + H_2O + * \rightarrow HO^-* + HOO* \qquad k_{O-2}$$

O.4a. $$HOO* + e^- \rightarrow HOO^-* \qquad k_{O-3a}$$

O.4b. $$HOO^-* + * \rightarrow HO^-* + O* \qquad k_{O-3b}$$

O.5a. $$O* + e^- \rightarrow O^-* \qquad k_{O-4a}$$

O.5b. $$O^-* + H_2O + * \rightarrow HO^-* + HO* \qquad k_{O-4b}$$

O.5c. $$HO* + e^- \rightarrow HO^-* \qquad k_{O-4c}$$

Net O_2 reduction half-reaction:

ORR $$O_2 + 4e^- + 4* + 2H_2O \rightarrow 4\ HO^-*$$

Alcohol Oxidation Half-Reaction

G.1. $*_g + RCH_2OH \rightleftharpoons RCH_2OH *_g$ K_{GNA}

G.2a. $RCH_2OH *_g \rightarrow RCH_2OH^+ *_g + e^-$ $k_{C-H,a}$

G.2b. $RCH_2OH^+ *_g + HO^- * \nrightarrow RCHOH *_g + H_2O + *$ $k_{C-H,b}$

G.3a. $RCHOH *_g \rightarrow RCHOH^+ *_g + e^-$ $k_{O-H,a}$

G.3b. $RCHOH^+ *_g + HO^- * \rightarrow RCHO *_g + H_2O + *$ $k_{O-H,b}$

G.4. $RCHO *_g \rightleftharpoons *_g + RCHO$ K_{GLA}^{-1}

Net alcohol oxidation half-reaction:

GOR $RCH_2OH + 2\ HO^- * \rightarrow RCHO + 2\,e^- + 2\,H_2O + 2\,*$

Net thermochemical reaction:

$$2\,RCH_2OH + O_2 \rightarrow 2\,RCHO + 2\,H_2O$$

Nomenclature:

R = $C_5H_9O_6$ for GNA; $*$: O_2 reduction active site; $*_g$: GNA binding multi-site ensemble;

$\rightleftharpoons$:Quasi equilibrated; $\rightarrow$:irreversible; $\nrightarrow$: kinetically-relevant

Derivation of a General Rate Equation

We derive the rate equation of GNA oxidation (*-r_{GNA}*) starting from the kinetically relevant step of C-H bond scission, step G.2b:

$$\frac{-r_{GNA}}{[*_g]_T} = r_{G.2b} = k_{C-H,b}\left(\frac{[RCH_2OH^+*_g]}{[*_g]_T}\right)\left(\frac{[HO^-*]}{[*]_T}\right) \quad \text{(S14.1)}$$

Pseudo steady state balance on RCH_2OH^+:

$$\frac{d[RCH_2OH^+]}{dt} = 0 = r_{G.2a} - r_{G.2b} \quad \text{(S14.2)}$$

$$\frac{-r_{GNA}}{[*_g]_T} = r_{G.2a} = k^0_{C-H,a}\left(\frac{[RCH_2OH*_g]}{[*_g]_T}\right)e^{\frac{\beta_{C-H}F(E_{cat}-E^0_{C-H,a})}{RT}} \quad \text{(S14.3)}$$

To simplify irreversible Butler-Volmer kinetic expressions (e.g., Eqn. S14.3), we group the equilibrium potential E^0 into the rate constant (with analogous expressions for all subsequent electron transfer steps):

$$k^{0\prime}_{C-H} = k^0_{C-H,a}e^{\frac{-\beta_{C-H}FE^0_{C-H,a}}{RT}} \quad \text{(S14.4)}$$

$$\frac{-r_{GNA}}{[*_g]_T} = k^{0\prime}_{C-H}\left(\frac{[RCH_2OH*_g]}{[*_g]_T}\right)e^{\frac{\beta_{C-H}FE_{cat}}{RT}} \tag{S14.5}$$

Quasi equilibrium approximation for step G.1:

$$K_{GNA} = \frac{[RCH_2OH*_g]}{[*_g]a_{GNA}},\ [RCH_2OH*_g] = K_{GNA}a_{GNA}[*_g] \tag{S14.6}$$

Substituting Eqn. S14.6 into Eqn. S14.5:

$$\frac{-r_{GNA}}{[*_g]_T} = k^{0\prime}_{C-H}K_{GNA}a_{GNA}\left(\frac{[*_g]}{[*_g]_T}\right)e^{\frac{\beta_{C-H}FE_{cat}}{RT}} \tag{S14.7}$$

Complete Electron Balance

Since potential is not an independent variable, rather an emergent property of the reaction system, we use mixed potential theory to balance the electrons and remove the rate dependence on E_{cat}. At open circuit:

$$\sum i_{red} + \sum i_{ox} = i_{net} = 0 \tag{S14.8}$$

$$-\sum i_{red} = \sum i_{ox} \tag{S14.9}$$

The rate expressions for elementary steps that contribute to potential (assuming no charged adsorption and assuming all irreversible) are:

Reduction rates:

$$\frac{r_{O.2}}{[*]_T} = k^{0\prime}_{O-1}\frac{[OO*]}{[*]_T}e^{\frac{-\beta_{O-1}FE_{cat}}{RT}} \tag{S14.10}$$

$$\frac{r_{O.4a}}{[*]_T} = k^{0\prime}_{O-3}\frac{[HOO*]}{[*]_T}e^{\frac{-\beta_{O-3}FE_{cat}}{RT}} \tag{S14.11}$$

$$\frac{r_{O.5a}}{[*]_T} = k^{0\prime}_{O-4}\frac{[O*]}{[*]_T}e^{\frac{-\beta_{O-4}FE_{cat}}{RT}} \tag{S14.12}$$

$$\frac{r_{O.5c}}{[*]_T} = k^{0\prime}_{O-5}\frac{[HO*]}{[*]_T}e^{\frac{-\beta_{O-5}FE_{cat}}{RT}} \tag{S14.13}$$

Oxidation rates:

$$\frac{r_{G.2a}}{[*_g]_T} = k^{0\prime}_{C-H}\frac{[RCH_2OH*_g]}{[*_g]_T}e^{\frac{\beta_{C-H}FE_{cat}}{RT}} \tag{S14.14}$$

$$\frac{r_{G.3a}}{[*_g]_T} = k^{0\prime}_{O-H}\frac{[RCHOH*_g]}{[*_g]_T}e^{\frac{\beta_{O-H}FE_{cat}}{RT}} \tag{S14.15}$$

Since all steps above are single electron transfers, Eqn. S14.9 is written as:

$$r_{O.2} + r_{O.4a} + r_{O.5a} + r_{O.5c} = r_{G.2a} + r_{G.3a} \tag{S14.16}$$

Substituting Eqns. S14.10–15 into Eqn. S14.16:

$$\left(k_{O-1}^{0\prime}[OO*]e^{\frac{-\beta_{O-1}FE_{cat}}{RT}} + k_{O-3}^{0\prime}[HOO*]e^{\frac{-\beta_{O-3}FE_{cat}}{RT}} + k_{O-4}^{0\prime}[O*]e^{\frac{-\beta_{O-4}FE_{cat}}{RT}} + k_{O-5}^{0\prime}[HO*]e^{\frac{-\beta_{O-5}FE_{cat}}{RT}}\right) = \left(\frac{[*_g]_T}{[*]_T}\right)\left(k_{C-H}^{0\prime}[RCH_2OH*_g]e^{\frac{\beta_{C-H}FE_{cat}}{RT}} + k_{O-H}^{0\prime}[RCHOH*_g]e^{\frac{\beta_{O-H}FE_{cat}}{RT}}\right) \quad \text{(S14.17)}$$

To simplify Eqn. S14.17, we use a pseudo steady state analysis (and quasi-equilibrium when appropriate) for all intermediates.

Pseudo steady state approximation on $[HOO*]$:

$$\frac{d[HOO*]}{dt} = r_{O.3} - r_{O.4a} = 0; \quad r_{O.3} = r_{O.4a} \quad \text{(S14.18)}$$

Pseudo steady state approximation on $[OO^-*]$:

$$\frac{d[OO^-*]}{dt} = r_{O.2} - r_{O.3} = 0; \quad r_{O.2} = r_{O.3} = r_{O.4a} \quad \text{(S14.19)}$$

Pseudo steady state approximation on $[O*]$:

$$\frac{d[O*]}{dt} = r_{O.4b} - r_{O.5a} = 0; \quad r_{O.4b} = r_{O.5a} \quad \text{(S14.20)}$$

Pseudo steady state approximation on $[HOO^-*]$:

$$\frac{d[HOO^-*]}{dt} = r_{O.4a} - r_{O.4b} = 0; \quad r_{O.4a} = r_{O.4b} = r_{O.5a} = r_{O.2} \quad \text{(S14.21)}$$

Pseudo steady state approximation on $[HO*]$:

$$\frac{d[HO*]}{dt} = r_{O.3} + r_{O.4b} - r_{O.5} = 0; \quad r_{O.5} = r_{O.3} + r_{O.4b} \quad \text{(S14.22)}$$

Pseudo steady state approximation on $[O^-*]$:

$$\frac{d[O^-*]}{dt} = r_{O.4a} - r_{O.4b} = 0; \quad r_{O.4a} = r_{O.4b} \quad \text{(S14.23)}$$

Substituting Eqns. S14.18–23 into the left side of Eqn. S14.16, the sum of reduction steps collapse to a single rate:

$$r_{O.2} + r_{O.4a} + r_{O.5a} + r_{O.5c} = r_{O.2} + r_{O.2} + r_{O.2} + r_{O.2} = 4r_{O.2} \quad \text{(S14.24)}$$

Repeating this process for the oxidation steps:

Pseudo steady state approximation on $[RCHOH*_g]$:

$$\frac{d[RCHOH*_g]}{dt} = r_{G.2b} - r_{G.3a} = 0; \quad r_{G.2b} = r_{G.3a} \quad \text{(S14.25)}$$

Pseudo steady state approximation on $[RCH_2OH^+*_g]$:

$$\frac{d[RCH_2OH^+*_g]}{dt} = r_{G.2a} - r_{G.2b} = 0; \quad r_{G.2a} = r_{G.2b} = r_{G.3a} \quad \text{(S14.26)}$$

Substituting Eqns. S14.25–26 into the right side of Eqn. S14.16, the sum of oxidation steps collapse to a single rate:

$$r_{G.2a} + r_{G.3a} = r_{G.2a} + r_{G.2a} = 2r_{G.2a} \quad \text{(S14.27)}$$

Combining Eqns. S14.24 and S14.27 in Eqn. S14.16:

$$4r_{O.2} = 2r_{G.2a} \tag{S14.28}$$

$$4[\ast]_T\left(k^{0\prime}_{O-1}\frac{[OO\ast]}{[\ast]_T}e^{\frac{-\beta_{O-1}FE_{cat}}{RT}}\right) = 2[\ast_g]_T\left(k^{0\prime}_{C-H}K_{GNA}a_{GNA}\left(\frac{[\ast_g]}{[\ast_g]_T}\right)e^{\frac{\beta_{C-H}FE_{cat}}{RT}}\right) \tag{S14.29}$$

Rearranging Eqn. S14.29 to isolate E_{cat}:

$$\left(\frac{4k^{0\prime}_{O-1}[\ast]_T}{2k^{0\prime}_{C-H}[\ast_g]_T}\right)\frac{\left(\frac{[OO\ast]}{[\ast]_T}\right)}{\left(\frac{[RCH_2OH\ast_g]}{[\ast_g]_T}\right)} = \frac{e^{\frac{\beta_{C-H}FE_{cat}}{RT}}}{e^{\frac{-\beta_{O-1}FE_{cat}}{RT}}} \tag{S14.30}$$

Quasi equilibrium approximation for step O.1:

$$K_{O_2} = \frac{[OO\ast]}{[\ast]a_{O_2}},\ [OO\ast] = K_{O_2}a_{O_2}[\ast] \tag{S14.31}$$

Substituting Eqns. S14.6 and S14.31 into Eqn. S14.30:

$$\left(\frac{4k^{0\prime}_{O-1}K_{O_2}[\ast]_T}{2k^{0\prime}_{C-H}K_{GNA}[\ast_g]_T}\right)\left(\frac{a_{O_2}}{a_{GNA}}\right)\frac{\left(\frac{[\ast]}{[\ast]_T}\right)}{\left(\frac{[\ast_g]}{[\ast_g]_T}\right)} = e^{(\beta_{C-H}+\beta_{O-1})\frac{FE_{cat}}{RT}} \tag{S14.32}$$

Rearranging:

$$E_{cat} = \frac{RT}{(\beta_{C-H}+\beta_{O-1})F}\ln\left(\left(\frac{4k^{0\prime}_{O-1}K_{O_2}[\ast]_T}{2k^{0\prime}_{C-H}K_{GNA}[\ast_g]_T}\right)\left(\frac{a_{O_2}}{a_{GNA}}\right)\frac{\left(\frac{[\ast]}{[\ast]_T}\right)}{\left(\frac{[\ast_g]}{[\ast_g]_T}\right)}\right) \tag{S14.33}$$

Without making any further assumptions, we substitute Eqn. S14.33 into S14.7:

$$\frac{-r_{GNA}}{[\ast_g]_T} = k^{0\prime}_{C-H}K_{GNA}a_{GNA}\left(\frac{[\ast_g]}{[\ast_g]_T}\right)e^{\frac{\beta_{C-H}F}{RT}\left(\frac{RT}{(\beta_{C-H}+\beta_{O-1})F}\ln\left(\left(\frac{4k^{0\prime}_{O-1}K_{O_2}[\ast]_T}{2k^{0\prime}_{C-H}K_{GNA}[\ast_g]_T}\right)\left(\frac{a_{O_2}}{a_{GNA}}\right)\frac{\left(\frac{[\ast]}{[\ast]_T}\right)}{\left(\frac{[\ast_g]}{[\ast_g]_T}\right)}\right)\right)} \tag{S14.34}$$

Simplifying:

$$\frac{-r_{GNA}}{[\ast_g]_T} = k^{0\prime}_{C-H}K_{GNA}a_{GNA}\left(\frac{[\ast_g]}{[\ast_g]_T}\right)\left(\frac{4k^{0\prime}_{O-1}K_{O_2}[\ast]_T}{2k^{0\prime}_{C-H}K_{GNA}[\ast_g]_T}\right)\left(\frac{a_{O_2}}{a_{GNA}}\right)\frac{\left(\frac{[\ast]}{[\ast]_T}\right)}{\left(\frac{[\ast_g]}{[\ast_g]_T}\right)}e^{\left(\frac{\beta_{C-H}}{\beta_{C-H}+\beta_{O-1}}\right)} \tag{S14.35}$$

$$\frac{-r_{GNA}}{[\ast]_T} = 2k^{0\prime}_{O-1}K_{O_2}a_{O_2}\left(\frac{[\ast]}{[\ast]_T}\right)e^{\left(\frac{\beta_{C-H}}{\beta_{C-H}+\beta_{O-1}}\right)} \tag{S14.36}$$

Limiting Case 1: Alcohol Oxidation and O_2 Reduction Occur on the Same Active Site

In this case, $[\ast] = [\ast_g]$:

The catalyst potential is now independent of coverage, and Eqn. S14.33 simplifies to:

$$E_{cat} = \frac{RT}{(\beta_{C-H}+\beta_{O-1})F}\ln\left(\left(\frac{4k_{O-1}^{0'}K_{O_2}}{2k_{C-H}^{0'}K_{GNA}}\right)\left(\frac{a_{O_2}}{a_{GNA}}\right)\right) \tag{S14.37}$$

The linearized form of Eqn. S14.37 is:

$$E_{cat} = \left[\frac{RT}{(\beta_{C-H}+\beta_{O-1})F}\ln\left(\frac{4k_{O-1}^{0'}K_{O_2}}{2k_{C-H}^{0'}K_{GNA}}\right)\right] + \left[\frac{RT}{(\beta_{C-H}+\beta_{O-1})F}\right]\ln\left(\frac{a_{O_2}}{a_{GNA}}\right) \tag{S14.38}$$

where $\left[\frac{RT}{(\beta_{C-H}+\beta_{O-1})F}\right]$ equals the slope between $\ln\left(\frac{a_{O_2}}{a_{GNA}}\right)$ and E_{cat}. The logarithmic dependence of E_{cat} on the O_2 to GNA ratio agrees with the measured E_{cat} values reported in Figure 4 of the main text.

For the site balance, we recognize that the negatively charged active hydroxyl moieties, which are explicitly accounted for in the pseudo steady state balance, are equivalent to the overall charge balance. To illustrate this point algebraically:

$$\frac{d[HO^- *]}{dt} = (r_{O.2} + r_{O.3} + r_{O.5b} + r_{O.5c}) - (r_{G.2b} + r_{G.2c}) = 0 \tag{S14.39}$$

Substituting Eqns. S14.24 and S14.27 in Eqn. S14.39:

$$\frac{d[HO^- *]}{dt} = 4r_{O.2} - 2r_{G.2a} = 0$$

(S14.40)

$$4k_{O-1}^{0'}([OO *])e^{\frac{-\beta_{O-1}FE_{cat}}{RT}} = 2k_{C-H}^{0'}[RCH_2OH *]e^{\frac{\beta_{C-H}FE_{cat}}{RT}} \tag{S14.41}$$

Eqn. S14.41 is identical to Eqn. 14.29. For the hydroxide $[HO^- *]$ to become negatively polarized, the Pt surface is equally and oppositely positively polarized. The polarized Pt surface, described by:

Pt.1. $$* \rightleftharpoons *^+ + e^-$$

acts as a surrogate for polarized $HO^- *$ via the equilibrium expression that retains electrical neutrality:

$$* + HO * \rightleftharpoons *^+ + HO^- *$$

Step Pt.1 formalizes e$^-$ transfer through the Pt nanoparticle. Step Pt.1 is described by the Nernst equation:

$$E_{cat} = E^0 - \frac{RT}{F}\ln\left(\frac{[*]}{[*^+]}\right) \tag{S14.42}$$

$$\frac{[*^+]}{[*]} = K_{Pt}^0 e^{\left(\frac{FE_{cat}}{RT}\right)} \tag{S14.43}$$

Where $K_{Pt}^0 = e^{\frac{-FE^0}{RT}}$.

The overall site balance is:

$$[L] = [*] + [*^+] + [OO *] + [RCH_2OH *] \tag{S14.44}$$

Substituting Eqns. S14.6, S14.31, and S14.43 into Eqn. S14.44:

$$\frac{[L]}{[*]} = 1 + K_{Pt}^{0} e^{\left(\frac{FE_{cat}}{RT}\right)} + K_{O_2} a_{O_2} + K_{GNA} a_{GNA} \tag{S14.45}$$

Substituting S14.45 into Eqn. S14.7 gives the rate expression:

$$\frac{-r_{GNA}}{[L]} = \frac{k_{C-H}^{0\,\prime} K_{GNA} a_{GNA} e^{\frac{\beta_{C-H} F E_{cat}}{RT}}}{1 + K_{Pt}^{0} e^{\left(\frac{FE_{cat}}{RT}\right)} + K_{O_2} a_{O_2} + K_{GNA} a_{GNA}} \tag{S14.46}$$

Substituting Eqn. S14.37 into Eqn. S14.46:

$$\frac{-r_{GNA}}{[L]} = \frac{k_{C-H}^{0\,\prime} K_{GNA} a_{GNA} e^{\frac{\beta_{C-H} F}{RT} \frac{RT}{(\beta_{C-H} + \beta_{O-1}) F} \ln\left(\left(\frac{4k_{O-1}^{0\,\prime} K_{O_2}}{2k_{C-H}^{0\,\prime} K_{GNA}}\right)\left(\frac{a_{O_2}}{a_{GNA}}\right)\right)}}{1 + K_{Pt}^{0} e^{\left(\frac{F}{RT} \frac{RT}{(\beta_{C-H} + \beta_{O-1}) F} \ln\left(\left(\frac{4k_{O-1}^{0\,\prime} K_{O_2}}{2k_{C-H}^{0\,\prime} K_{GNA}}\right)\left(\frac{a_{O_2}}{a_{GNA}}\right)\right)\right)} + K_{O_2} a_{O_2} + K_{GNA} a_{GNA}} \tag{S14.47}$$

Simplifying:

$$\frac{-r_{GNA}}{[L]} = \frac{2k_{O-1}^{0\,\prime} K_{O_2} a_{O_2} e^{\left(\frac{\beta_{C-H}}{\beta_{C-H} + \beta_{O-1}}\right)}}{1 + \left(\frac{2k_{O-1}^{0\,\prime} K_{O_2} K_{Pt}^{0}}{k_{C-H}^{0\,\prime} K_{GNA}} e^{\left(\frac{1}{\beta_{C-H} + \beta_{O-1}}\right)}\right)\left(\frac{a_{O_2}}{a_{GNA}}\right) + K_{O_2} a_{O_2} + K_{GNA} a_{GNA}} \tag{S14.48}$$

Eqn. S14.48 has an identical functional form to the thermocatalytic rate equation provided in the main text, Eqn. S13.2 in Section S13.

Alternative Case 1: C-H Scission is Solution Mediated

In this case, the two half-reactions are noninteracting and exactly equivalent to their electrochemical half-reactions, with proton shuttling from H_3O^+. We denote electron and proton transfer as separate steps, but combining these into a single elementary step would only add an $a_{H_3O^+}$ contribution to each O_2 reduction step, a value which is implicitly accounted for by the conversion of E_{cat} to a reversible hydrogen electrode scale.

O_2 Reduction Half-Reaction

O.1.	$* + O_2 \rightleftharpoons OO*$	K_{O_2}
O.2e.	$OO* + e^- \rightarrow OO^-*$	k_{O-1e}
O.2p.	$OO^-* + H_3O^+ \rightarrow H_2O + HOO*$	k_{O-1p}
O.3.	$HOO* + * \rightarrow HO* + O*$	k_{O-2}
O.4e.	$O* + e^- \rightarrow O^-*$	k_{O-3e}
O.4p.	$O^-* + H_3O^+ \rightarrow HO*$	k_{O-3p}

O.5e. $HO* + e^{-} \rightarrow H_2O + HO^{-}*$ k_{O-4e}

O.5p. $HO^{-}* + H_3O^{+} \rightarrow 2\,H_2O + *$ k_{O-4p}

Net O_2 reduction half-reaction:

ORR $O_2 + 4\,e^{-} + 4\,H_3O^{+} \rightarrow 6\,H_2O$ K_{ORR}

Alcohol Oxidation Half-Reaction

G.1. $*_g + RCH_2OH \rightleftharpoons RCH_2OH*_g$ K_{GNA}

G.2e. $RCH_2OH*_g \rightarrow RCH_2OH^{+}*_g + e^{-}$ $k_{C-H,e}$

G.2p. $RCH_2OH^{+}*_g + H_2O \nrightarrow RCHOH*_g + H_3O^{+}$ $k_{C-H,p}$

G.3e. $RCHOH*_g \rightarrow RCHOH^{+}*_g + e^{-}$ $k_{O-H,e}$

G.3p. $RCHOH^{+}*_g + H_2O \rightarrow RCHO*_g + H_3O^{+}$ $k_{O-H,p}$

G.4. $RCHO*_g \rightleftharpoons *_g + RCHO$ K_{GLA}^{-1}

Net alcohol oxidation half-reaction:

GOR $RCH_2OH + 2\,H_2O \rightarrow RCHO + 2\,e^{-} + 2\,H_3O^{+}$ K_{GOR}

Net thermochemical reaction:

$$2\,RCH_2OH + O_2 \rightarrow 2\,RCHO + 2\,H_2O$$

Nomenclature:

R = $C_5H_9O_6$ for GNA; $*$: O_2 reduction active site; $*_g$: GNA binding multi-site ensemble;

$\rightleftharpoons$:Quasi equilibrated; $\rightarrow$:irreversible; $\nrightarrow$: kinetically-relevant

Rate Equation Derivation

We derive the rate equation starting from the kinetically relevant step of proton abstraction in C-H bond scission, Step G.2p:

$$\frac{-r_{GNA}}{[*_g]_T} = r_{G.2p} = k_{C-H,p}\left(\frac{[RCH_2OH^{+}*_g]}{[*_g]_T}\right)a_{H_2O} \quad \text{(S14.49)}$$

Pseudo steady state balance on $[RCH_2OH^{+}*_g]$:

$$\frac{d[RCH_2OH^{+}*_g]}{dt} = 0 = r_{G.2e} - r_{G.2p} \quad \text{(S14.50)}$$

$$\frac{-r_{GNA}}{[*_g]_T} = k^{0}_{C-H,e}\left(\frac{[RCH_2OH*_g]}{[*_g]_T}\right)e^{\frac{\beta_{C-H}F\left(E_{cat}-E^{0}_{C-H}\right)}{RT}} \quad \text{(S14.51)}$$

Quasi equilibrium approximation for step G.1:

$$K_{GNA} = \frac{[RCH_2OH*_g]}{[*_g]a_{GNA}},\ [RCH_2OH*_g] = K_{GNA}a_{GNA}[*_g] \tag{S14.52}$$

Substituting Eqn. S14.52 into Eqn. S14.51:

$$\frac{-r_{GNA}}{[*_g]_T} = k^0_{C-H,e}K_{GNA}a_{GNA}\left(\frac{[*_g]}{[*_g]_T}\right)e^{\frac{\beta_{C-H}F\left(E_{cat}-E^0_{C-H}\right)}{RT}} \tag{S14.53}$$

Or, simplified according to Eqn. S14.4:

$$\frac{-r_{GNA}}{[*_g]_T} = k^{0\prime}_{C-H}K_{GNA}a_{GNA}\left(\frac{[*_g]}{[*_g]_T}\right)e^{\frac{\beta_{C-H}FE_{cat}}{RT}} \tag{S14.54}$$

For the electron balance at open circuit:

$$-\sum i_{red} = \sum i_{ox} \tag{S14.55}$$

The following rate expressions capture the elementary steps that depend on E_{cat}:

Reduction rates:

$$\frac{r_{O.2e}}{[*]_T} = k^{0\prime}_{O-1}\frac{[OO*]}{[*]_T}e^{\frac{-\beta_{O-1}FE_{cat}}{RT}} \tag{S14.56}$$

$$\frac{r_{O.4e}}{[*]_T} = k^{0\prime}_{O-3}\frac{[O*]}{[*]_T}e^{\frac{-\beta_{O-3}FE_{cat}}{RT}} \tag{S14.57}$$

$$\frac{r_{O.5e}}{[*]_T} = k^{0\prime}_{O-4}\frac{[HO*]}{[*]_T}e^{\frac{-\beta_{O-4}FE_{cat}}{RT}} \tag{S14.58}$$

Oxidation rates:

$$\frac{r_{G.2e}}{[*_g]_T} = k^{0\prime}_{C-H}\frac{[RCH_2OH*_g]}{[*_g]_T}e^{\frac{\beta_{C-H}FE_{cat}}{RT}} \tag{S14.59}$$

$$\frac{r_{G.3e}}{[*_g]_T} = k^{0\prime}_{O-H}\frac{[RCHOH*_g]}{[*_g]_T}e^{\frac{\beta_{O-H}FE_{cat}}{RT}} \tag{S14.60}$$

Eqn. S14.55 is therefore:

$$r_{O.2e} + r_{O.4e} + r_{O.5e} = r_{G.2e} + r_{G.3e} \tag{S14.61}$$

Substituting Eqns. S14.56–60 into Eqn. S14.61:

$$\left(k^{0\prime}_{O-1}\,\frac{[OO*]}{[*]_T}\,e^{\frac{-\beta_{O-1}FE_{cat}}{RT}} + k^{0\prime}_{O-3}\,\frac{[O*]}{[*]_T}\,e^{\frac{-\beta_{O-3}FE_{cat}}{RT}} + k^{0\prime}_{O-4}\,\frac{[HO*]}{[*]_T}\,e^{\frac{-\beta_{O-4}FE_{cat}}{RT}}\right) = \left(\frac{[*_g]_T}{[*]_T}\right)$$
$$\left(k^{0\prime}_{C-H}\,\frac{[RCH_2OH*_g]}{[*_g]_T}\,e^{\frac{\beta_{C-H}FE_{cat}}{RT}} + k^{0\prime}_{O-H}\,\frac{[RCHOH*_g]}{[*_g]_T}\,e^{\frac{\beta_{O-H}FE_{cat}}{RT}}\right) \tag{S14.62}$$

Pseudo steady state approximation on $[O*]$:

$$\frac{d[O*]}{dt} = r_{O.3} - r_{O.4e} = 0; \quad r_{O.4e} = r_{O.3} \tag{S14.63}$$

Pseudo steady state approximation on $[HOO*]$:

$$\frac{d[HOO*]}{dt} = r_{O.2p} - r_{O.3} = 0; \quad r_{O.2p} = r_{O.3} \tag{S14.64}$$

Pseudo steady state approximation on $[OO^- *]$:

$$\frac{d[OO^-*]}{dt} = r_{O.2e} - r_{O.2p} = 0; \quad r_{O.2p} = r_{O.2e} \tag{S14.65}$$

Combining Eqns. S14.63–S14.65:

$$r_{O.4e} = r_{O.2e} = k^{0\prime}_{O-1}[OO *]e^{\frac{-\beta_{O-1}FE_{cat}}{RT}} \tag{S14.66}$$

Pseudo steady state approximation on $[HO *]$:

$$\frac{d[HO*]}{dt} = r_{O.3} + r_{O.4p} - r_{O.5e} = 0; \quad r_{O.5e} = r_{O.3} + r_{O.4p} \tag{S14.67}$$

Pseudo steady state approximation on $[O^- *]$:

$$\frac{d[O^-*]}{dt} = r_{O4.e} - r_{O4.p} = 0; \quad r_{O4.e} = r_{O4.p} \tag{S14.68}$$

Combining Eqns. S14.66–S14.68:

$$r_{O.5e} = r_{O.3} + r_{O4.e} = 2r_{O4.e} = 2r_{O.2e} = 2k^{0\prime}_{O-1}[OO *]e^{\frac{-\beta_{O-1}FE_{cat}}{RT}} \tag{S14.69}$$

Substituting Eqns. S14.64–S14.66 and S14.69 into the left side of Eqn. S14.61:

$$r_{O.2e} + r_{O.4e} + r_{O.5e} = r_{O.2e} + r_{O.2e} + 2r_{O.2e} = 4r_{O.2e} \tag{S14.70}$$

Pseudo steady state approximation on $[RCHOH *_g]$:

$$\frac{d[RCHOH*_g]}{dt} = r_{G.2p} - r_{G.3e} = 0; \quad r_{G.3e} = r_{G.2p} \tag{S14.71}$$

Pseudo steady state approximation on $[RCH_2OH^+ *_g]$:

$$\frac{d[RCH_2OH^+*_g]}{dt} = r_{G.2e} - r_{G.2p} = 0; \quad r_{G.2p} = r_{G.2e} \tag{S14.72}$$

Combining Eqns. S14.71 and S14.72:

$$r_{G.3e} = r_{G.2e} = k^{0\prime}_{C-H}[RCH_2OH *_g]e^{\frac{\beta_{C-H}FE_{cat}}{RT}} \tag{S14.73}$$

Substituting Eqn. S14.73 into the right side of Eqn. S14.61:

$$r_{G.2e} + r_{G.3e} = r_{G.2e} + r_{G.2e} = 2r_{G.2e} \tag{S14.74}$$

Combining Eqns. S14.70 and S14.74 into Eqn. S14.61:

$$4r_{O.2e} = 2r_{G.2e} \tag{S14.75}$$

$$4k^{0\prime}_{O-1}\frac{[OO*]}{[*]_T}e^{\frac{-\beta_{O-1}FE_{cat}}{RT}} = \left(\frac{[*_g]_T}{[*]_T}\right)\left(2k^{0\prime}_{C-H}\frac{[RCH_2OH*_g]}{[*_g]_T}e^{\frac{\beta_{C-H}FE_{cat}}{RT}}\right) \tag{S14.76}$$

Rearranging:

$$\left(\frac{4k^{0\prime}_{O-1}[*]_T}{2k^{0\prime}_{C-H}[*_g]_T}\right)\frac{\left(\frac{[OO*]}{[*]_T}\right)}{\left(\frac{[RCH_2OH*_g]}{[*_g]_T}\right)} = \frac{e^{\frac{\beta_{C-H}FE_{cat}}{RT}}}{e^{\frac{-\beta_{O-1}FE_{cat}}{RT}}} \tag{S14.77}$$

Quasi equilibrium approximation for step O.1:

$$K_{O_2} = \frac{[OO*]}{[*]a_{O_2}},\ [OO*] = K_{O_2}a_{O_2}[*] \tag{S14.78}$$

Substituting Eqns. S14.52 and S14.78 into Eqn. S14.77:

$$\left(\frac{4k^{0'}_{O-1}K_{O_2}[*]_T}{2k^{0'}_{C-H}K_{GNA}[*_g]_T}\right)\left(\frac{a_{O_2}}{a_{GNA}}\right)\frac{\left(\frac{[*]}{[*]_T}\right)}{\left(\frac{[*_g]}{[*_g]_T}\right)} = e^{(\beta_{C-H}+\beta_{O-1})\frac{FE_{cat}}{RT}} \tag{S14.79}$$

$$E_{cat} = \frac{RT}{(\beta_{C-H}+\beta_{O-1})F}\ln\left(\left(\frac{4k^{0'}_{O-1}K_{O_2}[*]_T}{2k^{0'}_{C-H}K_{GNA}[*_g]_T}\right)\left(\frac{a_{O_2}}{a_{GNA}}\right)\frac{\left(\frac{[*]}{[*]_T}\right)}{\left(\frac{[*_g]}{[*_g]_T}\right)}\right) \tag{S14.80}$$

Substituting Eqn. S14.80 into Eqn. S14.54:

$$\frac{-r_{GNA}}{[*_g]_T} = k^{0'}_{C-H}K_{GNA}a_{GNA}\left(\frac{[*_g]}{[*_g]_T}\right)e^{\frac{\beta_{C-H}F}{RT}\left(\frac{RT}{(\beta_{C-H}+\beta_{O-1})F}\ln\left(\left(\frac{4k^{0'}_{O-1}K_{O_2}[*]_T}{2k^{0'}_{C-H}K_{GNA}[*_g]_T}\right)\left(\frac{a_{O_2}}{a_{GNA}}\right)\frac{\left(\frac{[*]}{[*]_T}\right)}{\left(\frac{[*_g]}{[*_g]_T}\right)}\right)\right)} \tag{S14.81}$$

Simplifying:

$$\frac{-r_{GNA}}{[*_g]_T} = k^{0'}_{C-H}K_{GNA}a_{GNA}\left(\frac{[*_g]}{[*_g]_T}\right)\left(\frac{4k^{0'}_{O-1}K_{O_2}[*]_T}{2k^{0'}_{C-H}K_{GNA}[*_g]_T}\right)\left(\frac{a_{O_2}}{a_{GNA}}\right)\frac{\left(\frac{[*]}{[*]_T}\right)}{\left(\frac{[*_g]}{[*_g]_T}\right)}e^{\left(\frac{\beta_{C-H}}{\beta_{C-H}+\beta_{O-1}}\right)} \tag{S14.82}$$

$$\frac{-r_{GNA}}{[*]_T} = 2k^{0'}_{O-1}K_{O_2}a_{O_2}\left(\frac{[*]}{[*]_T}\right)e^{\left(\frac{\beta_{C-H}}{\beta_{C-H}+\beta_{O-1}}\right)} \tag{S14.83}$$

Site Balances:

Two interconnected site balances:

$$[*_g]_T = [*_g] + [RCH_2OH*_g],\quad [*]_T = [*] + [OO*] + [HO*] \tag{S14.84}$$

Substituting Eqns. S14.52 and S14.78 into Eqn. S14.84:

$$\frac{[*_g]_T}{[*_g]} = 1 + K_{GNA}a_{GNA},\quad [*]_T = [*] + K_{O_2}a_{O_2}[*] + [HO*] \tag{S14.85}$$

Pseudo steady state balance on $[HO*]$ (continued from Eqn. S14.67):

$$\frac{d[HO*]}{dt} = r_{O.3} + r_{O.4p} - r_{O.5e} = 0;\quad r_{O.5e} = 2r_{O.2e} \tag{S14.86}$$

$$k^{0'}_{O-4}[HO*]e^{\frac{-\beta_{O-4}FE_{cat}}{RT}} = 2k^{0'}_{O-1}[OO*]e^{\frac{-\beta_{O-1}FE_{cat}}{RT}} \tag{S14.87}$$

$$[HO*] = \frac{2k^{0'}_{O-1}K_{O_2}a_{O_2}}{k^{0'}_{O-4}}[*]e^{\frac{-(\beta_{O-1}-\beta_{O-4})FE_{cat}}{RT}} \tag{S14.88}$$

Substituting Eqn. S14.80 into Eqn. S14.88:

$$[HO*] = \frac{2k_{O-1}^{0'}K_{O_2}a_{O_2}}{k_{O-4}^{0'}}[*]\left(\frac{1}{e^{\frac{(\beta_{O-1}-\beta_{O-4})F}{RT}\left(\frac{RT}{(\beta_{C-H}+\beta_{O-1})F}\ln\left(\left(\frac{4k_{O-1}^{0'}K_{O_2}[*]_T}{2k_{C-H}^{0'}K_{GNA}[*g]_T}\right)\left(\frac{a_{O_2}}{a_{GNA}}\right)\frac{\left(\frac{[*]}{[*]_T}\right)}{\left(\frac{[*g]}{[*g]_T}\right)}\right)\right)}}\right) \quad \text{(S14.89)}$$

$$[HO*] = \frac{2k_{O-1}^{0'}K_{O_2}a_{O_2}}{k_{O-4}^{0'}e^{\frac{(\beta_{O-1}-\beta_{O-4})}{(\beta_{C-H}+\beta_{O-1})}}}[*]\left(\frac{1}{\left(\frac{4k_{O-1}^{0'}K_{O_2}}{2k_{C-H}^{0'}K_{GNA}[*g]_T}\right)\left(\frac{a_{O_2}}{a_{GNA}}\right)\frac{([*])}{\left(\frac{1}{1+K_{GNA}a_{GNA}}\right)}}\right) \quad \text{(S14.90)}$$

$$[HO*] = \frac{k_{C-H}^{0'}K_{GNA}[*g]_T a_{GNA}}{k_{O-4}^{0'}e^{\frac{(\beta_{O-1}-\beta_{O-4})}{(\beta_{C-H}+\beta_{O-1})}}(1+K_{GNA}a_{GNA})} \quad \text{(S14.91)}$$

Substituting Eqn. S14.91 into Eqn. S14.85:

$$[*]_T\left(1-\frac{k_{C-H}^{0'}K_{GNA}\left(\frac{[*g]_T}{[*]_T}\right)a_{GNA}}{k_{O-4}^{0'}e^{\frac{(\beta_{O-1}-\beta_{O-4})}{(\beta_{C-H}+\beta_{O-1})}}(1+K_{GNA}a_{GNA})}\right) = [*] + K_{O_2}a_{O_2}[*] \quad \text{(S14.92)}$$

$$\frac{[*]}{[*]_T} = \frac{\left(1-\frac{k_{C-H}^{0'}K_{GNA}\left(\frac{[*g]_T}{[*]_T}\right)a_{GNA}}{k_{O-4}^{0'}e^{\frac{(\beta_{O-1}-\beta_{O-4})}{(\beta_{C-H}+\beta_{O-1})}}(1+K_{GNA}a_{GNA})}\right)}{1+K_{O_2}a_{O_2}} \quad \text{(S14.93)}$$

Substituting Eqn. S14.93 into Eqn. S14.83:

$$\frac{-r_{GNA}}{[*]_T} = 2k_{O-1}^{0'}K_{O_2}a_{O_2}\frac{\left(1-\frac{k_{C-H}^{0'}K_{GNA}\left(\frac{[*g]_T}{[*]_T}\right)a_{GNA}}{k_{O-4}^{0'}e^{\frac{(\beta_{O-1}-\beta_{O-4})}{(\beta_{C-H}+\beta_{O-1})}}(1+K_{GNA}a_{GNA})}\right)}{1+K_{O_2}a_{O_2}}e^{\left(\frac{\beta_{C-H}}{\beta_{C-H}+\beta_{O-1}}\right)} \quad \text{(S14.94)}$$

$$\frac{-r_{GNA}}{[*]_T} = 2k_{O-1}^{0'}K_{O_2}e^{\left(\frac{\beta_{C-H}}{\beta_{C-H}+\beta_{O-1}}\right)}\left(\frac{a_{O_2}}{1+K_{O_2}a_{O_2}}\right)\left(1-\frac{k_{C-H}^{0'}K_{GNA}\left(\frac{[*g]_T}{[*]_T}\right)}{k_{O-4}^{0'}e^{\frac{(\beta_{O-1}-\beta_{O-4})}{(\beta_{C-H}+\beta_{O-1})}}}\left(\frac{a_{GNA}}{1+K_{GNA}a_{GNA}}\right)\right) \quad \text{(S14.95)}$$

The functional form of Eqn. S14.95 matches Eqn. S13.3 (Model 3 in Section S13), which can accurately capture the kinetic results.

Furthermore, we substitute the coverage terms to describe E_{cat}:

$$E_{cat} = \frac{RT}{(\beta_{C-H}+\beta_{O-1})F}\ln\left(\left(\frac{4k_{O-1}^{0'}K_{O_2}[*]_T}{2k_{C-H}^{0'}K_{GNA}[*_g]_T}\right)\left(\frac{a_{O_2}}{a_{GNA}}\right)\frac{\frac{\left(1-\frac{k_{C-H}^{0'}K_{GNA}\left(\frac{[*_g]_T}{[*]_T}\right)a_{GNA}}{k_{O-4}^{0'}e^{\left(\frac{\beta_{O-1}-\beta_{O-4}}{\beta_{C-H}+\beta_{O-1}}\right)}(1+K_{GNA}a_{GNA})}\right)}{1+K_{O_2}a_{O_2}}}{\left(\frac{1}{(1+K_{GNA}a_{GNA})}\right)}\right) \quad \text{(S14.96)}$$

$$E_{cat} = \frac{RT}{(\beta_{C-H}+\beta_{O-1})F}\ln\left(\left(\frac{4k_{O-1}^{0'}K_{O_2}[*]_T}{2k_{C-H}^{0'}K_{GNA}[*_g]_T}\right)\left(\frac{a_{O_2}}{a_{GNA}}\right)\frac{(1+K_{GNA}a_{GNA})}{\left(1+K_{O_2}a_{O_2}\right)}\left(1-\frac{k_{C-H}^{0'}K_{GNA}\left(\frac{[*_g]_T}{[*]_T}\right)a_{GNA}}{k_{O-4}^{0'}e^{\left(\frac{\beta_{O-1}-\beta_{O-4}}{\beta_{C-H}+\beta_{O-1}}\right)}(1+K_{GNA}a_{GNA})}\right)\right)$$

(S14.97)

Eqn. S14.97 does not accurately capture the measured E_{cat} results, because we observe a monotonic dependence of E_{cat} on the logarithm of the O_2 to GNA ratio in Figure 4c of the main text. Therefore, this model is insufficient to explain all kinetic observations.

Limiting Case 1.1: Alcohol Oxidation and O_2 Reduction Occur on the Same Site and are Solution Mediated

If $[*] = [*_g]$, Eqn. S14.80 becomes:

$$E_{cat} = \frac{RT}{(\beta_{C-H}+\beta_{O-1})F}\ln\left(\left(\frac{4k_{O-1}^{0'}K_{O_2}}{2k_{C-H}^{0'}K_{GNA}}\right)\left(\frac{a_{O_2}}{a_{GNA}}\right)\right) \quad \text{(S14.98)}$$

and Eqn. S14.83 becomes:

$$\frac{-r_{GNA}}{[*]_T} = 2k_{O-1}^{0'}K_{O_2}a_{O_2}\left(\frac{[*]}{[*]_T}\right)e^{\left(\frac{\beta_{C-H}}{\beta_{C-H}+\beta_{O-1}}\right)} \quad \text{(S14.99)}$$

The Pt charge balancing technique used above, which described the coverage of $[HO^- *]$ using $[*^+]$ as proxy, does not apply here because O=O activation and C-H activation are solution mediated (i.e., we do not have an active oxygen species that is bound to Pt).

The site balance is:

$$[*]_T = [*] + [HO*] + [OO*] + [RCH_2OH*] \quad \text{(S14.100)}$$

Simplifying Eqn. S14.89 for a single active site:

$$[HO*] = \frac{k_{C-H}^{0'} K_{GNA} a_{GNA}}{k_{O-4}^{0'} e^{\frac{(\beta_{O-1}-\beta_{O-4})}{(\beta_{C-H}+\beta_{O-1})}}}[*] \tag{S14.101}$$

Substituting Eqns. S14.52, S14.78, and S14.101 into Eqn. S14.100:

$$\frac{[*]_T}{[*]} = 1 + K_{O_2} a_{O_2} + \left(1 + \frac{k_{C-H}^{0'}}{k_{O-4}^{0'} e^{\frac{(\beta_{O-1}-\beta_{O-4})}{(\beta_{C-H}+\beta_{O-1})}}}\right) K_{GNA} a_{GNA} \tag{S14.102}$$

Substituting Eqn. S14.102 into Eqn. S14.99:

$$\frac{-r_{GNA}}{[*]_T} = \frac{2k_{O-1}^{0'} K_{O_2} a_{O_2} e^{\left(\frac{\beta_{C-H}}{\beta_{C-H}+\beta_{O-1}}\right)}}{1+K_{O_2} a_{O_2} + \left(1+\frac{k_{C-H}^{0'}}{k_{O-4}^{0'} e^{\frac{(\beta_{O-1}-\beta_{O-4})}{(\beta_{C-H}+\beta_{O-1})}}}\right) K_{GNA} a_{GNA}} \tag{S14.103}$$

Eqn. S14.103 does not recover the measured kinetic dependencies because there is no positive order dependence on a_{GNA}. This arises because all surface intermediates in the oxidation and reduction half-reactions are independent, and do not "cross-talk" between the two half-reactions, so net turnover rates are controlled exclusively by the rate of O_2 activation.

S15. Derivation of the Enthalpic and Entropic Contributions to Rates and E_{cat}

GNA oxidation rates ($-r_{GNA}$) and catalyst electrode potential at open circuit (E_{cat}) depend on temperature with slopes that correspond to elementary thermodynamic quantities embedded in the kinetic parameters. We quantify these slopes in terms of the partial derivatives of $-r_{GNA}$ and E_{cat} with respect to inverse temperature ($\partial/\partial(1/T)$) or temperature ($\partial/\partial T$).

Analytical Expression Relating $\partial E_{cat}/\partial T$ to the Activation Enthalpies and Entropies

The analytical relationship between E_{cat} and O_2/GNA is (Eqn. S14.38):

$$E_{cat} = \left[\frac{RT}{(\beta_{C-H}+\beta_{O-1})F}\ln\left(\frac{4k^{0'}_{O-1}K_{O_2}}{2k^{0'}_{C-H}K_{GNA}}\right)\right] + \left[\frac{RT}{(\beta_{C-H}+\beta_{O-1})F}\right]\ln\left(\frac{a_{O_2}}{a_{GNA}}\right) \quad \text{(S15.1)}$$

For $k^{0'}_{O-1}$, K_{O_2}, $k^{0'}_{C-H}$, and K_{GNA}, the Eyring and van 't Hoff relations, following transition state theory, relate the rate and equilibrium constants to their activation and adsorption free energies:

$$k^{0'}_{O-1} = \frac{k_BT}{h}e^{\frac{-\Delta G^{\ddagger}_{O-1}}{RT}}e^{\frac{\beta_{O-1}FE^{0}_{0-1}}{RT}} \quad \text{(S15.2)}$$

$$K_{O_2} = e^{\frac{-\Delta G_{ads,O_2}}{RT}} \quad \text{(S15.3)}$$

$$k^{0'}_{C-H} = \frac{k_BT}{h}e^{\frac{-\Delta G^{\ddagger}_{C-H}}{RT}}e^{\frac{-\beta_{C-H}FE^{0}_{C-H}}{RT}} \quad \text{(S15.4)}$$

$$K_{GNA} = e^{\frac{-\Delta G_{ads,GNA}}{RT}} \quad \text{(S15.5)}$$

Substituting Eqns. S15.2–5 into Eqn. S15.1:

$$E_{cat} = \left[\frac{RT}{(\beta_{C-H}+\beta_{O-1})F}\ln\left(\frac{2\left(\frac{k_BT}{h}e^{\frac{-\Delta G^{\ddagger}_{O-1}}{RT}}e^{\frac{\beta_{O-1}FE^{0}_{0-1}}{RT}}\right)\left(e^{\frac{-\Delta G_{ads,O_2}}{RT}}\right)}{\left(\frac{k_BT}{h}e^{\frac{-\Delta G^{\ddagger}_{C-H}}{RT}}e^{\frac{-\beta_{C-H}FE^{0}_{C-H}}{RT}}\right)\left(e^{\frac{-\Delta G_{ads,GNA}}{RT}}\right)}\right)\right] + \left[\frac{RT}{(\beta_{C-H}+\beta_{O-1})F}\right]\ln\left(\frac{a_{O_2}}{a_{GNA}}\right) \quad \text{(S15.6)}$$

Simplifying Eqn. S15.6:

$$E_{cat} = \left[\frac{RT}{(\beta_{C-H}+\beta_{O-1})F}\left(\frac{-\Delta G^{\ddagger}_{O-1}-\Delta G_{ads,O_2}+\Delta G^{\ddagger}_{C-H}+\Delta G_{ads,GNA}+\beta_{O-1}FE^{0}_{0-1}+\beta_{C-H}FE^{0}_{C-H}}{RT} + \ln\left(2\frac{a_{O_2}}{a_{GNA}}\right)\right)\right] \quad \text{(S15.7)}$$

Next, decoupling all free energies into their enthalpy and entropy components:

$$E_{cat} = \left[\frac{RT}{(\beta_{C-H}+\beta_{O-1})F}\left(\frac{-\Delta H^{\ddagger}_{O-1}-\Delta H_{ads,O_2}+\Delta H^{\ddagger}_{C-H}+\Delta H_{ads,GNA}+\beta_{O-1}FE^{0}_{0-1}+\beta_{C-H}FE^{0}_{C-H}}{RT} + \left(\frac{\Delta S^{\ddagger}_{O-1}+\Delta S_{ads,O_2}-\Delta S^{\ddagger}_{C-H}-\Delta S_{ads,GNA}}{R} + \ln\left(2\frac{a_{O_2}}{a_{GNA}}\right)\right.\right.\right.$$

(S15.8)

Simplifying Eqn. S15.8:

$E_{cat} =$

$$\left[\left(\frac{-\Delta H^{\ddagger}_{O-1} - \Delta H_{ads,O_2} + \Delta H^{\ddagger}_{C-H} + \Delta H_{ads,GNA}}{F(\beta_{C-H} + \beta_{O-1})} + \frac{\beta_{O-1} E^{0}_{0-1} + \beta_{C-H} E^{0}_{C-H}}{(\beta_{C-H} + \beta_{O-1})}\right) + T\left(\frac{\Delta S^{\ddagger}_{O-1} + \Delta S_{ads,O_2} - \Delta S^{\ddagger}_{C-H} - \Delta S_{ads,GNA}}{(\beta_{C-H} + \beta_{O-1})F} + \frac{R}{(\beta_{C-H} + \beta_{O-1})F} \ln\left(2\,\frac{a_{O}[illegible]}{a_{G}[illegible]}\right.\right.\right.$$

(S15.9)

The first set of grouped terms are approximately constant with temperature, and the second set of grouped terms depend linearly on temperature. Therefore, the partial derivative of E_{cat} with respect to temperature ($\partial E_{cat}/\partial T$) is:

$$\frac{\partial E_{cat}}{\partial T} = \left(\frac{\Delta S^{\ddagger}_{O-1} + \Delta S_{ads,O_2} - \Delta S^{\ddagger}_{C-H} - \Delta S_{ads,GNA}}{(\beta_{C-H} + \beta_{O-1})F} + \frac{R}{(\beta_{C-H} + \beta_{O-1})F} \ln\left(2\,\frac{a_{O_2}}{a_{GNA}}\right)\right) \qquad \text{(S15.10)}$$

Analytical Expression Deconvoluting the Apparent Activation Enthalpies and Entropies

Following a similar analysis, the GNA oxidation rates ($-r_{GNA}$) are described by (Eqn. S14.48):

$$\frac{-r_{GNA}}{[L]} = \frac{2k^{0'}_{O-1} K_{O_2} a_{O_2} e^{\left(\frac{\beta_{C-H}}{\beta_{C-H} + \beta_{O-1}}\right)}}{1 + \left(\frac{2k^{0'}_{O-1} K_{O_2} K^{0}_{Pt}}{k^{0'}_{C-H} K_{GNA}} e^{\left(\frac{1}{\beta_{C-H} + \beta_{O-1}}\right)}\right)\left(\frac{a_{O_2}}{a_{GNA}}\right) + K_{O_2} a_{O_2} + K_{GNA} a_{GNA}} \qquad \text{(S15.11)}$$

Dividing both sides by temperature and taking the natural logarithm of Eqn. S15.11:

$$\ln\left(\frac{\frac{-r_{GNA}}{[L]}}{T}\right) = \ln\left(\frac{2k^{0'}_{O-1} K_{O_2} a_{O_2} e^{\left(\frac{\beta_{C-H}}{\beta_{C-H} + \beta_{O-1}}\right)}}{T}\right) - \ln\left(1 + \left(\frac{2k^{0'}_{O-1} K_{O_2} K^{0}_{Pt}}{k^{0'}_{C-H} K_{GNA}} e^{\left(\frac{1}{\beta_{C-H} + \beta_{O-1}}\right)}\right)\left(\frac{a_{O_2}}{a_{GNA}}\right) + K_{O_2} a_{O_2} + K_{GNA} a_{GNA}\right)$$

(S15.12)

For K^{0}_{Pt}, the van ‘t Hoff relation connects the equilibrium constant to its adsorption free energy:

$$K^{0}_{Pt} = e^{\frac{-\Delta G_{Pt.1}}{RT}} \qquad \text{(S15.13)}$$

Substituting Eqns. S15.2–5 and S15.13 into Eqn. S15.12:

$$\ln\left(\frac{\frac{-r_{GNA}}{[L]}}{T}\right) = \ln\left(2\,\frac{k_B}{h}\, e^{\frac{-\Delta G^{\ddagger}_{O-1}}{RT}} e^{\frac{\beta_{O-1} F E^{0}_{0-1}}{RT}} e^{\frac{-\Delta G_{ads,O_2}}{RT}} a_{O_2} e^{\left(\frac{\beta_{C-H}}{\beta_{C-H} + \beta_{O-1}}\right)}\right) - \ln\left(1 + \left(\frac{2\,\frac{k_B T}{h} e^{\frac{-\Delta G^{\ddagger}_{O-1}}{RT}} e^{\frac{\beta_{O-1} F E^{0}_{0-1}}{RT}} e^{\frac{-\Delta G_{ads,O_2}}{RT}} e^{\frac{-\Delta G_{Pt.1}}{RT}}}{\frac{k_B T}{h} e^{\frac{-\Delta G^{\ddagger}_{C-H}}{RT}} e^{\frac{-\beta_{C-H} F E^{0}_{C-H}}{RT}} e^{\frac{-\Delta G_{ads,GNA}}{RT}}} e^{\left(\frac{1}{\beta_{C-H} + \beta_{O-1}}\right)}\right)\left(\frac{a_{O_2}}{a_{GNA}}\right) + e^{\frac{-\Delta G_{ads,O_2}}{RT}} a_{O_2} + e^{\frac{-\Delta G_{ads,GNA}}{RT}} a_{GNA}\right)$$

(S15.14)

Simplifying Eqn. S15.14:

$$\ln\left(\frac{\frac{-r_{GNA}}{[L]}}{T}\right) = \ln\left(2\,\frac{k_B}{h}\,a_{O_2}\right) + \left(\frac{\beta_{C-H}}{\beta_{C-H}+\beta_{O-1}}\right) + \frac{-\Delta G^{\ddagger}_{O-1} - \Delta G_{ads,O_2} + \beta_{O-1} F E^{0}_{0-1}}{RT} -$$
$$\ln\left(1 + \left(\frac{2e^{\frac{-\Delta G^{\ddagger}_{O-1}}{RT}} e^{\frac{\beta_{O-1} F E^{0}_{0-1}}{RT}} e^{\frac{-\Delta G_{ads,O_2}}{RT}} e^{\frac{-\Delta G_{Pt.1}}{RT}}}{e^{\frac{-\Delta G^{\ddagger}_{C-H}}{RT}} e^{\frac{-\beta_{C-H} F E^{0}_{C-H}}{RT}} e^{\frac{-\Delta G_{ads,GNA}}{RT}}} e^{\left(\frac{1}{\beta_{C-H}+\beta_{O-1}}\right)}\right)\left(\frac{a_{O_2}}{a_{GNA}}\right) + e^{\frac{-\Delta G_{ads,O_2}}{RT}} a_{O_2} + e^{\frac{-\Delta G_{ads,GNA}}{RT}} a_{GNA}\right)$$

(S15.15)

While Eqn. S15.15 cannot be simplified further, linear regression gives apparent rate constants in regimes the fractional coverage of HO$^{-}*$ approaches unity, as the most abundant surface intermediate (MASI).

Limiting Case: HO$^{-}*$ as the MASI

In this case, Eqn. S15.11 simplifies to:

$$\frac{-r_{GNA}}{[L]} \approx \frac{2k^{0\,\prime}_{O-1} K_{O_2} a_{O_2} e^{\left(\frac{\beta_{C-H}}{\beta_{C-H}+\beta_{O-1}}\right)}}{\left(\frac{2k^{0\,\prime}_{O-1} K_{O_2} K^{0}_{Pt}}{k^{0\,\prime}_{C-H} K_{GNA}} e^{\left(\frac{1}{\beta_{C-H}+\beta_{O-1}}\right)}\right)\left(\frac{a_{O_2}}{a_{GNA}}\right)} \quad \text{(S15.16)}$$

Furthermore, in this regime, Eqn. S15.15 becomes:

$$\ln\left(\frac{\frac{-r_{GNA}}{[L]}}{T}\right) = \ln\left(2\,\frac{k_B}{h}\,a_{O_2}\right) + \left(\frac{\beta_{C-H}}{\beta_{C-H}+\beta_{O-1}}\right) + \frac{-\Delta G^{\ddagger}_{O-1} - \Delta G_{ads,O_2} + \beta_{O-1} F E^{0}_{0-1}}{RT} -$$
$$\ln\left(\left(\frac{2e^{\frac{-\Delta G^{\ddagger}_{O-1}}{RT}} e^{\frac{\beta_{O-1} F E^{0}_{0-1}}{RT}} e^{\frac{-\Delta G_{ads,O_2}}{RT}} e^{\frac{-\Delta G_{Pt.1}}{RT}}}{e^{\frac{-\Delta G^{\ddagger}_{C-H}}{RT}} e^{\frac{-\beta_{C-H} F E^{0}_{C-H}}{RT}} e^{\frac{-\Delta G_{ads,GNA}}{RT}}} e^{\left(\frac{1}{\beta_{C-H}+\beta_{O-1}}\right)}\right)\left(\frac{a_{O_2}}{a_{GNA}}\right)\right) \quad \text{(S15.17)}$$

Simplifying Eqn. S15.17:

$$\ln\left(\frac{\frac{-r_{GNA}}{[L]}}{T}\right) = \ln\left(2\,\frac{k_B}{h}\,a_{O_2}\right) + \left(\frac{\beta_{C-H}}{\beta_{C-H}+\beta_{O-1}}\right) + \frac{-\Delta G^{\ddagger}_{O-1} - \Delta G_{ads,O_2} + \beta_{O-1} F E^{0}_{0-1}}{RT} - \ln\left(2\,\frac{a_{O_2}}{a_{GNA}}\right) - \left(\frac{1}{\beta_{C-H}+\beta_{O-1}}\right) +$$
$$\frac{\Delta G^{\ddagger}_{O-1} + \Delta G_{ads,O_2} - \beta_{O-1} F E^{0}_{0-1} + \Delta G_{Pt.1} - \Delta G^{\ddagger}_{C-H} - \beta_{C-H} F E^{0}_{C-H} - \Delta G_{ads,GNA}}{RT} \quad \text{(S15.18)}$$

$$\ln\left(\frac{\frac{-r_{GNA}}{[L]}}{T}\right) = \ln\left(\frac{k_B}{h}\,a_{GNA}\right) + \left(\frac{-\beta_{O-1}}{\beta_{C-H}+\beta_{O-1}}\right) + \frac{-\Delta G^{\ddagger}_{C-H} - \beta_{C-H} F E^{0}_{C-H} - \Delta G_{ads,GNA} + \Delta G_{Pt.1}}{RT} \quad \text{(S15.19)}$$

Next, decoupling all free energies into their enthalpy and entropy components:

$$\ln\left(\frac{\frac{-r_{GNA}}{[L]}}{T}\right) = \ln\left(\frac{k_B}{h}\,a_{GNA}\right) + \left(\frac{-\beta_{O-1}}{\beta_{C-H}+\beta_{O-1}}\right) + \left(\frac{-\Delta H^{\ddagger}_{C-H} - \beta_{C-H} F E^{0}_{C-H} - \Delta H_{ads,GNA} + \Delta H_{Pt.1}}{RT}\right) +$$
$$\left(\frac{\Delta S^{\ddagger}_{C-H} + \Delta S_{ads,GNA} - \Delta S_{Pt.1}}{R}\right) \quad \text{(S15.20)}$$

Finally, taking the partial derivative of Eqn. S15.20 with respect to inverse temperature gives the apparent activation enthalpy $\Delta H^{\ddagger}_{eff}$:

$$\frac{\partial\left(\ln\left(\frac{-r_{GNA}}{[L]}/T\right)\right)}{\partial(1/T)} = \frac{-\Delta H^{\ddagger}_{eff}}{R} = \left(\frac{-\Delta H^{\ddagger}_{C-H} - \beta_{C-H} F E^{0}_{C-H} - \Delta H_{ads,GNA} + \Delta H_{Pt.1}}{R}\right) \quad \text{(S15.21)}$$

In this limiting regime, $\Delta H^{\ddagger}_{eff}$ does not depend on $\Delta H^{\ddagger}_{O-1}$ or $\Delta H_{ads,O_2}$. On the free energy landscape, this reflects rates dominated by GNA oxidation half-reactions.

Combining E_{cat} and $-r_{GNA}$ to Deconvolute Activation Enthalpies and Entropies of O_2 Activation

Regression of all temperature dependencies on $-r_{GNA}$ against Eqn. S15.15 gives the first order GNA oxidation activation enthalpy ($\Delta H^{\ddagger}_{C-H} + \Delta H_{ads,GNA}$) and entropy ($\Delta S^{\ddagger}_{C-H} + \Delta S_{ads,GNA}$), provided in Figure 6c of the main text. Combining these values with the temperature dependencies on E_{cat} estimates the first order O_2 reduction activation enthalpy ($\Delta H^{\ddagger}_{O-1} + \Delta H_{ads,O_2}$) and entropy ($\Delta S^{\ddagger}_{O-1} + \Delta S_{ads,O_2}$) values which would otherwise be inaccessible in C-H bond scission limited kinetic regimes.

Rearranging Eqn. S15.10 isolates $\Delta S^{\ddagger}_{O-1} + \Delta S_{ads,O_2}$:

$$\left(\Delta S^{\ddagger}_{O-1} + \Delta S_{ads,O_2}\right) = \left(\Delta S^{\ddagger}_{C-H} + \Delta S_{ads,GNA}\right) + (\beta_{C-H} + \beta_{O-1})F\frac{\partial E_{cat}}{\partial T} - \ln\left(2\,\frac{a_{O_2}}{a_{GNA}}\right) \quad \text{(S15.22)}$$

In the limit when β_{C-H} and β_{O-1} remain constant with temperature (approximating their sum as 0.6), substituting all kinetic parameters on the right side of Eqn. S15.22 gives the first order O_2 reduction activation entropy ($\Delta S^{\ddagger}_{O-1} + \Delta S_{ads,O_2}$ equals -208 ± 21 J (mol K)$^{-1}$). We note that enforcing a sum of β_{C-H} and β_{O-1} of unity significantly decreases the first order O_2 reduction activation entropy (-208 to -243 ± 21 J (mol K)$^{-1}$).

At steady state, the consumption rates of GNA and O_2 must equate, normalized by their stoichiometry.

$$-r_{GNA} = -2r_{O_2} \quad \text{(S15.23)}$$

Following the same derivation as Section S14, Eqn. S15.23 gives Eqn. S15.1, and thus Eqn. S15.7:

$$E_{cat} = \left[\frac{RT}{(\beta_{C-H}+\beta_{O-1})F}\left(\frac{-\Delta G^{\ddagger}_{O-1}-\Delta G_{ads,O_2}+\Delta G^{\ddagger}_{C-H}+\Delta G_{ads,GNA}+\beta_{O-1}FE^{0}_{0-1}+\beta_{C-H}FE^{0}_{C-H}}{RT} + \ln\left(2\,\frac{a_{O_2}}{a_{GNA}}\right)\right)\right] \quad \text{(S15.24)}$$

Rearranging Eqn. S15.24:

$$\left((\beta_{C-H} + \beta_{O-1})FE_{cat} - RT\ln\left(2\,\frac{a_{O_2}}{a_{GNA}}\right)\right) = \left(\Delta G^{\ddagger}_{C-H} + \Delta G_{ads,GNA} + \beta_{C-H}FE^{0}_{C-H}\right) - (\Delta G^{\ddagger}_{O-1} + \Delta G_{ads,O_2} - \beta_{O-1}FE^{0}_{0-1}) \quad \text{(S15.25)}$$

Combining the measured E_{cat} and O_2/GNA values (Figure 6b of the main text) with the GNA oxidation activation free energies (Figure 6a of the main text) in Eqn. S15.25 gives the first order activation free energies for O_2 reduction half-reactions (summation of $\Delta G^{\ddagger}_{O-1}$, $\Delta G_{ads,O_2}$, and $\beta_{O-1}FE^{0}_{0-1}$) at each

temperature. With both activation free energies (Eqn. S15.25) and entropies (Eqn. S15.22) known, trivial calculations give the first order activation enthalpies of O_2 reduction.

S16. GNA Oxidation Transient Rate and Potential Profiles with Varying O_2 Shutoff Times

Figure S13 shows GNA oxidation rates and catalyst open circuit potentials (E_{cat}) as a function of time on stream during O_2 shutoff experiments at varying O_2 shutoff times of 1 hour (a) and 0.5 hours (b). In both cases, E_{cat} decreases by over 500 mV within the first 10 minutes of the O_2 shutoff. Furthermore, upon reintroducing O_2, GNA oxidation rates are higher than their initial values, increasing by at least 80%, consistent with the longer O_2 shutoff experiments in Figure 7 of the main text. We note that the 1-hour O_2 shutoff experiment was performed using a fresh Ag/AgCl reference electrode, a slightly higher O_2 pressure (approximately 120 kPa O_2), and a slightly lower GNA concentration (0.1 M instead of 0.12 M), leading to the 70 mV higher E_{cat} values when compared to the 0.5-hour O_2 shutoff experiment.

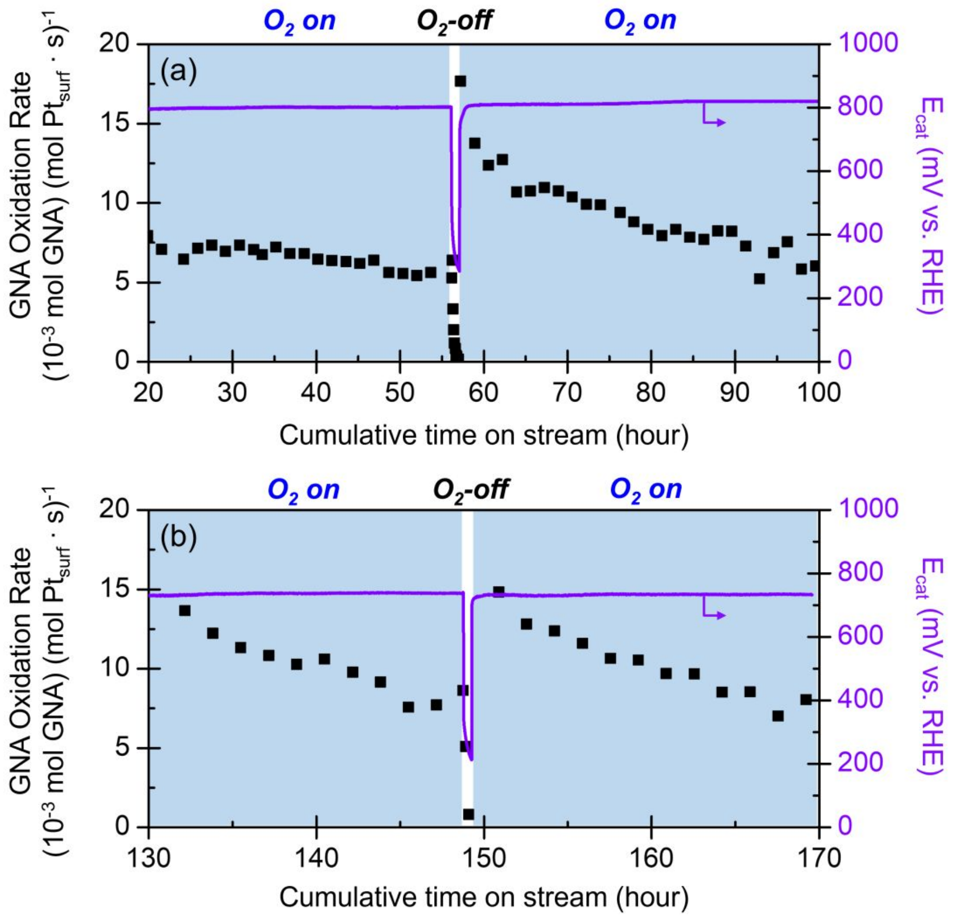

Figure S13. GNA oxidation rates (■) and catalyst open circuit potentials (purple) versus time on stream with O_2 (blue shaded) or without O_2 (Ar, unshaded) on Pt/C catalysts, with O_2 shutoff times of (a) 1 hour and (b) 0.5 hours. 0.10–0.12 M GNA (30 cm^3_{liquid} h^{-1}), 100 kPa gas (3000 cm^3_{STP} h^{-1}), 353 K, 0.5 g Pt/C.

S17. Optimizing Integral Reactor Productivity with Periodic O_2 Shutoffs

During reactor startup and after periodic O_2 shutoffs, GNA oxidation turnover rates can be described by a piecewise function (modeling the first deactivation regime provided in Section S6):

$$-r_{GNA}(t_{ox}) = \begin{cases} t_{ox} < t_{ind}: & r_0 e^{-t_{ox}/\tau_d} \\ t_{ox} \geq t_{ind}: & r_{ss} \end{cases} \tag{S17.1}$$

where t_{ox} represents the time spent with flowing O_2 during periodic operations. For illustrations, we model the O_2 shutoff of Figure S13b (O_2 shutoff time of 0.5 hours, 0.12 M GNA, 1 bar O_2, at 353 K), where induction times t_{ind} are approximately 23 hours, initial rates r_0 are 8.4×10^{-3} mol (g-atom-Pt_{surf} s)$^{-1}$, steady state rates r_{ss} are 4.7×10^{-3} mol (g-atom-Pt_{surf} s)$^{-1}$, and deactivation time constants τ_d are approximately 39 hours. The GNA oxidation rate depends sensitively on t_{ox} at a fixed O_2 shutoff time (t_{off}), with time-averaged turnover rates described by Eqn. S14.2:[9]

$$-r_{GNA,averaged} = \frac{\left(\int_{t=0}^{t_{ox}} -r_{GNA}(t_{ox})dt\right)}{t_{ox}+t_{off}} \tag{S17.2}$$

The productivity, in terms of the total number of catalytic turnovers in one day (in units of mol (g-atom-Pt_{surf})$^{-1}$), equals $-r_{GNA,averaged}$ multiplied by 24 hours. Figure S14 shows the total number of catalytic turnovers in 24 hours as a function of t_{ox}, where the maximum productivity occurs when $t_{ox} = 6.1$ hours, and the turnover numbers are approximately 50% higher than the steady state equivalents.

We note that high reactor heat loads upon introducing O_2 would reduce the integral productivity in GNA oxidation and limit this programmable approach at higher conversion, where the exothermic GNA oxidation reactions can cause local heat gradients near the catalyst surfaces that sinter or leach Pt as well as form byproducts.

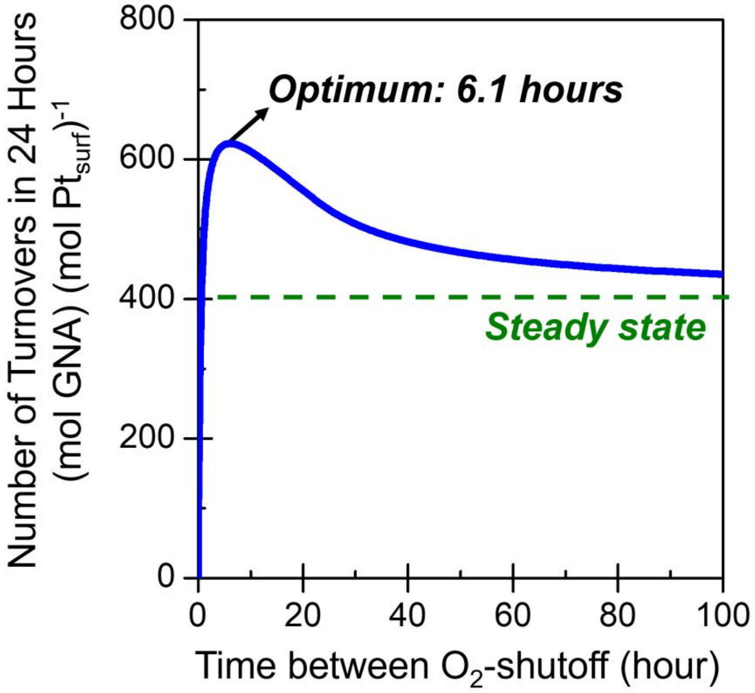

Figure S14. Dependence of the number of GNA oxidation catalytic turnovers in 24 hours, as a function of the time between 0.5-hour O_2 shutoffs, for a model at 0.12 M GNA, 100 kPa O_2 or Ar, and 353 K.

S18. References

(1) Bergeret, G.; Gallezot, P. Particle Size and Dispersion Measurements. In *Handbook of Heretogeneous Catalysis*; Wiley-VCH, 2008; Vol. 2, pp 738–765. https://doi.org/10.1002/9783527610044.hetcat0038.

(2) Cai, G.; Broomhead, W. T.; Chin, Y.-H.; Cai, H. Advanced Kinetic and Titration Strategies for Assessing the Intrinsic Kinetics on Oxide and Sulfide Catalysts. *Topics in Catalysis* **2023**, *66* (16), 1102–1119. https://doi.org/10.1007/s11244-023-01849-w.

(3) Conway, B. E.; Jerkiewicz, G. Relation of Energies and Coverages of Underpotential and Overpotential Deposited H at Pt and Other Metals to the 'Volcano Curve' for Cathodic H_2 Evolution Kinetics. *Electrochimica Acta* **2000**, *45* (25), 4075–4083. https://doi.org/10.1016/S0013-4686(00)00523-5.

(4) Fischer, P. J. Electrocatalytic Hydrogenation of Oxygenated Compounds in Aqueous Phase – Investigation of Fundamental Steps at the Electrode/Electrolyte Interface, Technische Universität München, **2022**. https://mediatum.ub.tum.de/node?id=1684645.

(5) Singh, N.; Sanyal, U.; Fulton, J. L.; Gutiérrez, O. Y.; Lercher, J. A.; Campbell, C. T. Quantifying Adsorption of Organic Molecules on Platinum in Aqueous Phase by Hydrogen Site Blocking and in Situ X-Ray Absorption Spectroscopy. *ACS Catalysis* **2019**, *9* (8), 6869–6881. https://doi.org/10.1021/acscatal.9b01415.

(6) Adams, J. S.; Kromer, M. L.; Rodríguez-López, J.; Flaherty, D. W. Unifying Concepts in Electro- and Thermocatalysis toward Hydrogen Peroxide Production. *Journal of the American Chemical Society* **2021**, *143* (21), 7940–7957. https://doi.org/10.1021/jacs.0c13399.

(7) Mars, P.; van Krevelen, D. W. Oxidations Carried out by Means of Vanadium Oxide Catalysts. *Chemical Engineering Science* **1954**, *3*, 41–59. https://doi.org/10.1016/S0009-2509(54)80005-4.

(8) Davis, S. E.; Ide, M. S.; Davis, R. J. Selective Oxidation of Alcohols and Aldehydes over Supported Metal Nanoparticles. *Green Chemistry* **2012**, *15* (1), 17–45. https://doi.org/10.1039/C2GC36441G.

(9) Missen, R. W.; Mims, C. A.; Saville, B. A. Batch Reactors. In *Introduction to Chemical Reaction Engineering and Kinetics*; John Wiley & Sons, **1999**; pp 294–316.
